# Identification of sequence changes in myosin II that adjust muscle contraction velocity

**DOI:** 10.1371/journal.pbio.3001248

**Published:** 2021-06-10

**Authors:** Chloe A. Johnson, Jake E. McGreig, Sarah T. Jeanfavre, Jonathan Walklate, Carlos D. Vera, Marta Farré, Daniel P. Mulvihill, Anthony J. Baines, Martin Ridout, Leslie A. Leinwand, Mark N. Wass, Michael A. Geeves

**Affiliations:** 1 School of Biosciences, University of Kent, Canterbury, United Kingdom; 2 BioFrontiers Institute and Department of Molecular, Cellular and Developmental Biology, University of Colorado Boulder, Colorado, United States of America; 3 School of Mathematics, Statistics and Actuarial Science, University of Kent, Canterbury, United Kingdom; University of Bergen, NORWAY

## Abstract

The speed of muscle contraction is related to body size; muscles in larger species contract at slower rates. Since contraction speed is a property of the myosin isoform expressed in a muscle, we investigated how sequence changes in a range of muscle myosin II isoforms enable this slower rate of muscle contraction. We considered 798 sequences from 13 mammalian myosin II isoforms to identify any adaptation to increasing body mass. We identified a correlation between body mass and sequence divergence for the motor domain of the 4 major adult myosin II isoforms (β/Type I, IIa, IIb, and IIx), suggesting that these isoforms have adapted to increasing body mass. In contrast, the non-muscle and developmental isoforms show no correlation of sequence divergence with body mass. Analysis of the motor domain sequence of β-myosin (predominant myosin in Type I/slow and cardiac muscle) from 67 mammals from 2 distinct clades identifies 16 sites, out of 800, associated with body mass (p_adj_ < 0.05) but not with the clade (p_adj_ > 0.05). Both clades change the same small set of amino acids, in the same order from small to large mammals, suggesting a limited number of ways in which contraction velocity can be successfully manipulated. To test this relationship, the 9 sites that differ between human and rat were mutated in the human β-myosin to match the rat sequence. Biochemical analysis revealed that the rat–human β-myosin chimera functioned like the native rat myosin with a 2-fold increase in both motility and in the rate of ADP release from the actin–myosin crossbridge (the step that limits contraction velocity). Thus, these sequence changes indicate adaptation of β-myosin as species mass increased to enable a reduced contraction velocity and heart rate.

## Abstract 2

Heart and skeletal muscles of larger mammals contract more slowly than smaller ones. This study identifies amino acid changes in myosin isoforms that correlate with species size; mutating the residues in human β-myosin to match the rat sequence at these positions increased its in vitro velocity to that of the rat protein.

## Introduction

Proteins can adapt over time, tuning their function to the specific needs of the organisms in which they are expressed. That is, the same protein expressed in different organisms (orthologues) may have distinct properties to better suit the needs of each organism. This is well established for muscle contraction where the maximum shortening velocity, V_0_, is a property of the myosin isoform expressed in the tissue. V_0_ varies more than 5-fold for muscles expressing the same myosin isoform in different mammals. For example, the V_0_ of rat and human Type I/β-cardiac myosin (hereafter referred to as β-myosin) are 1.42 and 0.33 μm s^−1^ half sarcomere^−1^, respectively [1]. Across a range of mammals, V_0_ for each muscle myosin isoform is inversely related to the size of the mammal; larger mammals have slower contracting muscles [1,2]. This can most commonly be observed in cardiac tissue where heart rate (related to the contraction velocity, see below) varies widely and is slower for larger mammals [3].

Since V_0_ is a property of the myosin isoform expressed in the tissue, there are expected to be changes in the myosin sequence that have resulted in a myosin with the needed velocity. However, changes in sequence may be occurring for reasons other than adjusting the velocity. Here, we ask if it is possible to define which sequence changes tune V_0_ for an individual myosin isoform.

Several attempts have been made to identify the sequence changes associated with changes in the myosin isoforms and the associated changes in shortening velocity. These used the relatively small numbers of sequences available at the time or considered the differences between paralogues of muscle myosin IIs. The studies focused on nonconservative substitutions in sequence [4] or on the variable surface loops [2,5] and identified areas of interest, but did not locate specific residues nor how they might influence contraction velocity. Large numbers of mammalian myosin sequences are now available from various genome projects. Using this large data set, we test the hypothesis that muscle myosin-II isoforms from mammals have adaptations in protein sequence associated with mean body mass and V_0_.

We have examined 798 mammalian myosin II sequences, from 13 myosin II isoforms and find that the myosin isoforms found in adult sarcomeric muscle (Type II fast muscle isoforms IIa, IIb, and IIx and β; see Table 1 for definition of isoforms) have a much higher variation in sequence than the non-muscle myosins (non-muscle A [NMA] and non-muscle B [NMB]). This is consistent with an adaptation of contraction velocity to body mass occurring only in the adult muscle myosins and not in the non-muscle cellular myosin which are unaffected by overall species size.

**Table 1:** Myosin II isoforms considered.

Gene name	Heavy chain	Muscle type	Isoform	Short name	No complete sequences	Motor		Tail	
**Major adult sarcomeric muscle myosins**						R^2^	Gradient (%/log(kg))	R^2^	Gradient (%/log(kg))
*MYH1*	MyHC-1	Fast	Skeletal IIb	IIb	51	0.48	−0.52 ± 0.20	0.58	−0.75 ± 0.12
*MYH2*	MyHC-2	Fast	Skeletal IIa	IIa	77	0.44	−0.53 ± 0.10	0.35	−0.43 ± 0.11
*MYH4*	MyHC-4	Fast	Skeletal IID/X	IIx	41	0.84	−0.84 ± 0.11	0.85	−0.68 ± 0.09
*MYH6*	MyHC-6	Cardiac	α-cardiac	α	65	0.3	−0.36 ± 0.29	0.35	−0.31 ± 0.11
*MYH7*	MyHC-7	Cardiac/slow	β-cardiac	β	67	0.63	−0.72 ± 0.11	0.03	−0.06 ± 0.16
**Specialist adult sarcomeric myosins**									
*MYH13*	MyHC-13	Fast	Extraocular	EXOC	60	0.18	−0.32 ± 0.10	0.21	−0.72 ± 0.2
*MYH7b*	MyHC-7b	Slow	Slow tonic	SlowT	72	0.05	−0.08 ± 0.06	0.03	−0.063 ± 0.06
**Developmental muscle isoforms**									
*MYH3*	MyHC-3	Developmental	Embryonic	EMB	60	0.13	−0.09 ± 0.04	0.22	−0.31 ± 0.12
*MYH8*	MyHC-8	Developmental	Perinatal	PERI	54	0.01	−0.04 ± 0.11	0.21	−0.28 ± 0.12
**Non sarcomeric myosins**									
*MYH9*	MyHC-9	Non-muscle	Non-muscle A	NMA	59	0.03	−0.033 ± 0.036	0.14	−0.14 ± 0.06
*MYH10*	MyHC-10	Non-muscle	Non-muscle B	NMB	69	0.11	0.042 ± 0.022	0.09	−0.08 ± 0.05
*MYH11*	MyHC-11	Smooth muscle	Smooth muscle	SM	71	0.52	−0.37 ± 0.07	0.08	−0.17 ± 0.09
*MYH14*	MyHC-14	Non-muscle	Non-muscle C	NMC	52	0.10	−0.21 ± 0.11	0.203	−0.65 ± 0.35

MyHC 13 and 7b are labelled “specialised” because unlike the other sarcomeric forms they are not found alone in a specific muscle type but only in combination with other isoforms.

We go on to examine in greater detail the sequence differences within the more widely studied mammalian β-myosin (MYH7, expressed in slow Type I and cardiac muscle) to establish if it is possible to define a relationship between amino acid sequence and velocity of contraction. We examine 67 mammalian β-myosin sequences from 2 clades and identify a group of 16 amino acids which have the strongest association with the size of the mammal and not the clade. Four are in the hypervariable regions (Loop2 and the N-terminus). Of the 12 remaining amino acids, 9 differ between human and rat β-myosin. We test our hypothesis that these 9 residues influence V_0_ through the construction and subsequent biochemical characterisation of a rat–human β-myosin chimera (hereafter referred to as chimera) in which the 9 rat residues are exchanged into the human β-myosin.

## Results

All myosin II isoforms contain a motor domain and a tail domain. The N-terminal globular motor domain (approximately 800 amino acids) contains all of the requirements for motor activity, while the carboxyl-terminal tail domain (approximately 1,200 amino acids, almost entirely a single α helix) drives dimerisation and assembly into myosin filaments. Here, we focus on the motor domain but also report a summary of a similar analysis of the tail domain.

Our analysis considered 13 different mammalian myosin II isoforms (Table 1), the 5 main adult sarcomeric muscle isoforms (3 fast muscle—IIa, IIb, and IIx and 2 cardiac isoforms, α and β, also known as the slow skeletal isoform), 2 relatively rare adult sarcomeric forms (extraocular and slow tonic), 2 developmental isoforms (perinatal and embryonic), a smooth muscle isoform, and 3 non-muscle isoforms (NMA, NMB, and non-muscle C [NMC]). The non-muscle isoforms provide a negative control, as they act only at the cellular level and are therefore unlikely to be influenced by body mass. We identified all available complete myosin II sequences of these 13 myosin isoforms (see Materials and methods; S2 Table). This resulted in a total of 798 sequences, with an average of 61 sequences per isoform (range 41 to 74 sequences; Table 1).

### Adaptation of the myosin II motor domain to increasing body mass

**Fig 1:**
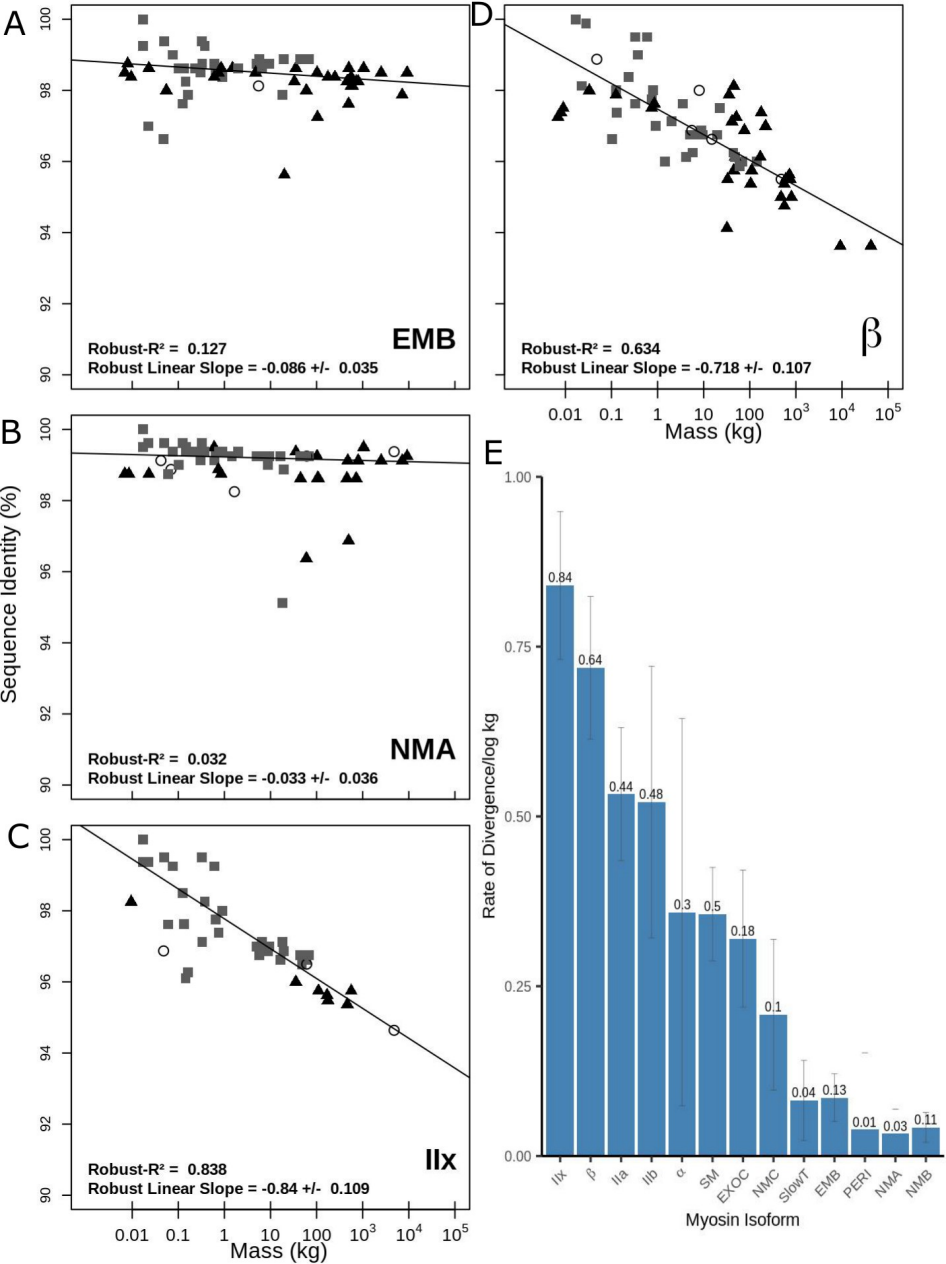
Sequence Identity (%ID) vs. Mass (kg) for myosin II motor domains. **(A–D)** Sequence Identity (%ID) vs. Mass (kg) for the motor domains of 4 myosin II isoforms: EMB, NMA, β-myosin, and IIx. Symbols indicate the clade that each species belongs to: grey squares (Euarchontoglires), black triangles (Laurasiatheria), and clear circles (Afrotheria and Metatheria). Each plot has been fitted with a robust linear regression. Sequence identity is pairwise to the mouse. The R^2^ value and slope are shown on each plot. **(E)** The relationship between body mass and sequence divergence for the 13 myosin II motor domains. Values above the bar are the R^2^ values. The error bars show the error of the gradient. Raw data files are available at Figshare. EMB, embryonic; NMA, non-muscle A; NMB, non-muscle B; NMC, non-muscle C.

To consider how the motor domain of myosin II isoforms may have adapted to increasing body mass, we investigated whether there was a correlation between sequence divergence and body mass. Mass values were collected from the literature, and we report the arithmetic mean of the range of values reported for each species. The range of masses covers more than 6 orders of magnitude from 6 g to 10,000 kg (S1 Table). Being one of the smallest species, the mouse was selected as a reference. The sequence identity of each other species with the mouse sequence was plotted against the species body mass (see Fig 1, Table 1, and S1 Fig). The analysis can alternatively be done using the myosin sequence from largest species, but this is not always the same for each myosin II data set. We therefore used the commonly available mouse sequences throughout. Sequence identity rather than conservation was used because within each myosin isoform, a low level of divergence was expected (>95% identity). As such, even conservative changes of amino acids that tune the function of the protein may be relevant to adaptation to body mass. For example, a 2-fold change in a rate constant that controls shortening velocity would according to transition state theory require only a change of the order of 1 kcal/mol in the activation energy. This is comparable to a single hydrogen bond or van der Waals interaction (see S1 Text and [6,7]).

In Fig 1, the sequence identity versus mass is plotted for 4 representative myosin motor domains: the slow/cardiac β-myosin, the fast muscle IIx, the non-muscle IIa, and the embryonic isoforms. Plots for the other 9 isoforms are presented in S1 Fig. The plots in Fig 1 show that both adult sarcomeric forms β and IIx have a strong correlation with body mass (R^2^ = 0.63 and 0.838, respectively) with gradients of −0.72 and −0.84 percent divergence per log kg. The data for all 13 isoforms are summarised in Fig 1E and Table 1. Based on the rate of divergence with body mass, the 13 myosin II isoforms form 3 groups. The first group with gradients >0.5% per log kg body mass contains 4 of the 5 main adult sarcomeric muscle isoforms, IIb, β, IIa, and IIx isoforms, with the IIb isoform plots showing a wider scatter in data as indicated in the 20% to 40% error in the gradient value. For a myosin motor domain of approximately 800 amino acids, a gradient of >0.5% per log kg body mass means >4 amino acids change for each 10-fold increase in body mass. At the other extreme, 5 of the isoforms (slow tonic, embryonic, perinatal, NMA, and NMB) exhibit little divergence of sequence and no correlation with body mass (slope < 0.1, R^2^ < 0.13), i.e., <1 amino acid per log kg increase in mass.

The remaining 4 isoforms (α-myosin, smooth muscle, extraocular, and NMC) are intermediate between the 2 groups with a lower rate of sequence divergence with body mass (−0.21 to −0.36) with only the smooth muscle isoform having an R^2^ > 0.3 (0.498). Note the very large scatter in the data for α-cardiac myosin (S1D Fig gradient −0.36 ± 0.29).

A similar range of gradients (−0.75 to −0.05) and R^2^ values (0.85 to 0.016) were observed for the tail domains, but only the adult sarcomeric myosins had R^2^ values greater than 0.3 (Table 1, S1 Fig), and for these sarcomeric myosins, the gradients and R^2^ values were similar for the motor and tail domains. An intriguing exception was the β-myosin isoform, where there was no correlation between size and sequence variation in the tail (gradient −0.06%/log(kg), R^2^ 0.03), showing that the tail is far more highly conserved than the motor and more highly conserved than for other sarcomeric myosin tail domains.

Given that there may be some error associated with the species body mass, we tested what effect this may have on the results of this analysis. To do this, we randomly modified the body mass of each species such that they were varied in the range 80% to 120% of the average value being used. This was repeated 1,000 times, each time reperforming the analysis. Our results demonstrate that the results are stable to such errors (S1 Table).

**Fig 2:**
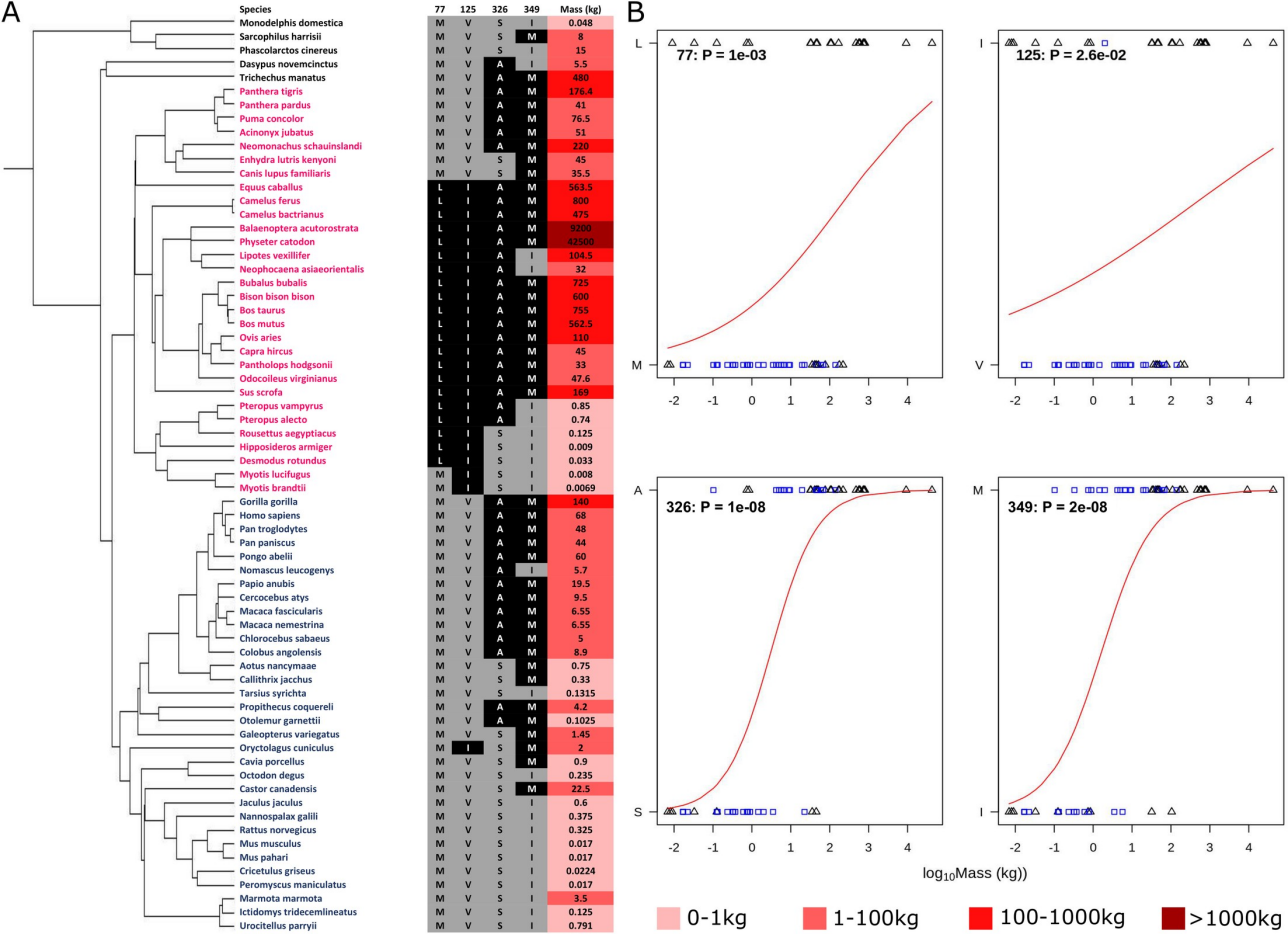
Residue mass transition plots for 4 representative amino acid sites. **(A)** Species tree of organisms used in this analysis is shown, with the amino acids present in each species at the 4 sites and the mass of the organism displayed adjacent to it. Darker reds in the mass column are indicative of a greater mass value. In this tree, species from the Euarchontoglires clade are highlighted blue, and species from Laurasiatheria are highlighted pink. **(B)** Binomial regression mapping the transition of the most frequent amino acid at positions in the motor region of β-myosin to the second most frequent amino acid at that position. The residue numbering is that of the human β-cardiac myosin. The blue squares are Euarchontoglires, and the triangles are Laurasiatheria. The *p*-value with each plot arises from a test of the null hypothesis that amino acid type is unrelated to mass. Raw data files are available at Figshare.

Species and gene trees were generated for the sarcomeric myosin isoforms (Fig 2A, S6 Fig). To exclude the possibility that the observations for adult sarcomeric myosins could simply be the result of phylogenetic relationships between the sequences, we used Felsenstein’s phylogenetically independent contrasts (PICs; [8]) method to exclude the phylogenetic relationship as a factor for the correlation between sequence divergence and body mass. For β-myosin, we still observe a significant correlation (*p* = 0.06 × 10^−4^). For the other 3 main adult isoforms (IIa, IIb, and IIx) where a significant correlation had been observed, only IIx was significant (*p* = 0.009), while IIa and IIb were not (*p* = 0.54 and 0.1, respectively). We also performed the equivalent analysis for the tail domain data (S1 Table) and observed a *p*-value of 0.29 for the β-myosin, indicating that variation in the tail domain is explained by phylogenetic relationships.

### Adaptation of the β-myosin motor domain to reduce contraction velocity as species size increased

Of the adult sarcomeric myosin isoforms, β-myosin is unique as it is the only slow muscle myosin, and it is expressed in few tissues, primarily slow, Type 1 muscle, and in cardiac muscle (it is the dominant isoform expressed in the ventricle the heart of mammals larger than 1 kg), and, as result of both of these, it performs the same specific function in different species. The other striated muscle isoforms are expressed in multiple tissues and may be involved in multiple different functions.

The negative correlation between species heart rate and body mass is well established across a wide range of species [3]. Given our observation that β-myosin shows a strong association of sequence change with body mass, it is possible that there has been selection pressure on this isoform to enable a change in contraction velocity, which is associated with the change in heart rate as body mass has increased. However, the observation of small correlation between sequence and body mass for the α isoform implies that this relationship between body mass and sequence, if present, is less dominant for α than for β. It is possible that the role of β as the only slow skeletal muscle isoform is more significant in defining the relationship to body mass than its role in cardiac muscle. This would make it similar to the fast skeletal muscle isoforms (IIa, IIb, and IIx) which show strong dependence of sequence on body mass.

In addition, the rate constant controlling ADP release is easily measured for β-myosin and limits contraction velocity for this isoform (Table 2 and references therein). Therefore, all further analyses focus on variation in β-myosin.

**Table 2:** The relationship between the predicted and measured parameters for 4 slow/beta cardiac myosin isoforms.

Slow muscle/β-cardiac myosin	Measured		Predicted τ (sec)		
	k_-ADP_ (s^−1^)	V_0_ (μm/sec/half sarcomere)	τ_ADP_ = 1/k_-ADP_ (msec)	τ_Vo_ = d/V_0_ (msec)	Ratio τ_ADP_/τ_Vo_
**Rat**	119	1.42	8.4	7.04	1.19
**Rabbit**	63	0.67	15.9	14.9	1.06
**Human**	30*	0.33	33	30.3	1.08
**Cow**	27	0.27	37	37.0	1.00

In terms of the actin myosin crossbridge cycle, the dominant model proposes that the maximum velocity (V0) is limited by the lifetime of the strongly attached force holding state (τ), the “detachment limited model” ([9], V0 = d/τ, where d is the working stroke of the crossbridge; assumed here to be 5 nm [7]). For the mammalian, β-myosin isoform, it is well established that τ is defined by the rate constant controlling ADP release k-ADP = 1/τ [10–12]. Thus, values of k-ADP measured using myosin motor domains isolated from β-cardiac/slow muscle of a mammal predict remarkably accurately the maximum shortening velocity of a muscle fibre taken from the same tissues.

Experimental data were collected at 100 mM KCl and 12°C.

k-_ADP_ values for bovine, rabbit, and human are from Deacon et al. [10], the rat from this study. * the value for human k_-ADP_ at 12°C, was estimated from an Arrhenius plot of values between 20 and 10°C. These values are consistent with rat and porcine β data collected using the 2-headed myosin fragment, heavy meromyosin carried out at 100 mM KCl and 15°C.

V_o_ data for rat, rabbit, and human are from Pellegrino et al. [1], and bovine is from Toniolo et al. [13].

**Fig 3:**
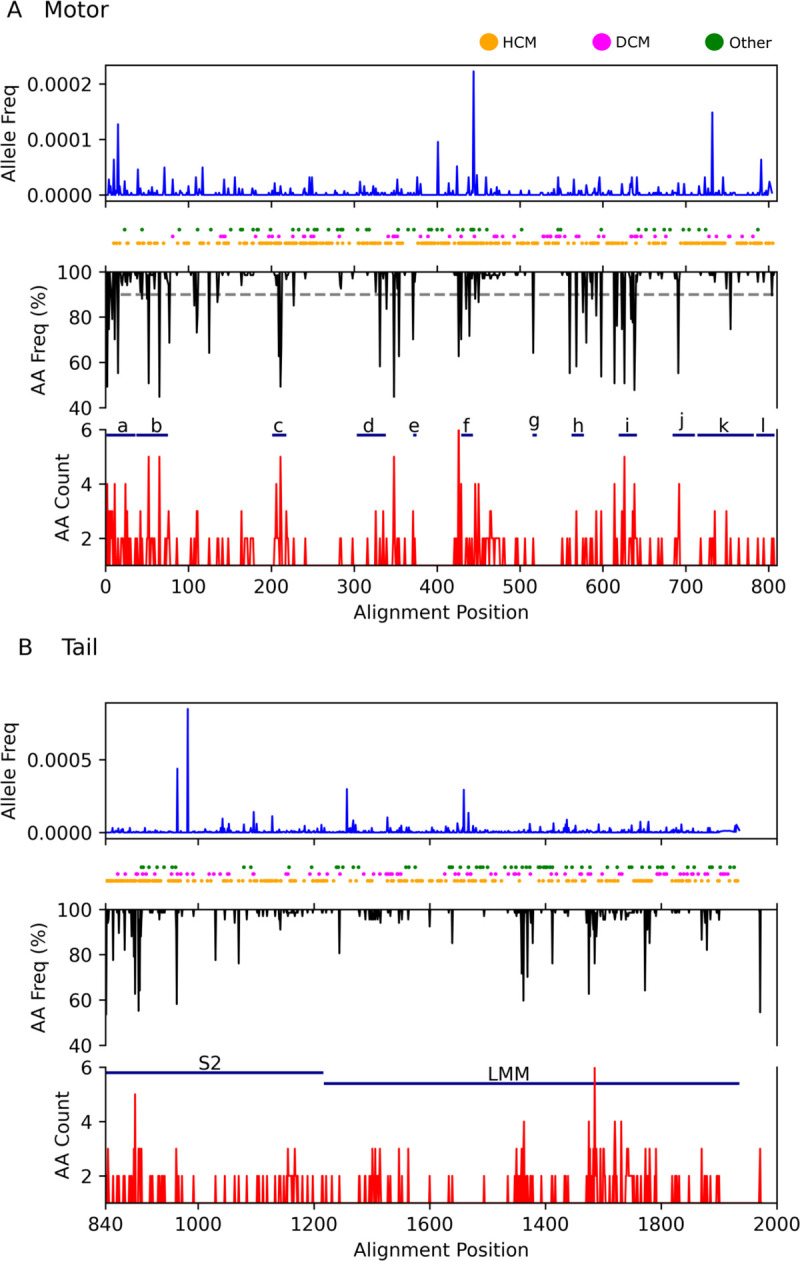
Location of human variation, cardiomyopathy-associated variants, and non-conserved residues for the β isoform. Blue lines show the frequency of missense variation present in the gnomAD. Circles indicate the residue position of variants associated with cardiomyopathy (HCMs in orange, DCMs in magenta, and other in green [14]). Black lines show the frequency with which the consensus amino acid occurs at each residue position in the sequence. Red lines show the number of different amino acids occurring at that position in the sequence (minimum 1). The dashed grey lines indicate the position at which more than 10% of sequences contain an alternate amino acid. Key functional areas of the sequence as listed Motor domain: a. N-terminus 1–36, b. SH3 domain 37–75, c. Loop 1 201–215, d. *Drosophila* exon 7 region 300–335, e. Loop 4 368–372, f. Helix-O 426–440, g. Relay helix/loop, h. Loop 3 558–573, i. Loop 2 615–637, j. SH helices 680–707, k. Converter 710–778, l. IQ 781–810. B. Tail. S2, subfragment 2 coiled-coil; LMM, light meromyosin filament forming region. Raw data files are available at Figshare. DCM, dilated cardiomyopathy; gnomAD, Genome Aggregation Database; HCM, hypertrophic cardiomyopathy.

The location of sequence changes in the motor and tail domains were examined to evaluate if particular structural features of the domains are especially variable (Fig 3). For each residue, we considered the frequency of the consensus amino acid (black lines) and the number of different amino acids present at each position (red lines). In the β-myosin motor domain, of the 800 amino acids, 632 were totally conserved, and a further 114 sites were highly conserved (i.e., fewer than 4/67 species have a different amino acid; for 69 of these sites, only 1 species had a different amino acid). These changes occurred in so few species that no conclusions could be drawn about the driver for these changes. For 52 positions, the consensus amino acid was present in less than 90% of the sequences, thus these positions with more frequent alternate amino acids are of greater interest (highlighted in Fig 3 by crossing the dotted line).

The sequence variations are scattered throughout the motor both within and outside the major functional regions of the motor domain with no identifiable pattern to the location of the changes (Fig 3). High levels of variation are found in the N-terminal domain [1–59] and near the surface loops, Loop 1 (near residue 210) and Loop 2 (near 630). These loops are known to be hypervariable across the larger myosin family.

The 52 common sites of variation between species were of interest to compare with amino acids in human β-myosin implicated in myopathies (hypertrophic cardiomyopathies [HCMs], dilated cardiomyopathies [DCMs], and other cardiomyopathies as orange, magenta, and green dots in Fig 3 [14]). More than half the sites in the motor domain (approximately 500/800) have been found to have mutations linked to myopathies, but only 20 out of the 52 sites identified to differ among mammals are common between the 2 groups. Of these, only 1 E44D shares the same mutation; in all other cases, the substituted amino acid is different. Thus, there is no simple correlation between the 2 groups of positions with changed sequences. This is illustrated by the converter domain (710 to 778, region k in Fig 3), which is enriched in myopathy mutations (59 reported mutations at 35 sites in the 68 residues of the converter domain), but there are very few sequence changes between species in this region with only 1 position that differs in more than 2 species (Gly747, replaced by Ser in 7 species).

Further, analysis of gnomAD [15] revealed that there is very little variation in β-myosin within healthy human populations (Fig 3), thus suggesting that the sequence is highly constrained within human but varies between species. This constraint in humans for the MYH7 gene has been analysed in several papers and in databases [16].

#### Distinguishing between variation due to clade and body mass in β-myosin

Change in sequence can be driven by several things. Here, we identified positions of variation that are associated with body mass and also compared this to variation that is clade specific between the 2 main clades present in the data set (Euarchontoglires and Laurasiatheria). Of the 67 complete sequences examined, about half were Euarchontoglires (32, e.g., rodents and primates) with a mass range of 0.011 to 140 kg, and half were Laurasiatheria (30, e.g., bats, ungulates, and cetaceans) with a mass range of 0.0069 to 42,500 kg.

**Fig 4:**
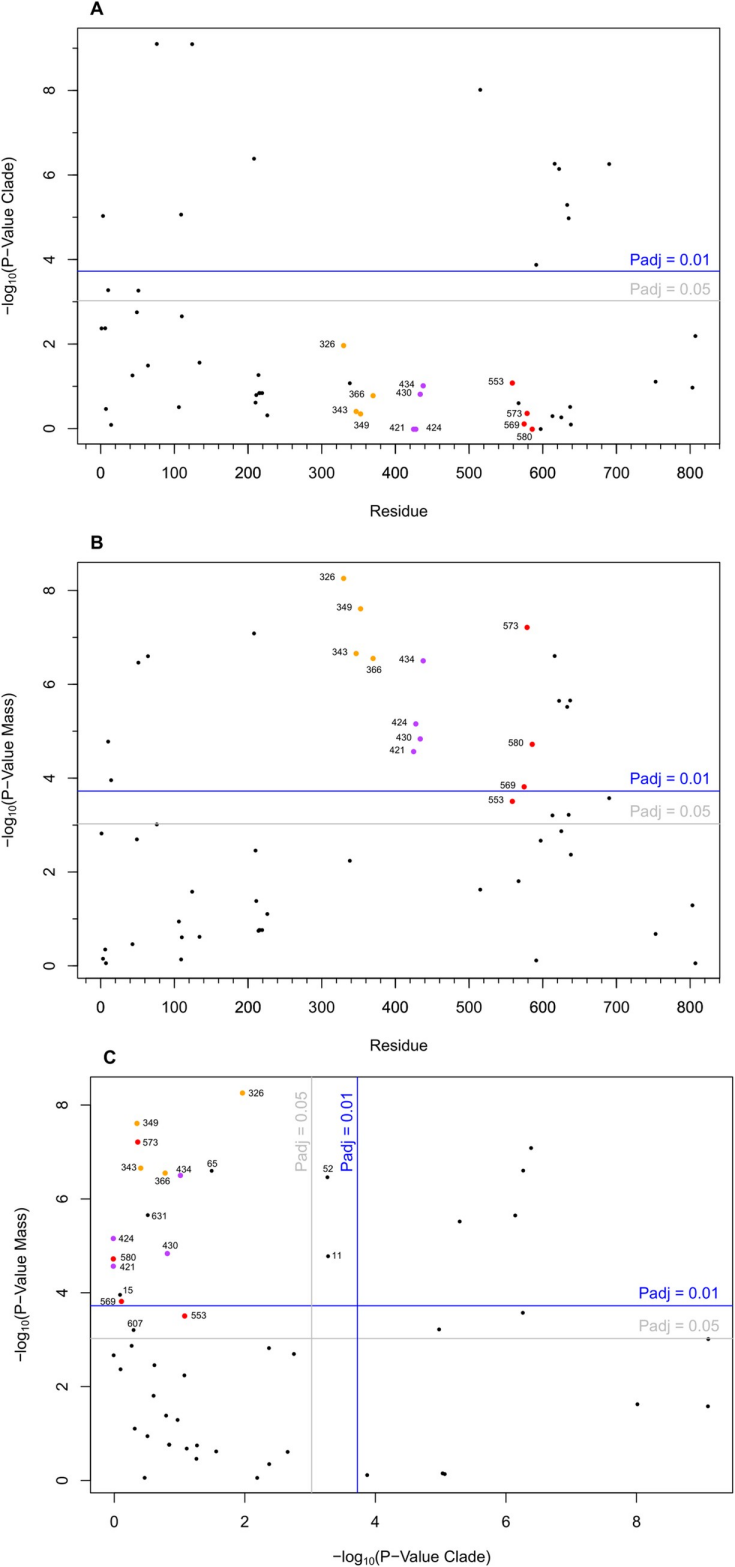
Residue mass change association with mass, clade, and each other. The association of each residue with clade **(A)**, mass **(B)**, and the association of the *p*-values of clade vs. mass **(C)** are plotted. Moreover, 1% and 5% significance thresholds are shown with the Bonferroni adjusted lines (blue and grey). The 3 groups of residues investigated are highlighted in orange, purple, and red and labelled with the human residue numbering in each plot. Raw data files are available at Figshare.

The 52 sites of variation that occur in >10% of species were analysed to distinguish between changes that correlated with clade and those that correlated with body mass as illustrated in Fig 2 for 4 sites with the remaining plots in S2 Fig. At most positions (41/52), only 2 residues were observed at each specific site; in the small number of positions (11/52) with multiple amino acids, only the 2 most frequent residues were considered. The identity of the 2 most frequent amino acids were coded as 0 and 1 (with the amino acid more frequently present in smaller species as 0), and a logistic regression model was fitted with log(mass) as the explanatory variable (Fig 2, S2 Fig; see Materials and methods) to model the transition between residues. For the 4 positions present in Fig 2, 2 had a strong correlation with species body mass (the amino acid common in small mammals is given first) A326P and I349M; (p_adj_ << 0.01. Note the adjusted 1% significance threshold is *p* = 1.92 × 10^−4^ and the 5% significance threshold is *p* = 9.62 × 10^−4^. p_adj_ will be used to indicate the adjusted significance threshold), and each has a distinct midpoint mass for the transition between the 2 amino acids. In contrast, I125V has a low association with mass (p_adj_ > 0.05), and M77L has an intermediate association (p_adj_ approximately 0.05); however, both M77L and V125I have a strong association with the clade (Fig 4). This is highlighted in Fig 2A where the 4 sequence positions are mapped against the species tree; L77 and I125 are found almost exclusively in Laurasiatheria. In contrast, it is clear from Fig 2A that the variation at positions 326 and 349 is not explained by the phylogenetic relationships, with the 2 different amino acids spread across the 2 clades.

Overall, only 12 sites had a very strong association with clade (p_adj_ ≤ 0.01), and a further 2 were significant at the 5% level (p_adj_ ≤ 0.05; Fig 4A). Some of these residues occur in 2 groups; 1 group in the N-terminal region (4, 11, 52, 77, 110, and 125) and 4 residues near surface loop 2 (610, 616, 627, and 629). The remaining 4 are at D208E, E509T, T585I, and I684M. A total of 20 positions were strongly associated (p_adj_ ≤ 0.01) with body mass, and a further 4 were associated at p_adj_ ≤ 0.05 (Fig 4B). Moreover, 9 positions were associated with both clade and body mass (Fig 4C), which is likely to represent that the very largest mammals (body mass > 500 kg) in the data set are all Laurasiatheria (S5 Fig).

**Fig 5:**
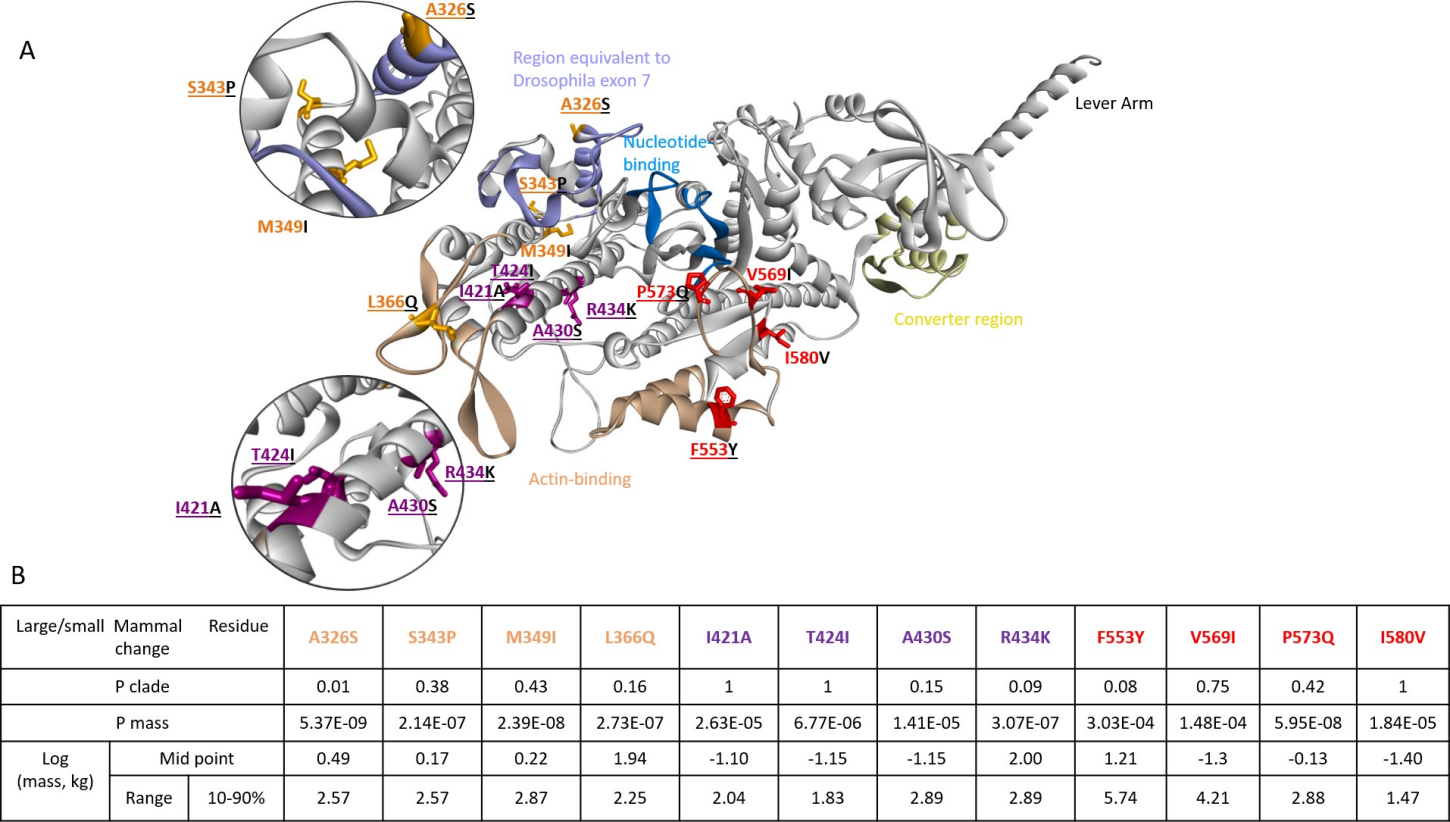
Location of residues switched in the chimera. Structure of human β-myosin (homology model based on Protein Databank ID 6FSA). The actin-binding site is highlighted in brown, exon 7 in blue, the nucleotide binding site in marine blue, and the converter region in yellow. The 3 groups of residues investigated are highlighted and labelled in orange (326, 343, 349, and 366), purple (421, 424, 430, and 434), and red (553, 569, 573, and 580) in each plot, and those that were switched are underlined. The structure shown represents one of the known conformations adopted by myosin during the turnover of ATP and is shown to illustrate the relative position of the residues of interest in relation to, e.g., actin- and ADP-binding sites. Since the residues highlighted are not directly coupled to ADP binding, it is likely that the stability of specific conformations and/or the transition (activation barrier) between conformations are affected by the sequence changes. For a review of the myosin motor domain structure, see [17,18], and for a broader discussion of activation barriers and conformational changes, see S1 Text and [19]. The table indicates the details about the 3 groups of variable residues, the adjusted probability (p_adj_) of association with clade or mass, the log(mass) at the midpoint of the transition of the regression line between the 2 amino acids, and the range (10%–90%) over which the transition occurs.

A total of 12 of the 24 sites associated with body mass occur in the known hypervariable regions, 4 in the N-terminal region (**11**, 15, **52**, and 65, bold **residues** also occur in the clade list), 1 in loop 1 (**D208E**), and a further 6 occur in or near loop 2 (607, **610**, **616**, **627**, **629**, and 631)) and 1 at **I684M**. The remaining 12 sites group into 3 sets of 4, most with p_adj_ ≤ 0.01 (coloured in Figs 4 and 5). Comparing the strength of association between clade and body mass, these 12 sites are strongly associated with body mass but not with clade (Fig 4C). Hence, we propose that these 12 positions are likely to be important in determining the β-myosin velocity of contraction. At 8 of the 12 positions, only 2 amino acids are observed, one position contains 3 amino acids, although the third is only present once (residue 366). Multiple amino acids [4–7] were observed at the remaining 3 positions. For 2 of the positions, 421 and 424, this reflects a subset of the species from one clade having an alternate amino acid in some of the larger species (see S3 Fig).

**Fig 6:**
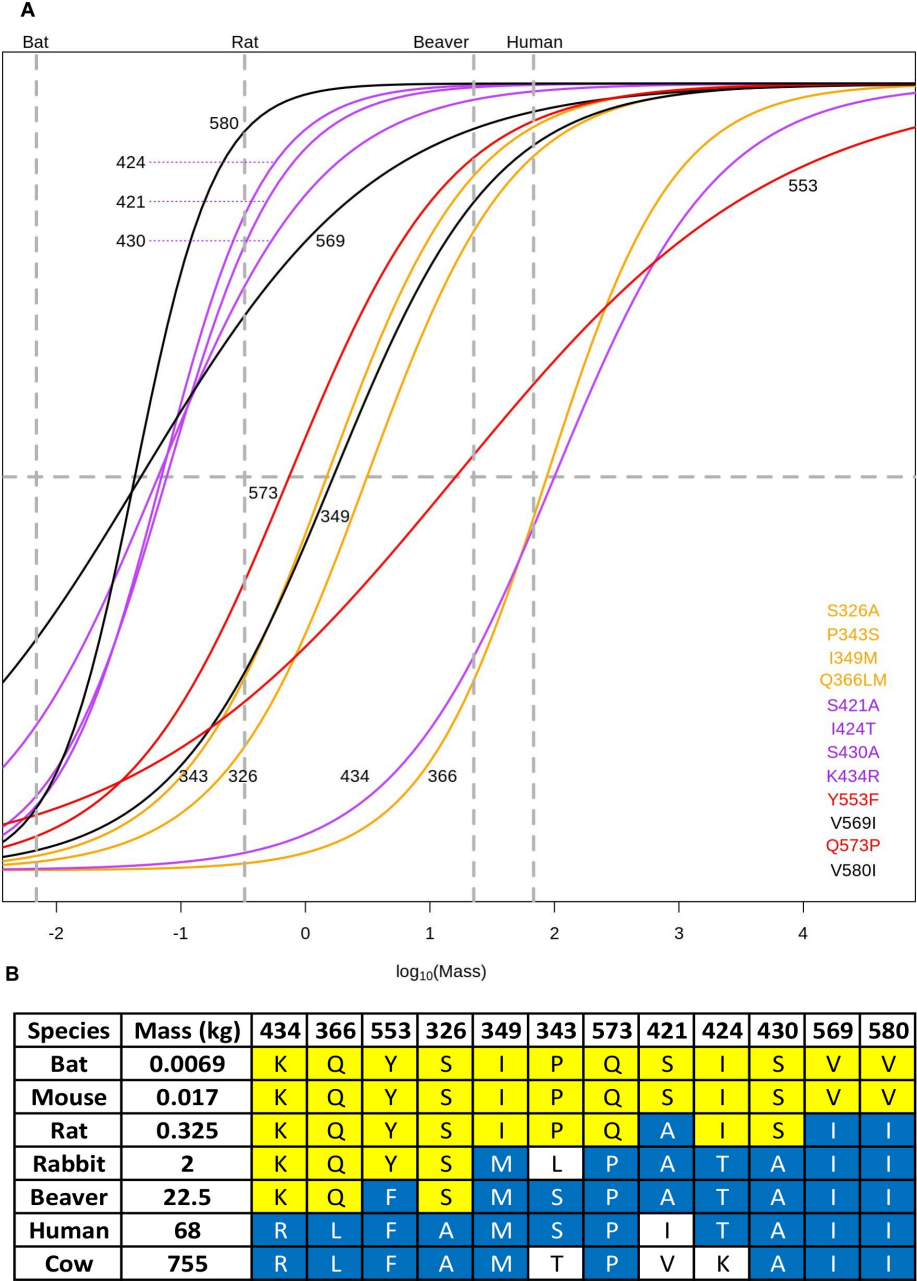
Residue mass transition plots. Overlapping binomial regression mapping the transition of the most frequent amino acid at positions in the motor region of β-myosin to the second most frequent amino acid at that position for the 12 residues of interest. The 3 groups of residues investigated are highlighted and labelled in orange (326, 343, and 366), purple (421, 424, 430, and 434), and red (553 and 573) in each plot. The residues in black are residues of interest, but were not mutated (349, 569, and 580). The table shows the mass and residues for species at different weights, where the bat and cow are from the clade Laurasiatheria, and the other species are from Euarchontoglires clade. Raw data files are available at Figshare.

The first group of residues is in a region 331 to 371 (orange residues in Figs 4–6). One residue, 366, is just before the actin-binding loop known as the cardiomyopathy loop. The remaining 3 residues are adjacent to the region of *Drosophila* myosin II coded by exon 7 (and the “linker region”). This region is one of 4 exons in the single myosin II gene of *Drosophila* which are alternately spliced to generate all isoforms of myosin II in *Drosophila* [20]. We have previously shown [21] that the alternatively spliced forms of this region alter ADP release in the *Drosophila* myosin. The second set (426 to 439; residues magenta in Figs 4–6) is in a long helix (Helix-O) in the upper 50-kDa domain that links the cardiomyopathy loop to the nucleotide binding pocket (via switch 2). The third region (560 to 587; residues red in Figs 4 and 5) is in the lower 50-kDa domain and residue 553 forms part of the helix–loop–helix actin-binding motif, while 573 and 569 are part of actin-binding loop 3. The last residue 580 is on the β-strand that follows loop 3. Thus, 4 of the 12 residues are in or close to actin-binding loops, 3 are close to a region known to influence ADP binding (the region coded by exon 7), and 4 are on the helix that links the actin-binding site to the nucleotide pocket. It is therefore possible that many of these residues could influence how actin binding leads to displacement of ADP.

Comparing the myopathy mutations listed by Parker and Peckham [14], 6 mutations occur in the 16 sites we found to be associated with mass (p_adj_ < 0.05) but not with clade. Each of these myopathy sites have a different substitution to the one found across mammals except R434K. Of the 6 sites, ClinVar [22] lists only A326P as a potential cardiomyopathy mutation. The others are classified as benign or of uncertain significance.

#### Experimental testing of the computational predictions

We have previously expressed the motor domain of human β-myosin in mouse C_2_C_12_ muscle cells and isolated the protein using His tags attached to the co-expressed human light chain. This is currently the only way to express mammalian striated muscle myosin motors domains but is complex and time consuming and yields just a few mg of protein [21,23,24]. To test the hypothesis that the highlighted group of 12 residues is responsible for a significant part of the adjustment of ADP release rate constant, we generated a chimeric human-rat β-myosin motor domain in which the region containing the 9 positions (of the 12) that vary between human and rat was replaced with the amino acid sequence present in rat (A326S, S343P, L366Q, I421A, T424I, A430S, R434K, F553Y, and P573Q—human residue number and amino acid listed first). The other 3 positions are the same in rat and human (349, 569, and 580). At residue 421, we replaced Ile with Ala as present in rat, although Ser is present in most of the smallest mammals (see S5 Fig).

As shown in Table 2, the velocity of contraction for β-cardiac/Type I slow muscle fibres in rat and humans differ by a factor of approximately 4. Given that these residues have a range of transition masses (see Fig 6 and Discussion section), the hypothesis is that each of these 9 residues will contribute a fraction of the difference between the rat and human β-myosin ADP release rate constant, and, hence, velocity. With all 9 residues changed, our prediction was that the differences in the rate constant would be large enough to be easily detectable.

The S1 fragment of human β-myosin and the chimera was expressed in C_2_C_12_ cells and purified with the human essential light chain attached. Few details of the kinetic characterisation of the rat β-myosin S1 have been published [16]. The rat β-myosin S1 was therefore purified from rat soleus muscle to use as a comparator for the chimera. S4 Fig shows the SDS-PAGE of all 3 proteins used in this study and demonstrates that all 3 proteins are pure and contain the appropriate light chains.

**Fig 7:**
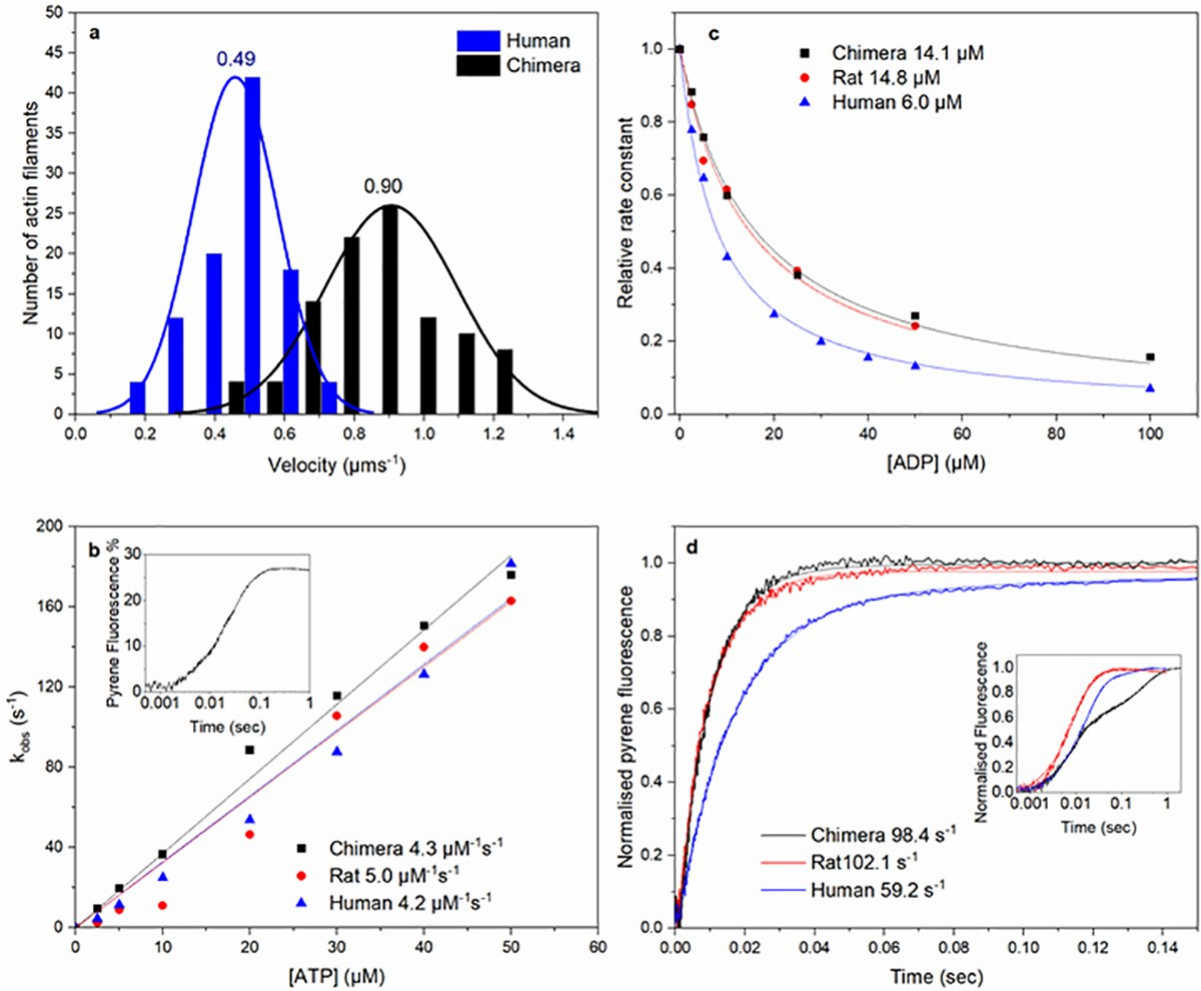
In vitro analysis of the chimera, rat and human β-S1 proteins. **(A)** Histogram of in vitro velocity of 100 rhodamine-labelled phalloidin actin filaments moving over human β-S1 or chimera S1. The solid line shows the data fitted to a single Gaussian curve. The mean velocity for the human β-S1 was 0.49 ± 0.028 μm s^−1^ and for the chimera, 0.90 ± 0.015 μm s^−1^. **(B)** The effect of ATP concentration on k_obs_ for ATP-induced dissociation of pyrene-actin.S1. The gradient generates a second-order rate constant of ATP binding; the values for the 3 proteins are highlighted next to the plot. Inset shows an example transient of 50 nM pyrene actin-chimera S1 mixed with 20 μM ATP, resulting in a fluorescence change of 26%. **(C)** Plot of relative observed rate constant dependence on [ADP] for the ATP-induced dissociation of pyrene-actin.S1. A total of 50 nM pyrene-actin.S1 was mixed with 10 μM ATP and varying [ADP] (0–100 μM). Numbers indicate the values of ADP affinity for acto.S1, K_ADP_, for the 3 proteins. **(D)** To measure k_+ADP_, ADP is displaced from pyrene-actin.S1.ADP complex by an excess of ATP. A total of 2 mM ATP was mixed with 250 nM S1 which was preincubated with 500 nM pyrene-actin and 100 μM ADP. k_+ADP_ values for the 3 proteins are given in 7D. Inset showing data on a longer log timescale showing the slow phase components of the transients. The average values from 3 independent measurements for experiments shown in B, C, and D are summarised in Table 3. Raw data files are available at Figshare. The inset shown in Fig 7D, a complication of the ADP displacement measurement, is that ADP displacement from human β-myosin occurs in 2 phases (fast and slow). The fast phase corresponds with ADP released at the end of the normal ATPase cycle, while the slow phase is a trapped ADP which is released much slower and at a much slower rate than the overall cycling. This is therefore a dead-end side branch of the pathway commonly seen in slow muscle and non muscle myosins [11,24,25]. The fraction of ADP trapped in this way is characteristic of each myosin. The rat β-myosin S1 has no apparent slow phase, the human has approximately 10% of ADP released in the slow phase, while the chimera has a larger fraction (approximately 40%) of the total ADP released in the slow phase. The role of the substituted amino acids in the slow phase requires further study, but the reader is referred to the literature for a broader study of this phenomena.

As a test of the functions of the chimeric protein, the ATP-induced dissociation of the chimera from pyrene labelled actin was monitored and compared to the recombinant human and the native rat S1. A typical transient is presented (inset in Fig 7B), and the observed amplitude of the signal change was the same for all 3 proteins. The similarity of observed amplitudes of the pyrene signal changes for the chimera, human, and native rat proteins indicates that the chimera binds actin and releases it on ATP binding as for the human and rat S1. This is consistent with the chimera being a fully folded and active protein. A plot of the observed rate constant (k_obs_) versus [ATP] gives a straight line which defines the apparent second-order rate constant for the reaction (Fig 7B, Table 3) and appears the same for all 3 proteins. The observed rate constant of this reaction has been defined for many myosins and has 2 components, k_obs_ = [ATP] K’_1_k’_+2_. The reaction is sensitive to both the affinity of ATP for the complex (K’_1_) and the efficiency with which ATP induced a major conformational change in the myosin (k’_+2_). This involves the closure of switches 1 and 2 onto the ATP and the opening of the major cleft in the actin-binding site of myosin. The absence of any change in K’_1_k’_+2_ is consistent with a well-preserved nucleotide pocket and a preserved communication pathway between the ATP binding pocket and the actin-binding site.

**Table 3:** Comparison of ATP and ADP binding parameters of native rat S1 and the C_2_C_12_ cell–expressed human β-myosin and chimera S1.

	Rat native S1	Chimera S1	Human S1	Chimera/rat ratio	Chimera/human ratio
**ATP binding to A.S1 (μM**^**−1**^**s**^**−1**^**)**	5 ± 0.16	4.5 ± 0.2	4.4 ± 0.3	0.9	1.02
**ADP affinity to A.S1 (μM)**	14 ± 0.09	14 ± 0.14	6.1 ± 0.7	1	2.3
**ADP release from A.S1.ADP (s**^**−1**^**)**	107.2 ± 7.3	100.7 ± 4.2	59 ± 3.3	0.94	1.71
**Motility (μm.s**^**−1**^**)**	NA	0.90 ± 0.221	0.49 ± 0.129	NA	1.84

Errors reported are SEM, except for motility, which is the HWHM of the normal distribution.

Data are from 3 separate preparation of protein from either different cell pellets (expressed protein) or different soleus muscles from the rat. Experimental conditions were 25 mM KCl, 20°C.

HWHM, half width at half maximum; SEM, standard error of the mean.

The affinity of ADP for actin.S1 was measured in a competition assay with ATP (Fig 7C), and the affinity of ADP for the rat actin.S1 complex (14 μM) was 2.3-fold weaker than for the human wild-type (WT) protein (6.3 μM). These values are consistent with published values [26]. The chimera was distinct from the human S1 and indistinguishable from the rat S1. To confirm this result, the ADP release rate constant was measured directly by displacing ADP from actin.S1.ADP through addition of an excess of ATP. The results (Fig 7D) for human and rat S1 are again consistent with published values with ADP, leaving the rat complex at approximately twice the rate of the human complex (107 versus 59 s^−1^). The chimera was indistinguishable from the rat S1. As predicted, the amino acids introduced into the human β-myosin motor domain weaken ADP affinity for actin–myosin by accelerating ADP release to make the human β-myosin S1 behave like the rat β-myosin S1.

Motor activity of the recombinant human β-myosin S1 and the chimera protein was measured using an in vitro motility assay (Fig 7A, S1 Video). This assay determines the myosin-mediated velocities of fluorescent actin filaments moving on a nitrocellulose-coated slide surface. The human WT β-myosin moved actin at a velocity of 0.49 μm.s^−1^, at 20°C. For the chimera, the mean filament velocity was almost 2-fold faster than human WT at 0.9 μm.s^−1^, which is consistent with the approximately 2-fold increase in ADP release-rate data. Our human S1 velocity was similar to the 0.612 μm.s^−1^ value reported by Ujfalusi and colleagues, which was measured at 23°C [27]. A similar velocity of 0.378 μm.s^−1^ was reported for full length human-β myosin at 25°C and a velocity of 0.624 μm.s^−1^ for the rat. This gives a rat/human ratio of 1.65, very similar to our chimera/human ratio (1.84). The motility assay was not performed for the rat S1 as we do not have an expression system for the protein. The native rat S1 has only a single light chain and lacks a tag to attach the protein to the surface. The rat protein will not therefore give a valid comparable measurement. However, it is known from the literature (Table 2 and references therein) that the rat protein moves 3 to 5 times faster than the human protein, depending upon the exact measurement conditions.

## Discussion

The well-established observation that the muscles of large mammals contract at slower speeds than small mammals [28] and that the maximum contraction velocity is a property of the myosin isoforms expressed in the muscle [1] led us to ask if it is possible to define which amino acid changes are responsible for the change in contraction velocity. Our analysis of a large number of myosin II sequences indicates that adult sarcomeric myosins have a larger variation in sequence than 2 of the non-muscle myosins (or developmental striated muscle myosin isoforms), which are expected to be independent of species size. Furthermore, there appears to be a correlation between size difference and sequence difference.

Examination of 67 mammalian β-myosin sequences identified sequence changes at 12 sites, which had a high correlation with species size and little correlation with clade. Fig 6 plots the probability of transition versus species mass for each of these sites and indicates that there is a distinct midpoint of transition for each site as shown in Fig 5B. Reading the mass of a given species on the x-axis allows the probability of each site having the amino acid associated with small or large species to be estimated. The trend in midpoint values implies that there is a distinct order in which the amino acids at the 12 sites change as the species mass increases from the smallest to the largest. Furthermore, the order in which the sequence changes is similar for both clades. This is illustrated for 7 species in Fig 6B where the 7 sites are shown in the order of the midpoint mass of the transition. The sites change from yellow to blue as the species mass increases in the order expected. A similar plot for all 67 species is given in S5 Fig and shows the same phenomena but, as might be expected, with more scatter in the data.

Our analysis suggests that there are reasons to believe that the sequence of the motor domain is changing with the mass of the species. Before considering the sequence changes in the motor domain of β-myosin in more detail, it is useful to consider why contraction velocity and specifically the maximum velocity is an important factor in defining muscle performance.

### The central role of V_0_ in muscle physiology

The maximum contraction velocity of a muscle, V_0_, is a key attribute of muscle contraction that plays a significant role in defining both the force–velocity relationship, power output, and the efficiency of muscle contraction [4, 5]. V_0,_ one part of the force–velocity relationship of a muscle, as empirically defined by Hill in 1938, is a fundamental property which underpins both the power output (power = force × velocity) and the contraction velocity at which power and efficiency of contraction are maximum, normally considered the optimal working conditions for a muscle. Power and efficiency are fundamental parameters that define the movement of an animal and crucially the output of the heart. In contrast to V_0_, which varies more than 10-fold for different myosin isoforms [29], the maximum force a myosin can generate varies relatively little between myosin isoforms when assessed at the single molecule level or in the muscle sarcomere [5,29]. Maximum velocity is therefore a central parameter that plays a significant role in defining both the force–velocity relationship, power output, and the efficiency of muscle contraction.

There are strong theoretical arguments and experimental evidence that ADP release is the event in the crossbridge cycle that defines the maximum velocity of contraction in β-myosin (Table 2). We need to understand how the rate of ADP release is adjusted to match the physiological requirements.

### How does the location of the 12 residues in the motor domain influence ADP release, and, hence, V_0_?

The sites identified are not directly linked to the ADP binding pocket, and this is not a surprise since the same pocket binds ATP and compromising ATP binding is likely to be detrimental to myosin function. This illustrates the problem in modifying myosin; the mutation should affect only ADP release and not any other event in the cycle, which, it is assumed, are optimised to make efficient use of the energy of ATP hydrolysis.

Some loops on myosin’s surface are known to be variable across myosin in general, and surface loop 1 has been shown to modulate ADP release in some myosins (scallop muscle myosin II and smooth muscle myosin II have an alternate splice in this region [29,30], and smooth muscle myosin, Dictyostelium myosin II, and human myosin 1b have been modified to explore the role of variations in this region [31–33]). Loop 1 does show some variations here, but this has no correlation with size. Note that the rat and cow share identical sequences in this region.

Myosin is a coordinated mechanical system with each part of the structure able to sense allosteric changes across the structure as a whole—as illustrated by the many mutations in β-myosin that have been explored. Each mutation can have multiple effects on the cycle as a whole. Thus, in principle, mutations anywhere in the motor domain could influence ADP release, but the system is tightly constrained because ADP release must be modulated without a negative effect elsewhere in the cycle. There will be a limited number of ways in which this can be achieved. The evidence from Fig 6 is that a solution to this problem exists, and both clades have found the same solution.

*Drosophila* has multiple isoforms of muscle myosin II (flight, cardiac, leg, embryonic muscle, etc.), and all of these isoforms are encoded by a single gene. Alternate splicing of 4 regions of the motor domain results in the expression of each different isoform. One of these regions is coded by exon 7, which has been shown to modulate ADP release, and this region overlaps with some of the mutations highlighted here. Thus, different routes to modulating ADP release are possible, and it is intriguing that *Drosophila* and mammals use the same region. To define exactly how the set of 12 sites identified here can modulate, ADP release and V_0_ will require more detailed molecular structures and detailed molecular dynamics.

One question about the relationship between myosin sequence and mammal size is whether such a correlation is seen with any other sarcomeric protein. It is of course impossible to be definitive about this, but examination of the other major sarcomeric proteins actin, tropomyosin, and the 3 troponin components (I, T, and C) reveals no such correlation. In fact, actin and tropomyosin are among the most conserved eukaryotic proteins showing little variation among mammals or indeed vertebrates [34,35]. Troponins do have a higher degree of variation than actin or tropomyosin, but we found no evidence of a correlation between sequence and mammal size. There has been a report of a correlation in the size of repetitive Pro-Ala rich regions in myosin binding protein C [36] and in myosin light chain 1 [37], but these have, to date, only considered a small number of species and include a broader data set of vertebrates.

In conclusion, we have used a computational approach to identify variation in β-myosin that is associated with increasing body mass, which would have a role in reducing heart rate. The experimental characterisation of the chimeric human-rat β-myosin demonstrates that these residues do indeed control the rate of ADP, the rate limiting step in the myosin cycle, and thus are likely to have adapted to enable the slower heart rate required in larger animals as they have evolved from small to large (Copes Law; [38,39]).

## Materials and methods

Protein sequences were extracted from RefSeq [40] and UniProt [41] as listed in S2 Table. To ensure that each sequence corresponded to the specific myosin isoform, we used both the UniProt and eggNOG [42] annotations and for the model species available in APPRIS [43] selected the principal isoform. Isoform determination was also checked with the gene tree (S6 Fig) of all sequences. There were 15 sequences in which eggNOG gave a different assignment to UniProt (6 NMA, 7 α, and 1 IIa); these are described in S1 Text.

Incomplete sequences were excluded from our analysis because sequence gaps could have a major effect on the sequence comparison for such closely related isoforms.

For each isoform, the protein sequences were divided into the Motor (1–800, β-myosin numbering) and Tail (842 to 1,936) regions. For each region of each myosin, the sequences were aligned using Clustal Omega [35]; the resulting multiple sequence alignment was used to construct a percentage identity matrix between the species. Sequence identity was used rather than sequence similarity as we are considering small changes (>93% identity and >98% similarity) within the isoform and substitutions that would normally be classed as similar (e.g., aspartate to glutamate) may be relevant.

The masses of each species were extracted from a wide range of information sources and are listed in S2 Table. Where the literature provides a range of adult body masses, the arithmetic mean was selected. To compare sequence divergence against either evolutionary time or animal body mass, the relevant matrices were plotted against each other. Amino acid sequences for the β-myosin motor from 67 different mammalian species comprised organisms from the clades Euarchontoglires [31], Laurasiatheria [29], Metatheria [4], and Afrotheria [1].

Protein sequence divergence was plotted against the species mass. A robust regression was fitted to reduce the weighting of the outliers. This was done by minimising absolute difference rather than squared distance, which should reduce the amount of under- and overestimation in value difference caused by the square.

Residues associated with cardiomyopathies were obtained from Parker and Peckham [14] and compared to the residues associated with body mass by considering exact matches to residues associated with cardiomyopathy. These were grouped into HCM, DCM, and other.

### Phylogenetic independent contrasts

Two statistical methods were used to create phylogenetic trees, maximum likelihood (ML), and Bayesian inference methods, using programmes Randomised Axelerated Maximum Likelihood (RAxML) [44] and Bayesian Evolutionary Analysis Sampling Trees (BEAST) and its corresponding user interface BEAUtI [45], to increase reliability as species sequence identity is high. The RaxML trees were generated using the CIPRES Science Gateway [46]. TreeAnnotator [45] was then used to generate a consensus tree for each set of 10,000 trees produced by BEAST. *Monodelphis domestica* (opossum) was selected as an outgroup. The ML and Bayesian trees were compared using Compare2Trees [47], with black nodes on the trees indicating branches where the 2 trees disagree. Trees were drawn using FigTree [48].

PICs [8] were performed using the ape [49] and phytools [50] packages in R. This analysis used species trees generated from TimeTree from the species present in our analyses for the β, α, IIa, IIb, and IIx isoforms.

### Statistical analysis

For each position in the alignment that had more than 1 amino acid present, species masses were compared between the 2 highest frequency amino acids at that position using the Mann–Whitney U test, a nonparametric 2-sample test. Multiple testing was accounted for by applying the Bonferroni correction. To avoid very imbalanced comparisons, the analysis was not run if the frequency of the second amino acid was less than 10% of the frequency of the most frequent one. Where more than 2 amino acids were present at an alignment position, only the 2 most frequent amino acids were considered. See S3 Fig for details of sites with more than 2 amino acids.

Alignment positions were divided into 3 groups: those with an adjusted *p*-value (p_adj_) less than 0.01 (with Bonferroni correction applied this is equivalent to a *p*-value of *p* = 9.50x10^-04^), those with 0.01 < p_adj_ < 0.05 (5% significance threshold equivalent to *p* = 9.6x10^-4^), and those with a p_adj_ >0.05. In addition, the 2 highest frequency amino acids were coded as 0 and 1, and a logistic regression model was fitted with log(mass) as the explanatory variable (Fig 3, S2 Fig). In order to overlay these residue plots, as the coding of the amino acids as 0 and 1 was arbitrary, it was done in such a way that the slope of the fitted logistic regression line was positive (Fig 3). The value of mass at which the 2 amino acids were predicted to be equally likely to occur was estimated from the regression line.

For each alignment position, a 2 × 2 table was constructed, classifying the species by amino acids present (most frequent and second most frequent) and clade. Fisher exact test was used to test whether these 2 factors were associated. The residue and −log_10_ of the *p*-value from the Fisher exact test were plotted to identify residues for which the amino acid variation was likely to have resulted from clade-associated changes. The residue and −log_10_ of the *p*-value from the Mann–Whitney U test were also plotted to determine when residue variation was likely attributed to mass changes. Finally, the −log_10_ *p*-values obtained from both tests were plotted against each other. For each of these plots, lines at positions of the Bonferroni adjusted *p*-values 0.01 and 0.05 were added to assess the confidence in each residues association with mass or clade.

All statistical analyses were run in R [51].

### Molecular biology of the chimera

A pUC19 plasmid containing the human β-myosin motor domain gene was digested with NsiI and NgoMIV to excise DNA encoding for residues 310 to 599 of the human β-myosin. This region was replaced with a complementary pair of synthetic oligos encoding for the same region, but with the 9 amino acid substitutions listed (Ala326Ser, Ser343Pro, Leu366Gln, Ile421Ala, Thr424Ile, Ala430Ser, Arg434Lys, Phe553Tyr, and Pro573Gln). The subsequent clone was confirmed by sequencing. This chimera gene was cloned into a pShuttle CMV vector to allow recombinant replication-deficient adenovirus production, as previously described [10].

### Protein purification

The chimera and the human β-myosin motor domains (known as subfragment 1 or S1) were expressed and purified as descried previously [25]. Briefly, the adenoviruses were used to infect C_2_C_12_ myotubes in culture and resulted in overexpression of recombinant myosin proteins. The heavy chains (residues 1–842) were co-expressed in C_2_C_12_ myotubes with His-tagged human ventricular essential light chain. The recombinant proteins also carried the endogenous mouse regulatory light chain (MLC3). This is homologous to subfragment 1, S1, generated by proteolytic digestion of myosin. For motility assays, the heavy chain was additionally tagged with an 8 residue (RGSIDTWV) carboxyl-terminal extension. Cell pellets were homogenised in a low salt buffer and centrifuged, and the supernatants were purified by affinity chromatography using a HisTrap HP 1 ml column. The proteins were then dialysed into the low salt experimental buffer (25 mM KCl, 20 mM MOPS, 5 mM MgCl_2_, 1mM DTT, pH 7.0).

The SNAP-PDZ18 affinity tag used for in vitro motility measurements were purified as described in [52,53]. SNAP-PDZ18 was expressed through a pHFT2 expression vector, and the plasmid transformed into *Escherichia coli* BL21 DE3 cells. The protein was purified using nickel affinity chromatography and dialysed in PBS.

Actin was prepared from rabbit muscle as described by [54]. The actin was labelled with pyrene at Cys-374 as described in [55]. When used at sub-micromolar concentrations, the actin was stabilised by incubation in a 1:1 mixture with phalloidin.

Rat β-myosin S1 was prepared from soleus muscle which was dissected immediately postmortem and stored on ice. The muscle was homogenised into Guba-Straub buffer and left to stir for 30 minutes. After centrifugation at 4,600 Revolutions per Minute (RPM) for 30 minutes at 4°C, the supernatant was subject to myosin precipitation as described in [56]. The resulting myosin was digested with 0.1-mg chymotrypsin per ml of solution and left to stir for 10 minutes exactly, at room temperature. To stop the digestion, 0.5 mM phenylmethylsulfonyl fluoride (PMSF) was added and the solution left to stir for 10 minutes. The digested myosin solution was dialysed into the low salt experimental buffer overnight (25 mM KCl, 20 mM MOPS, 5 mM MgCl_2_, 1mM DTT, pH 7.0). Precipitated myosin and light meromyosin was pelleted and removed via centrifugation at 12,000 RPM for 10 minutes, with the supernatant containing the purified soleus S1. SDS gels of the purified protein were run and compared to the expressed human β-myosin and chimera S1.

### Stopped-flow spectroscopy

Kinetic measurements for S1 of chimera, human β-myosin, and rat soleus myosin were performed as described previously [25,57,58]. Solutions were buffered with 25 mM KCl, 20 mM MOPS, 5 mM MgCl_2_, 1 mM DTT at pH 7.0, and measurements were conducted at 20°C on a High-Tech Scientific SF-61 DX2 stopped-flow system. Traces were analysed in Kinetic Studio (TgK Scientific, Bradford-on-Avon, UK) and Origin.

### In vitro motility assay

Motility assays were performed essentially as described previously [53,59]. Briefly, flow chambers were constructed with coverslips coated with nitrocellulose mounted on glass slides. Reagents were loaded in the following order: (1) SNAP-PDZ18 affinity tag; (2) BSA to block the surface from nonspecific binding; (3) S1 of human β-myosin or the chimera with an 8-amino acid carboxyl-terminal affinity clamp; (4) BSA to wash the chamber; (5) rhodamine–phalloidin-labelled rabbit actin; (6) an oxygen-scavenging system consisting of 5 mg/ml glucose, 0.1 mg/ml glucose oxidase and 0.02 mg/ml catalase 7; 2 mM ATP. Partially inactivated myosin heads in S1 preparations were removed by incubating with a 10-fold molar excess of actin and 2 mM ATP for 15 minutes, then sedimentation at 100,000 RPM for 15 minutes. Supernatant was collected containing active myosin heads. All solutions were diluted into 25 mM imidazole, 25 mM KCl, 4 mM MgCl_2_, 1 mM EGTA, 1 mM DTT, pH 7.5. Actin filaments were detected using a widefield fluorescence imaging system (described in [60]) with UAPON 100XOTIRF NA lens (Olympus, Hamburg, Germany) and QuantEM emCCD camera (Photometrics, Arizona, USA). The system was controlled and data analysed using Metamorph software (Molecular Devices, Sunnyvale, USA). Assays were performed at 20°C and were repeated with 3 fresh protein preparations, with at least 3 movies of 30 second duration, recorded at a rate of 0.46 seconds per frame. Individual velocities were determined from motile filaments that demonstrated a smooth consistent movement over 10 frames (4.6 seconds). A total of 100 individual measured velocities were used to calculate the mean velocity for each recombinant myosin.

## Supporting information

S1 Text Results discussing the assignment of isoforms to myosin sequences and why conservative amino acid changes are potentially important in tuning muscle contraction velocity. (PDF)

S1 Fig Mass vs. sequence identity plots. The motor and tail domains have been analysed separately. The grey squares are Euarchontoglires, the black triangles are Laurasiatheria, and the open circles are the Afrotheria and Metatheria groups. Each plot has been fitted with a robust linear regression. Sequence identity is pairwise to the mouse. The R^2^ value and slope gradient are shown on each plot. Raw data files are available at Figshare.
(PDF)

S2 Fig Residue mass transition plots. Binomial regression mapping the transition of the most frequent amino acid at positions in the motor region of β-myosin to the second most frequent amino acid at that position. The residue numbering is that of the human β-myosin, as oppose to the alignment position. The black squares are Euarchontoglires, and the triangles are Laurasiatheria. The *p*-value with each plot indicate the probability that the transition of the amino acids is not a result of change in mass. AliPos refers to positions in the sequence alignment that are not present in human β-myosin. Raw data files are available at Figshare.
(PDF)

S3 Fig Highly variable residue mass vs. amino acid frequencies. Residues which had more than 2 sites of variation, with the third most frequent amino acid being close in frequency to the second most common amino acid. The black squares are Euarchontoglires, and the triangles are Laurasiatheria. The residue numbering is that of human β-cardiac myosin. Raw data files are available at Figshare.
(PDF)

S4 Fig SDS-PAGE of the 3 protein preparations. **(A)** Recombinant human β-S1 with 2 light chains. **(B)** Native rat β-S1 and recombinant chimera S1 with 2 light chains.
(PDF)

S5 Fig Distribution of amino acids at 12 positions. This table shows the amino acids that occurs in each of the 12 residues, those predicted to be assocaited with mass and that are of interest, in each species, sorted by mass. Yellow background is the predominant amino acid in small mammals. Blue background is the predominant amino acid in large mammals. The clades Laurasiatheria, Euarchontoglires, Metatheria, and Afrotheria are represented by the letter L, E, M, and A, respectively.
(PDF)

S6 Fig Tree of all myosin isoforms. ML phylogenetic tree generated for all myosin isoforms. Isoforms are labelled according to the key. Zoomed in sections show the α and β regions, and the NMA and NMB regions. Bootstrap values for the isoform branches are shown adjacent to the bars identifying each isoform. ML, maximum likelihood.
(PDF)

S7 Fig Phylogenetic (gene) trees for sarcomeric isoforms. Phylogenetic trees for IIa, IIb, IIx, α, and β isoforms motor and tail domains.
(PDF)

S8 Fig Phylogenetic (species) trees for sarcomeric isoforms. Phylogenetic trees for IIa, IIb, IIx, α, and β species generated from TimeTree.
(PDF)

S1 Table Mass bootstrapping for each myosin isoform. The mass of species for each isoform was randomised 1,000 times at 0.8–1.2 times the mass value for each given species. This table shows the range of values produced from this analysis. The values for the motors are shown in A and values for the tails in B.
(PDF)

S2 Table Accession numbers for isoforms considered. (XLS)

S1 Video In vitro motility of WT and chimeric beta-myosin. WT, wild-type.
(MOV)

## Abbreviations

BEAST: Bayesian Evolutionary Analysis Sampling Trees
DCM: dilated cardiomyopathy
HCM: hypertrophic cardiomyopathy
ML: maximum likelihood
NMA: non-muscle A
NMB: non-muscle B
NMC: non-muscle C
PIC: phylogenetically independent contrast
PMSF: phenylmethylsulfonyl fluoride
RAxML: Randomised Axelerated Maximum Likelihood
RPM: Revolutions per Minute
WT: wild-type
