# Supplementary material for: Identification of sequence changes in myosin II that adjust muscle contraction velocity
Source: PLoS Biol. 2021 Jun 10;19(6):e3001248. doi: 10.1371/journal.pbio.3001248 (PMC8191873; doi:10.1371/journal.pbio.3001248)
Supplement: S1 Text — (PDF) [file pbio.3001248.s001.pdf]

Supplementary Results

Assignment of isoforms to myosin sequences

Our assignments of the isoforms is based on the data in UNIPROT. We have checked all of the sequences against several data bases including NCBI, and eggnog. All agree on the isoforms except eggnog in which 15 of the sequences had been assigned to a different isoform. In eggnog six NMB sequences have been assigned to NMA but this gives these species two copies of NMA and no NMB sequence. Similarly, seven α sequences have been assigned to β with the same results – two β sequences and no α. One IIa sequence was assigned to IIb. Given these anomalies we have used the UNIPROT assignments. Furthermore, we have generated a phylogenetic tree (Supplementary figure S6) in which sequences for each isoform group into the expected isoform.

Why are conservative amino acid changes potentially important in tuning muscle contraction velocity?

The velocity of contraction is 2-3 fold faster for a rat muscle expressing β-myosin than for the same isoform in human muscle. Before considering the role of conservative changes in sequence it is constructive to examine the free energy changes involved in such an acceleration of velocity.

The relationship between the rate constant defining a chemical/biochemical reaction and temperature was defined empirically by Arrhenius as $k = A\ e^{-Ea/R.T}$ where R is the gas constant, T is absolute temperature, A is an arbitrary constant and Ea is the activation energy – the energy barrier between the reactants and products. Essentially the ratio of the activation barrier (Ea) to thermal energy (R.T) defines the fraction of molecules with sufficient kinetic energy from the environment (at temperature, T) to pass over the barrier.

ADP release from the actin-myosin complex is the event thought to limit the velocity of contraction (see Table 2 and Fig 6). The activation energy for ADP release from A.M.ADP in human β-myosin has been measured as ~ 90 kJ mol^{-1} [1], while at room temperature (298 K) R.T is 2.48 kJ mol^{-1}. This means that few molecules at any one time will react. The rate of reaction can be accelerated by increasing the temperature or lowering the activation energy. The activation energy is measured from the temperature dependence of the rate constant and an activation energy of 90 kJ mole^{-1} is typical of many protein reactions. For an Ea of this size an increase of temperature of 10 K (or ~3% of 298 K) will increase the rate constant by ~3 fold. Changing the sequence of a protein can increase the rate of a reaction by reducing the activation energy. From the Arrhenius equation it is simple demonstrate that to increase k by a factor of 3 (at constant T and A) requires a decrease in Ea of the order of 2.7 kJ. mol^{-1} . i.e.

$k/k' = 3 = A\ e^{-Ea/RT} / A\ e^{-E'a/RT}$ or $\ln 3 = (E'a - Ea)/R.T$. For a temperature of 298 K and R = 8.314 $J.mol^{-1}\ K^{-1}$, then E'a- Ea = 2.7 $kJ.mol^{-1}$.

The decrease in activation energy is therefore small compared to Ea itself (2.7 vs 90 kJ mol^{-1}), small compared to a weak side chain interaction (e.g., a hydrogen bond is ~ 20 kJ mol-1) and the increase in k is equivalent to that induced by a 10 K temperature rise. In the case of the human-rat chimera we show that a 2-3 fold acceleration in the rate constant of ADP release and the velocity in the motility assay can be induced by a set of 9 amino acid substitutions some conservative (K434R, Y553F) others not (P343S, Q573P). The contribution of each side chain is impossible to assess from our work, we assume each makes a small contribution to the overall change. If we assume, for the sake of argument, that each amino acid substitution makes a similar size contribution to the change in Ea then each would contribute 2.7/9 or 0.30 kJ $mole^{-1}$ the equivalent of a 1 K temperature rise. From this calculation it would be unwise to assume a conservative amino acid substitution cannot make a contribution to the change in Ea. The slow change of contraction velocity over time then occurs by the accumulation or multiple very small changes in sequence each conferring a marginal advantage.

Conserved amino acids are defined as having similar properties (charge, hydrophobicity etc) but they are not identical. Small changes considered conservative Asp/Glu, Lys/Arg or Val/Lue can have a significant effect on activity. For example, the well-known serine protease family has a catalytic triad of Ser/His/Asp which is invariant, the Asp for example cannot in general be replaced by Glu [2]. In the case of the myosin ~ 1000 mutations in human MyHC7 have been reported which are linked to familial cardiomyopathies of these ~60% are in the motor domain (reviewed in Parker & Peckham 2020[3]. These myosins, containing a mutation are for the most part functional but have a hyper- or hypo- contractile phenotype often resulting in disease in adulthood. Although most of the mutations would be considered non-conservative, many are not e.g., Asp/Glu (positions 554, 497), Val/Leu (pos 186, 216), Arg/Lys (pos 207, 721) Leu or Val/Met (338, 427).

In addition to being important in specific interactions in protein catalysis, in ligand recognition and protein stability, amino acid side chains are important in conformational flexibility - the ability of proteins to access the optimal conformational states linked to function[4]. Since the sites we have identified are not directly involved in ADP binding we suspect conformational flexibility to be affected since mutations often affect the ability of the remote actin and ADP binding sites to communicate[5–7]. In fact, the thermodynamic coupling between actin and ADP binding defines different types of myosin motor activity [8].

Supplementary Information References:

1. Deacon, J. C., Bloemink, M. J., Rezavandi, H., Geeves, M. A. & Leinwand, L. A. Erratum to: Identification of functional differences between recombinant human α and β cardiac myosin motors. *Cell. Mol. Life Sci.* **69**, 4239–55 (2012).

2. Barnes, M. R. & Gray, I. C. *Bioinformatics for geneticists*. (Wiley, 2003).

3. Parker, F. & Peckham, M. Disease mutations in striated muscle myosins. *Biophys. Rev.* **12**, 887–94 (2020).

4. Schmid, S. & Hugel, T. Controlling protein function by fine-tuning conformational flexibility. *Elife* **9**, (2020).

5. Bloemink, M. . *et al.* Alternative exon 9-encoded relay domains affect more than one communication pathway in the Drosophila myosin head. *J. Mol. Biol.* **389**, 707–21 (2009).

6. Bloemink, M., Melkani, G., Dambacher, C., Bernstein, S. & Geeves, M. Two Drosophila myosin transducer mutants with distinct cardiomyopathies have divergent ADP and actin affinities. *J. Biol. Chem.* **286**, 28435–43 (2011).

7. Tsiavaliaris, G. *et al.* Mutations in the relay loop region result in dominant-negative inhibition of myosin II function in Dictyostelium. *EMBO Rep.* **3**, 1099–105 (2002).

8. Bloemink, M. & Geeves, M. Shaking the myosin family tree: Biochemical kinetics defines four types of myosin motor. *Seminars in Cell and Developmental Biology* **22**, 961–967 (2011).
