# Supplementary material for: Identification of sequence changes in myosin II that adjust muscle contraction velocity
Source: PLoS Biol. 2021 Jun 10;19(6):e3001248. doi: 10.1371/journal.pbio.3001248 (PMC8191873; doi:10.1371/journal.pbio.3001248)

A – Tail domains for EMB, β, NMA, IIx

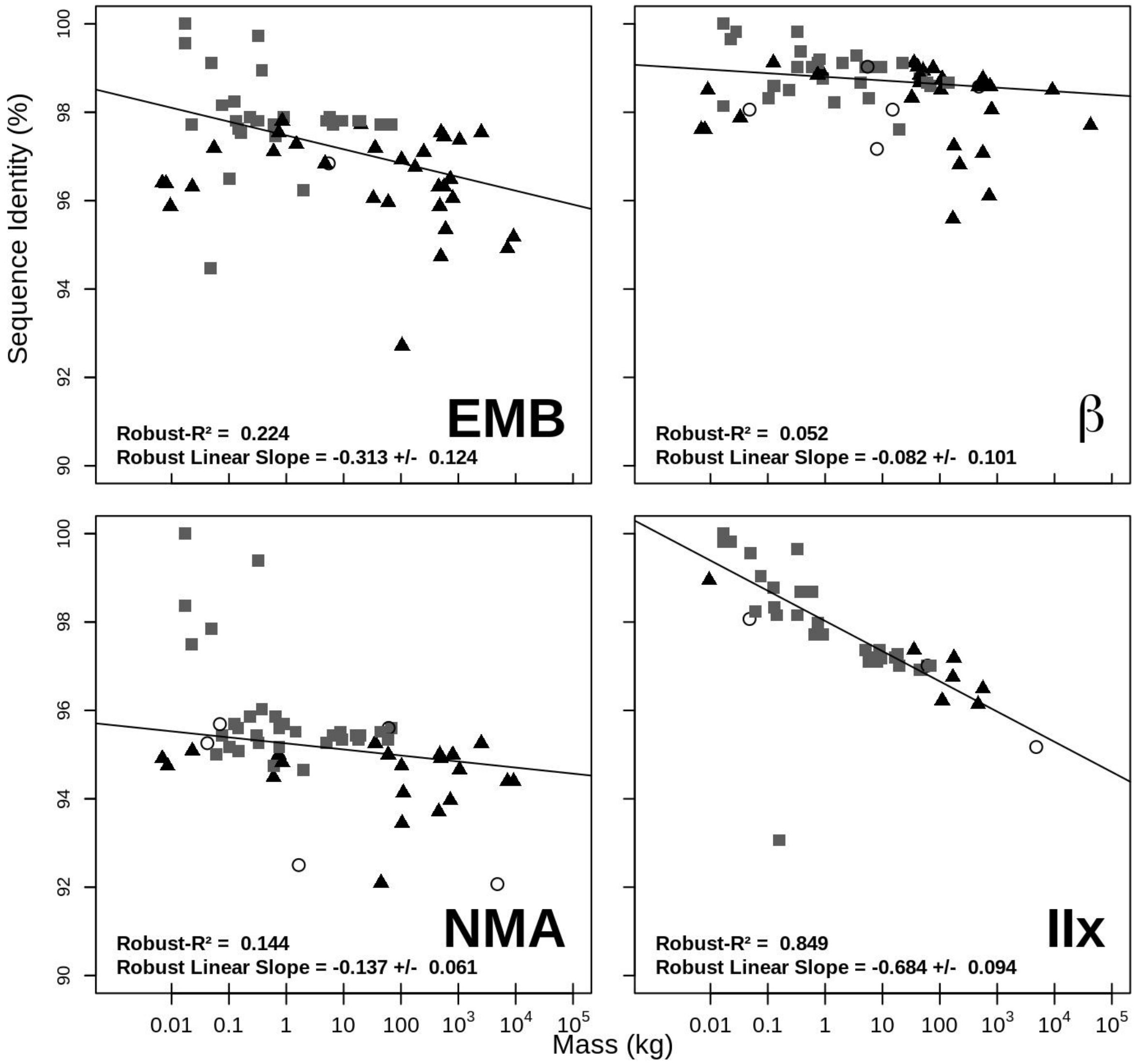

B – Motor and Tail domains for NMB, EXOC and SM.

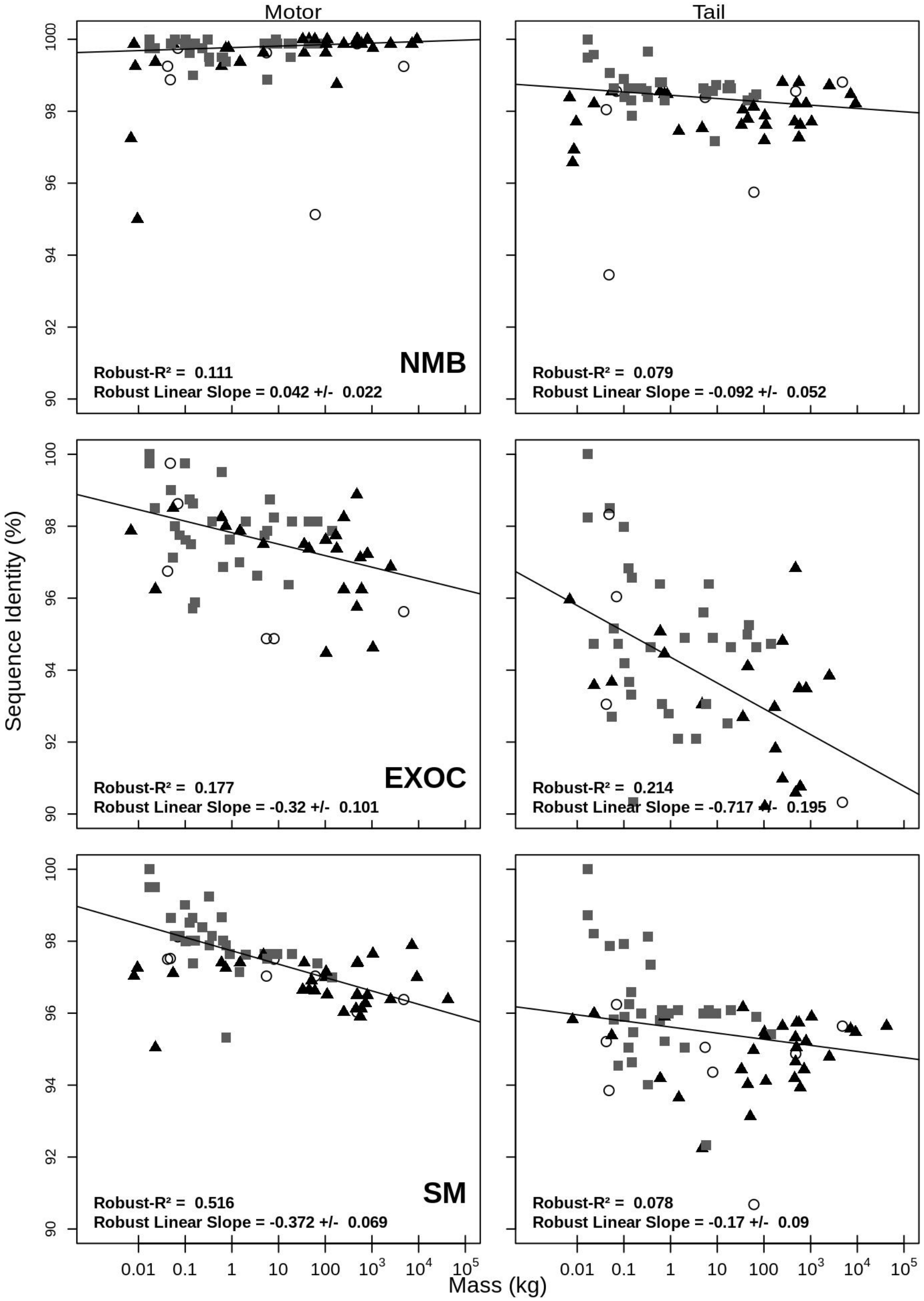

C – Motor and Tail domains for IIa, IIb and NMC

NB   Note change of y-scale for NMC to accommodate the broader spread of values for the NMC tail

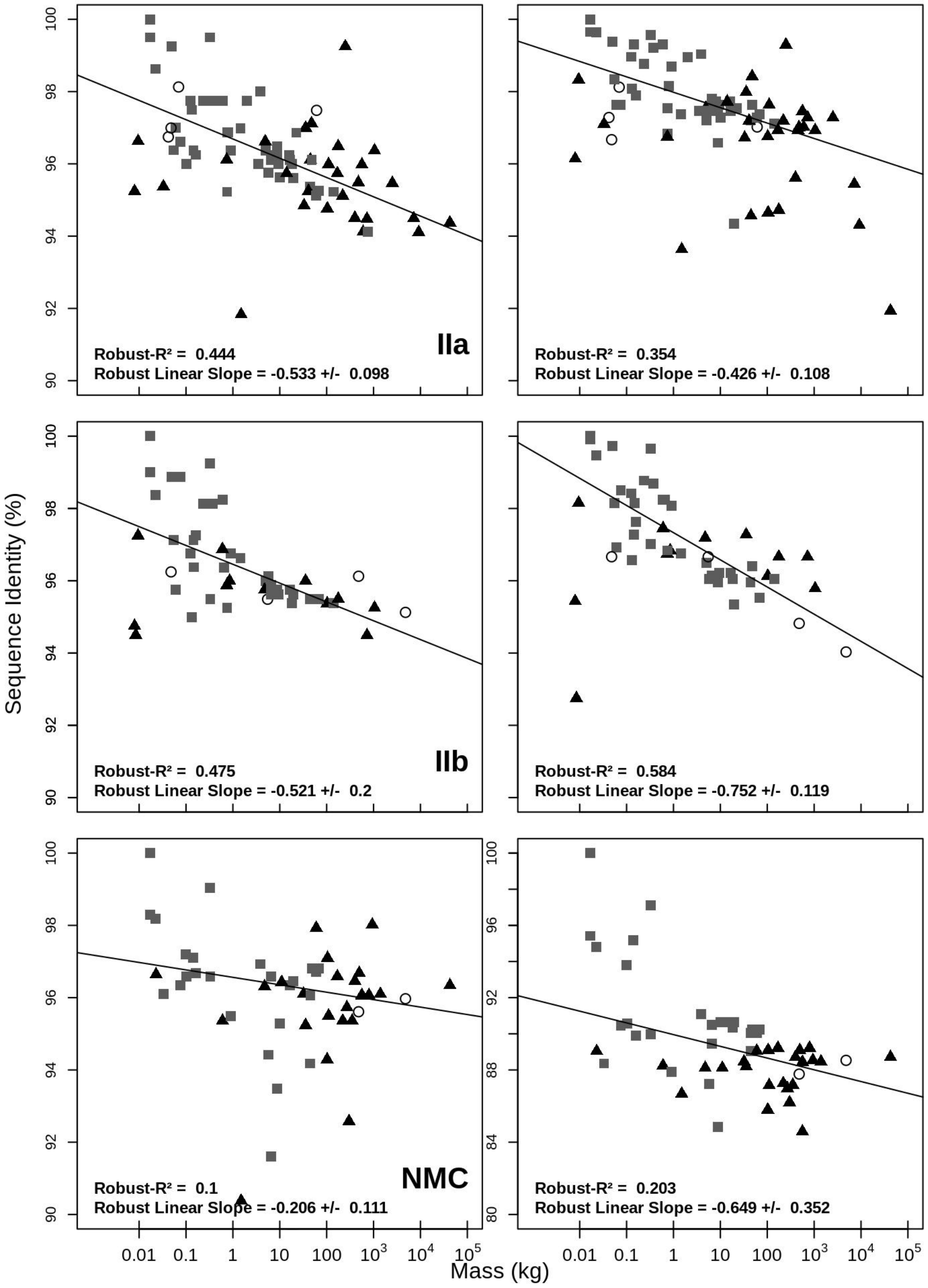

D – Motor and Tail domains for PERI, SlowT, and α

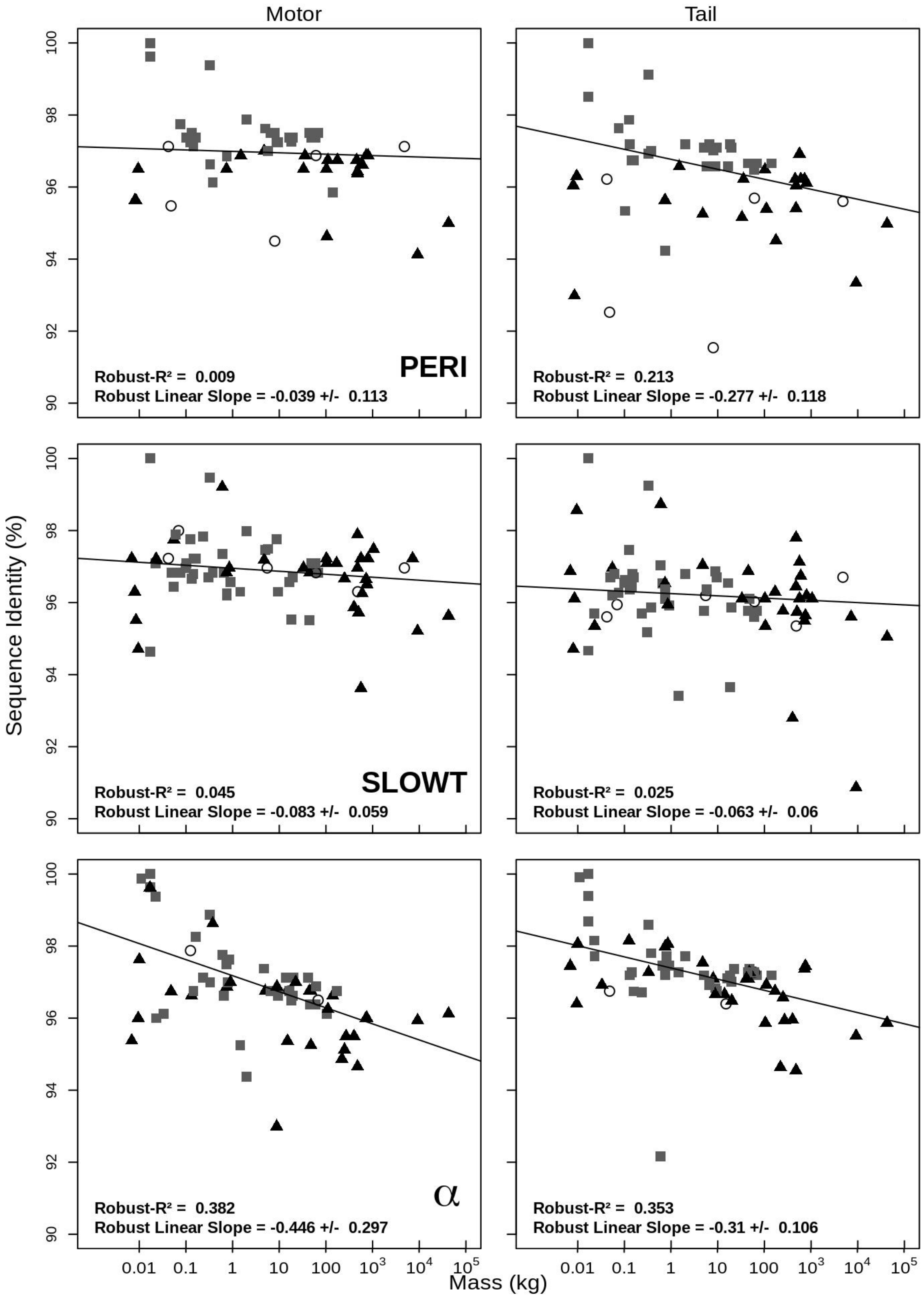

Supplement: S1 Fig — The motor and tail domains have been analysed separately. The grey squares are Euarchontoglires, the black triangles are Laurasiatheria, and the open circles are the Afrotheria and Metatheria groups. Each plot has been fitted with a robust linear regression. Sequence identity is pairwise to the mouse. The R2 value and slope gradient are shown on each plot. Raw data files are available at Figshare. (PDF) [file pbio.3001248.s002.pdf]
