# Supplementary figures and images for: Identification of sequence changes in myosin II that adjust muscle contraction velocity
Source: PLoS Biol. 2021 Jun 10;19(6):e3001248. doi: 10.1371/journal.pbio.3001248 (PMC8191873; doi:10.1371/journal.pbio.3001248)

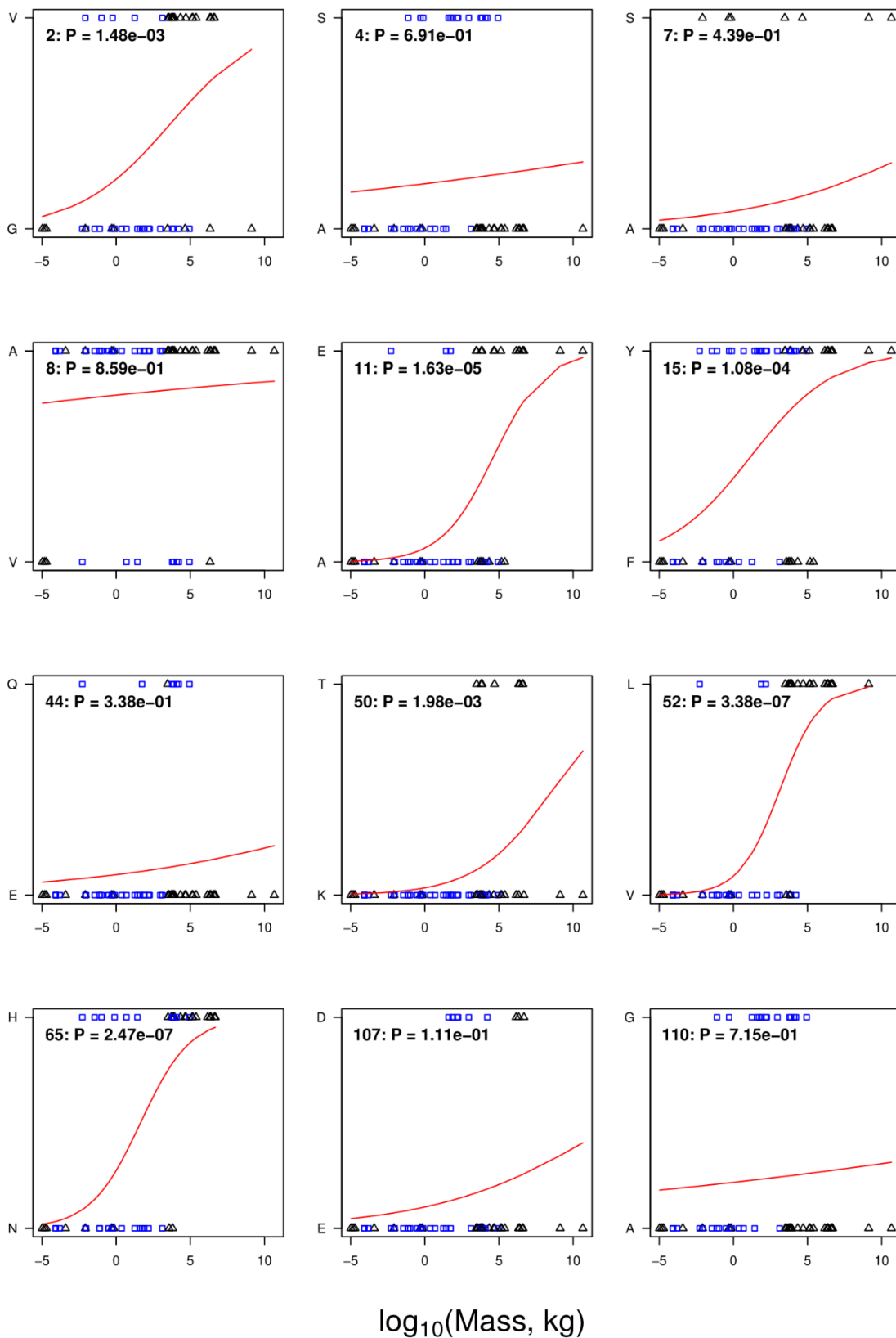

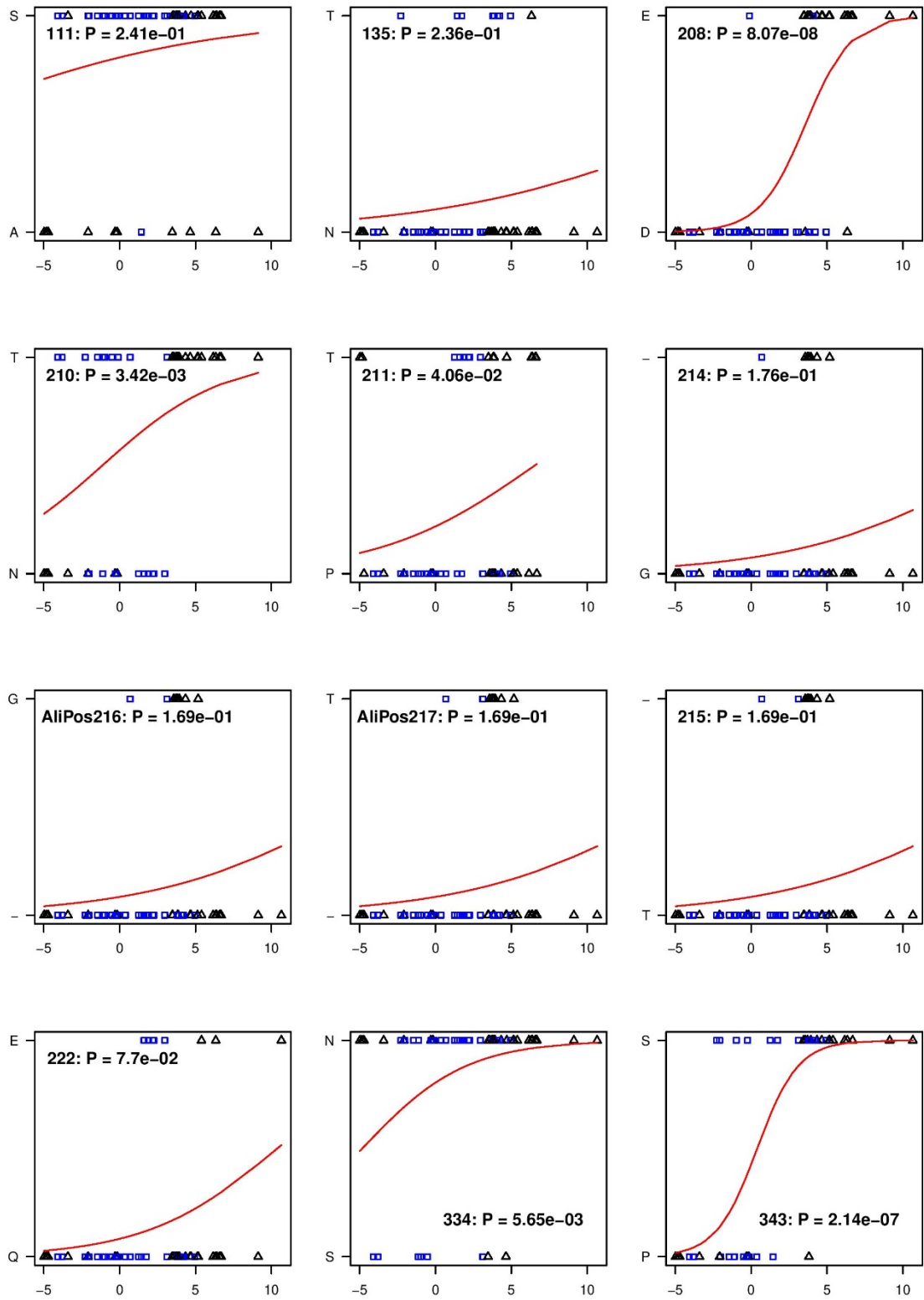

$\log_{10}(\text{Mass, kg})$

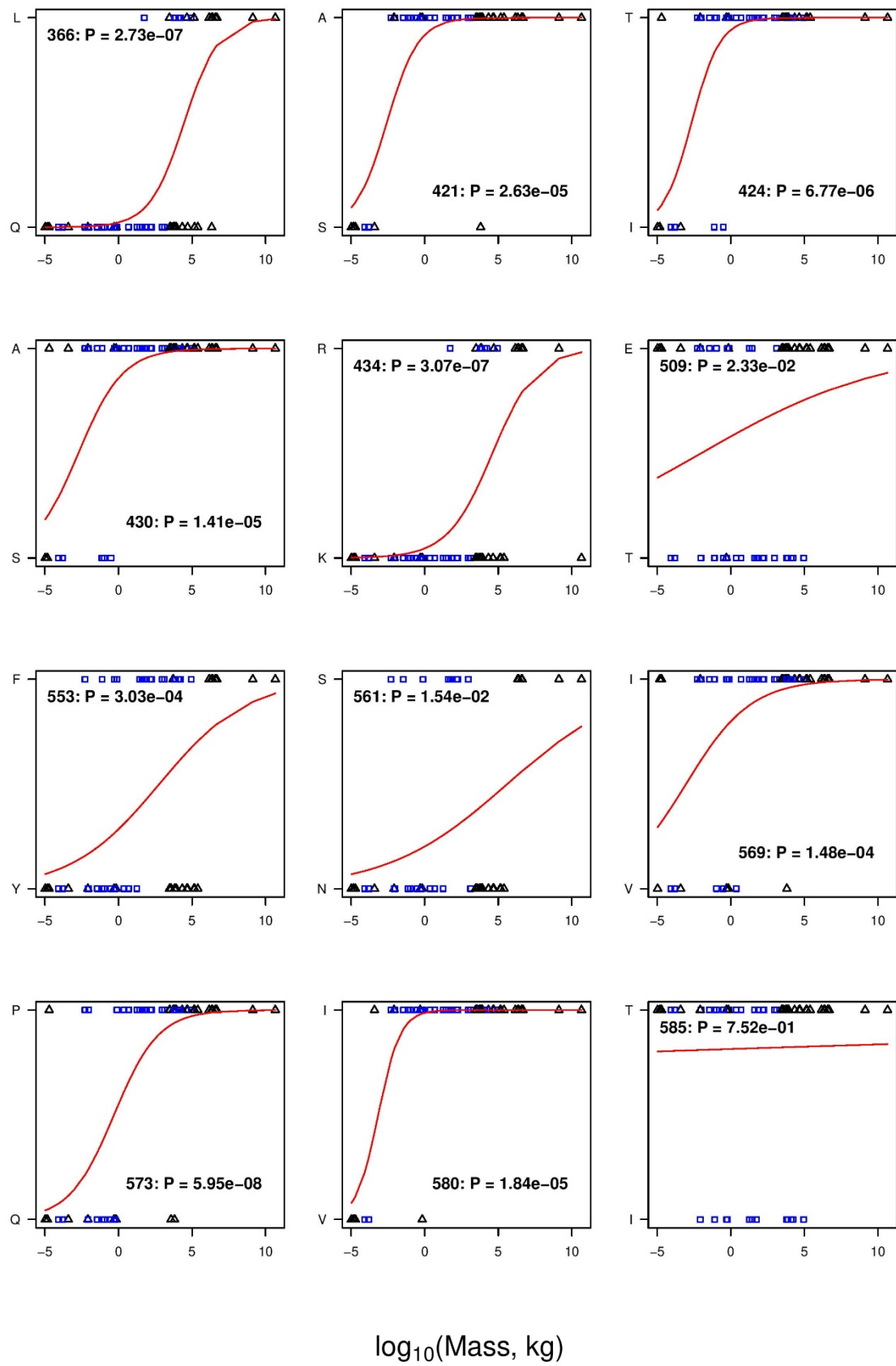

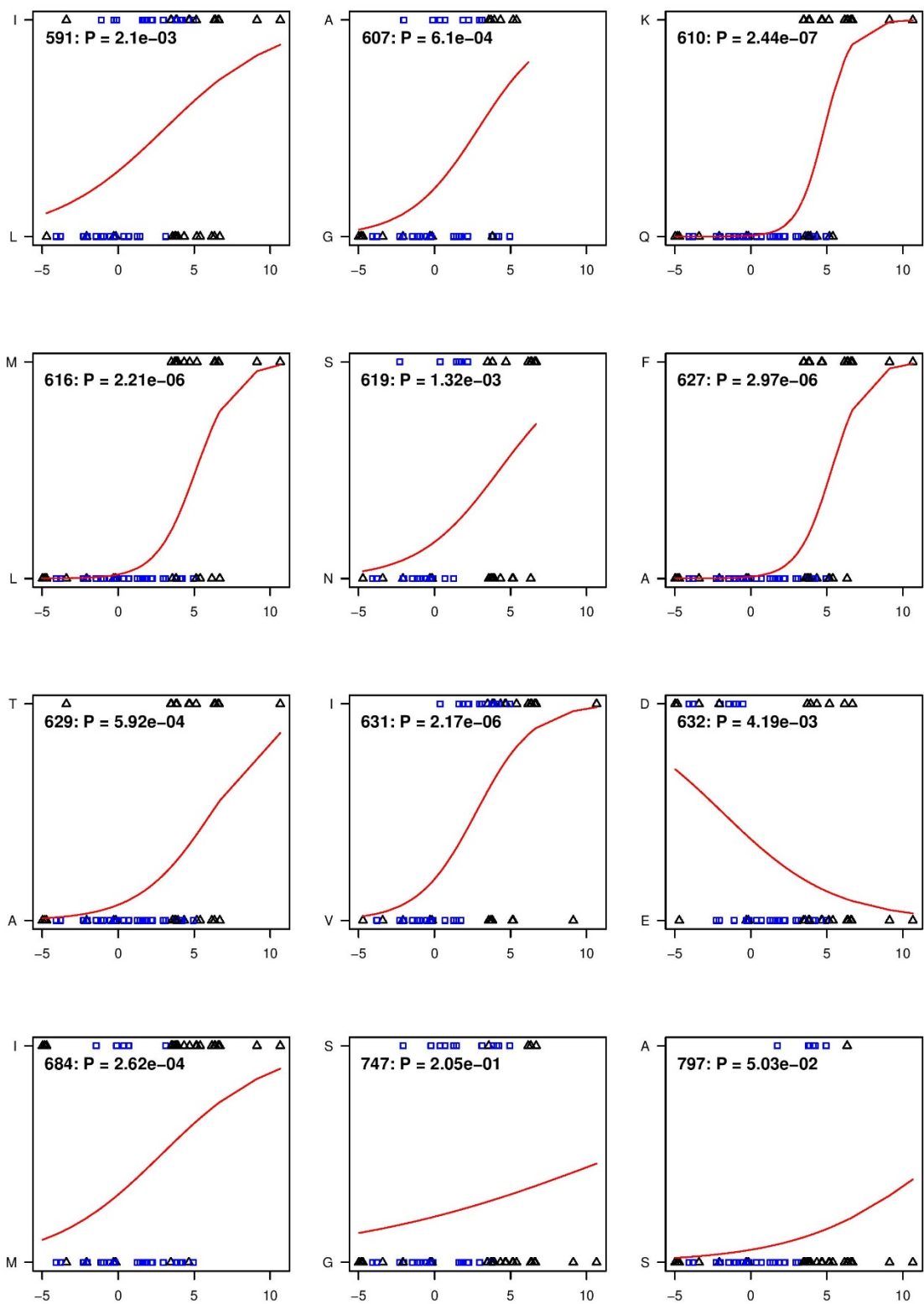

$\log_{10}(\text{Mass, kg})$

Supplement: S2 Fig — Binomial regression mapping the transition of the most frequent amino acid at positions in the motor region of β-myosin to the second most frequent amino acid at that position. The residue numbering is that of the human β-myosin, as oppose to the alignment position. The black squares are Euarchontoglires, and the triangles are Laurasiatheria. The p-value with each plot indicate the probability that the transition of the amino acids is not a result of change in mass. AliPos refers to positions in the sequence alignment that are not present in human β-myosin. Raw data files are available at Figshare. (PDF) [file pbio.3001248.s003.pdf]

Amino Acid

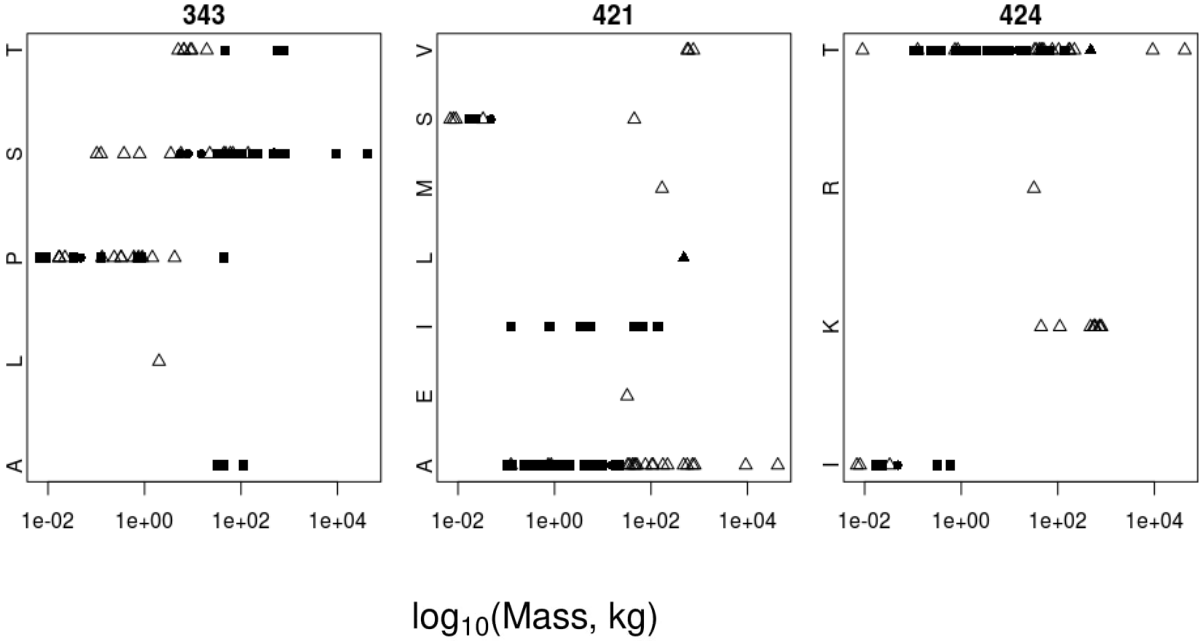

Supplement: S3 Fig — Residues which had more than 2 sites of variation, with the third most frequent amino acid being close in frequency to the second most common amino acid. The black squares are Euarchontoglires, and the triangles are Laurasiatheria. The residue numbering is that of human β-cardiac myosin. Raw data files are available at Figshare. (PDF) [file pbio.3001248.s004.pdf]

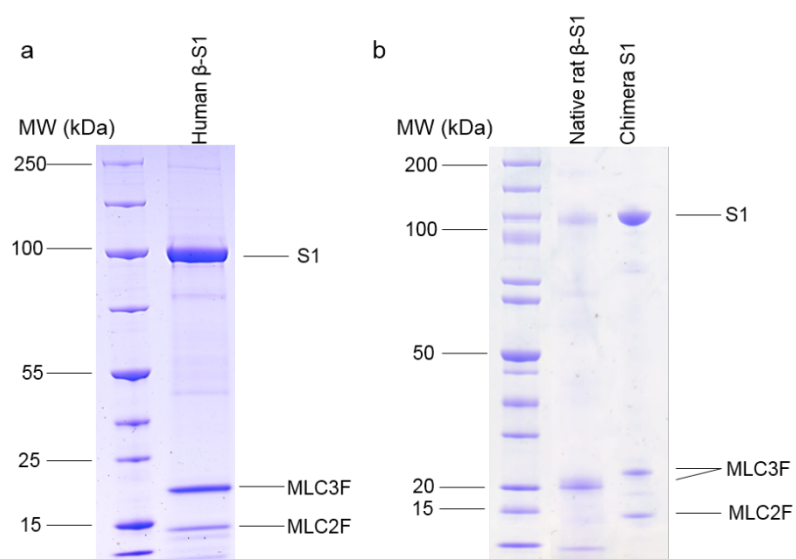

Supplement: S4 Fig — (A) Recombinant human β-S1 with 2 light chains. (B) Native rat β-S1 and recombinant chimera S1 with 2 light chains. (PDF) [file pbio.3001248.s005.pdf]

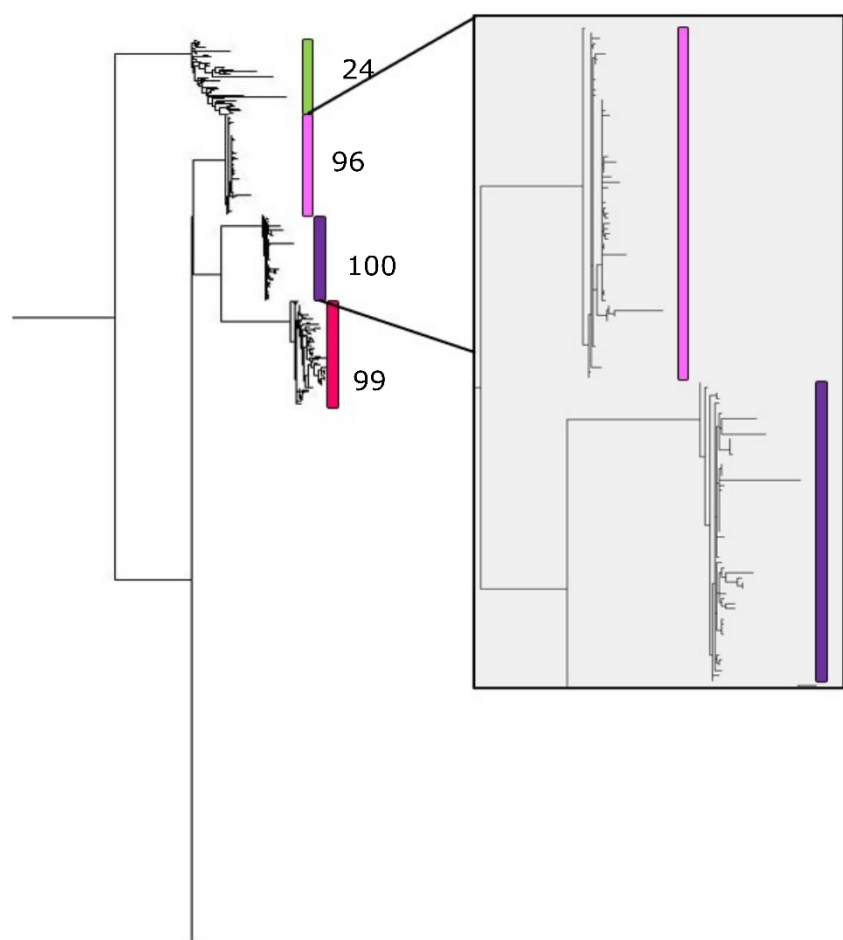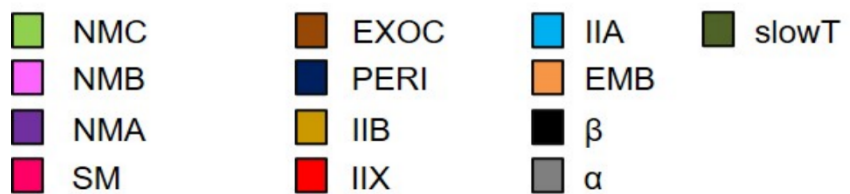

0.5

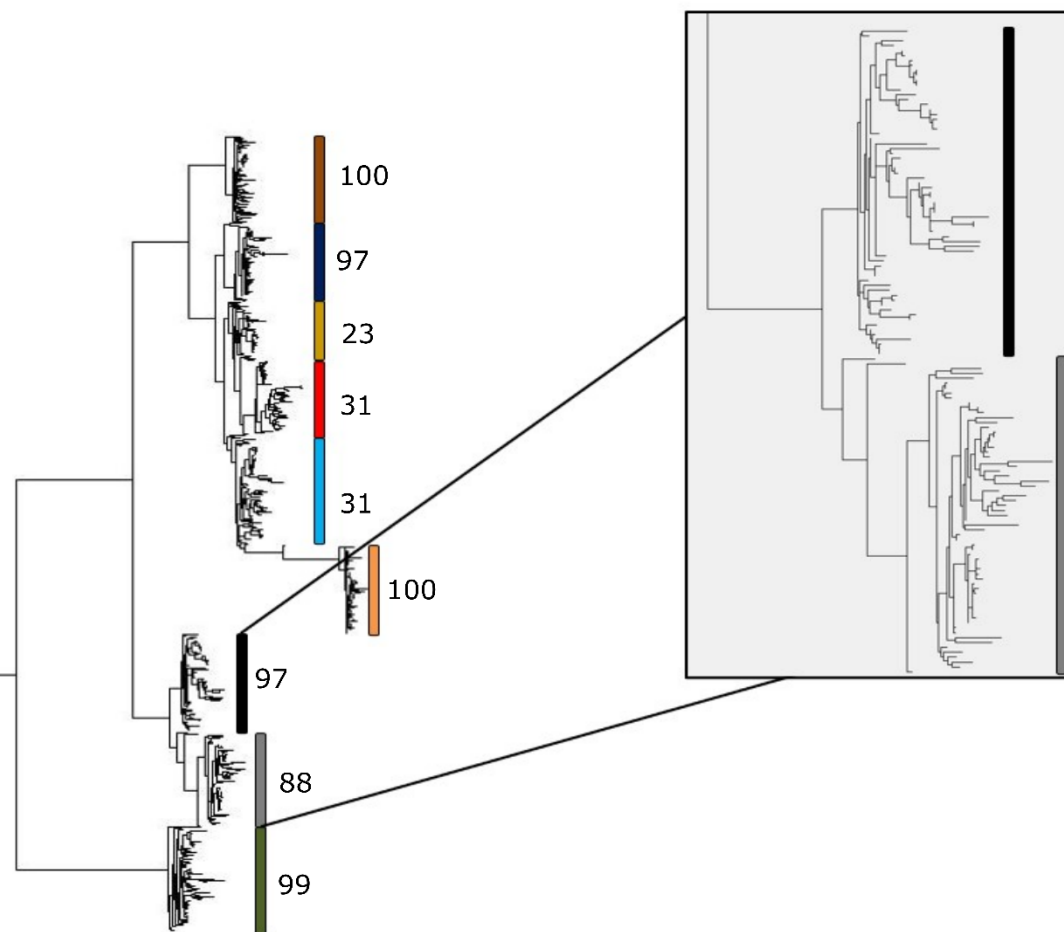

Supplement: S6 Fig — ML phylogenetic tree generated for all myosin isoforms. Isoforms are labelled according to the key. Zoomed in sections show the α and β regions, and the NMA and NMB regions. Bootstrap values for the isoform branches are shown adjacent to the bars identifying each isoform. ML, maximum likelihood. (PDF) [file pbio.3001248.s007.pdf]

## A IIa

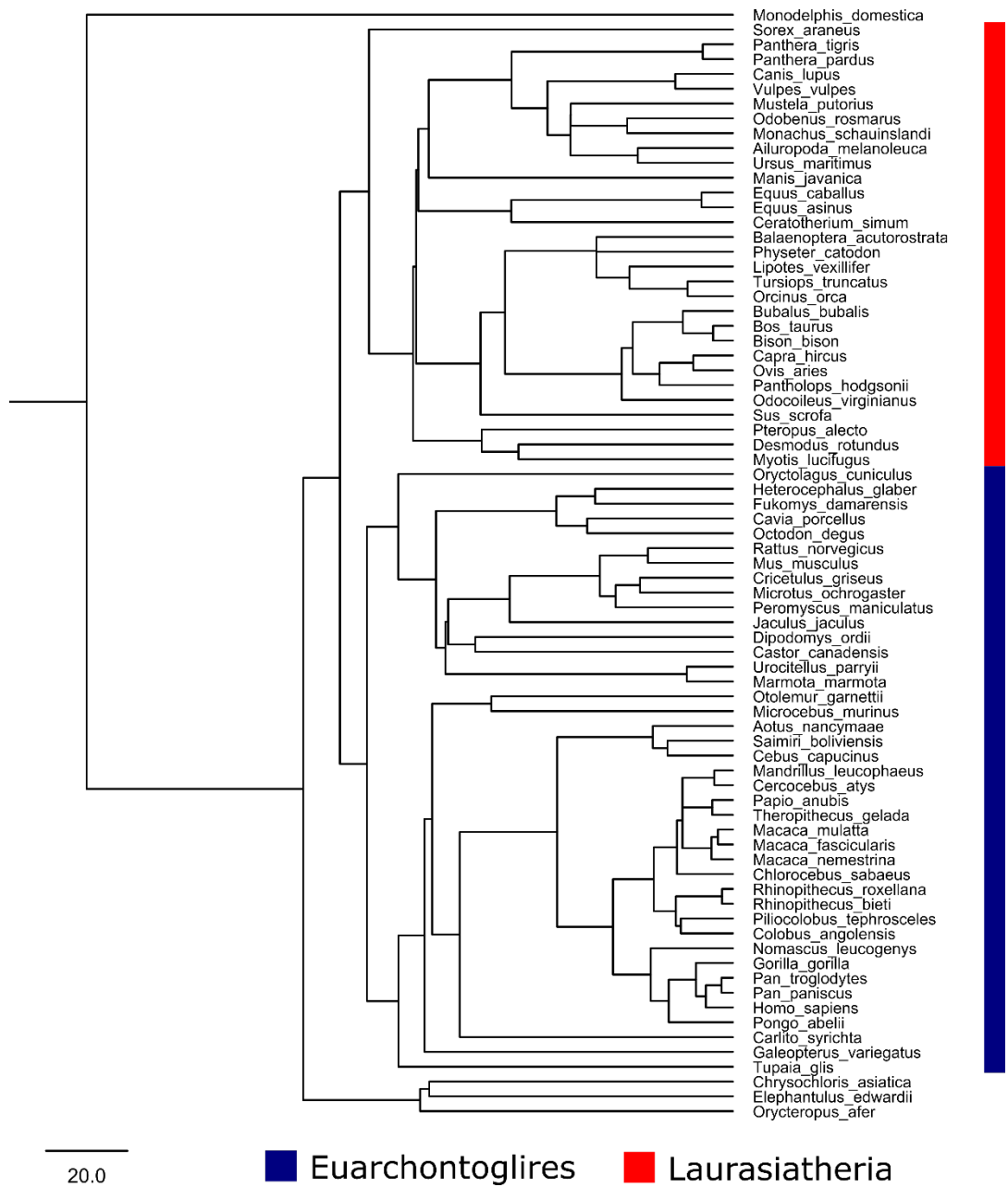

## B IIb

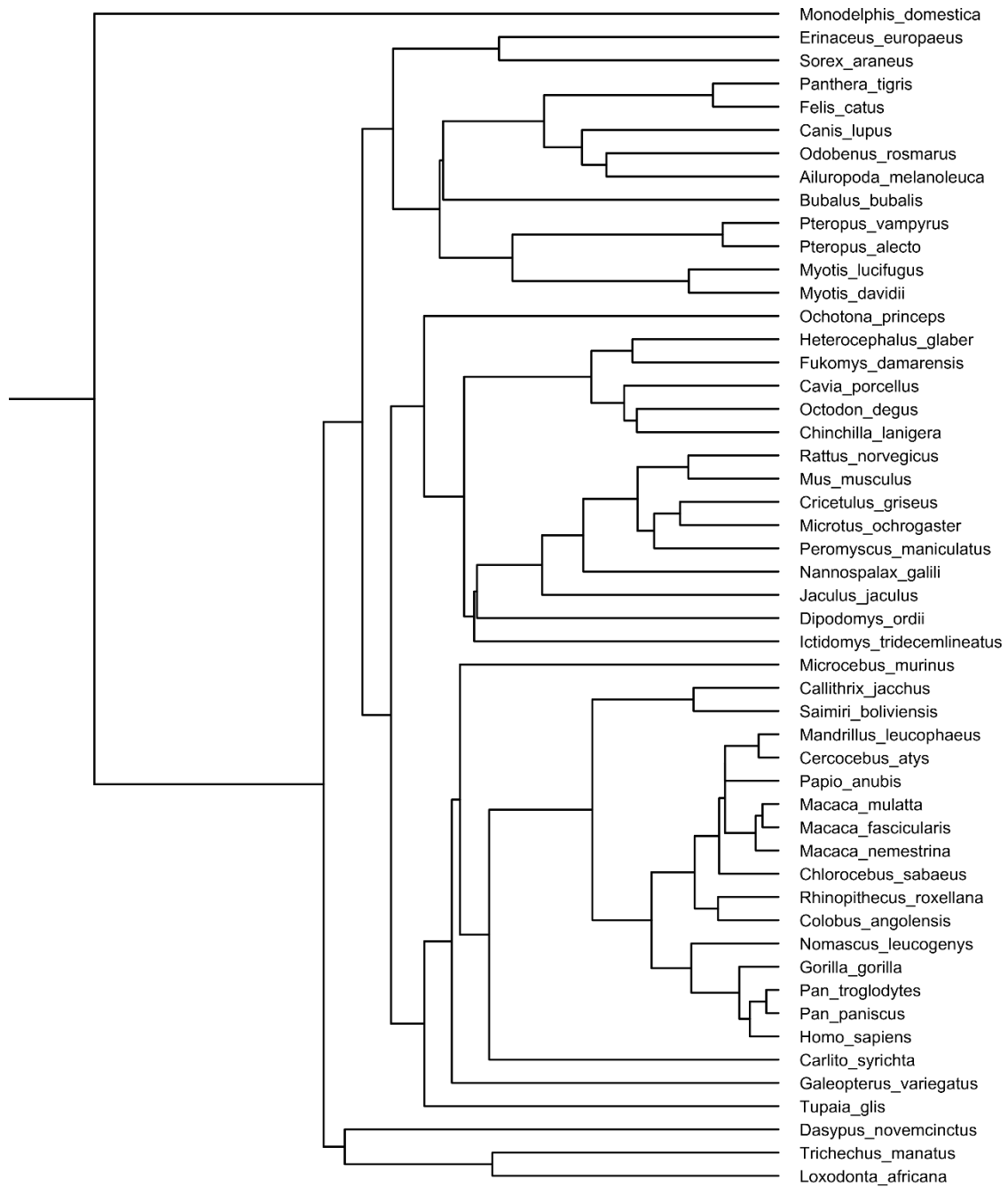

20.0

■ Euarchontoglires ■ Laurasiatheria

C llx

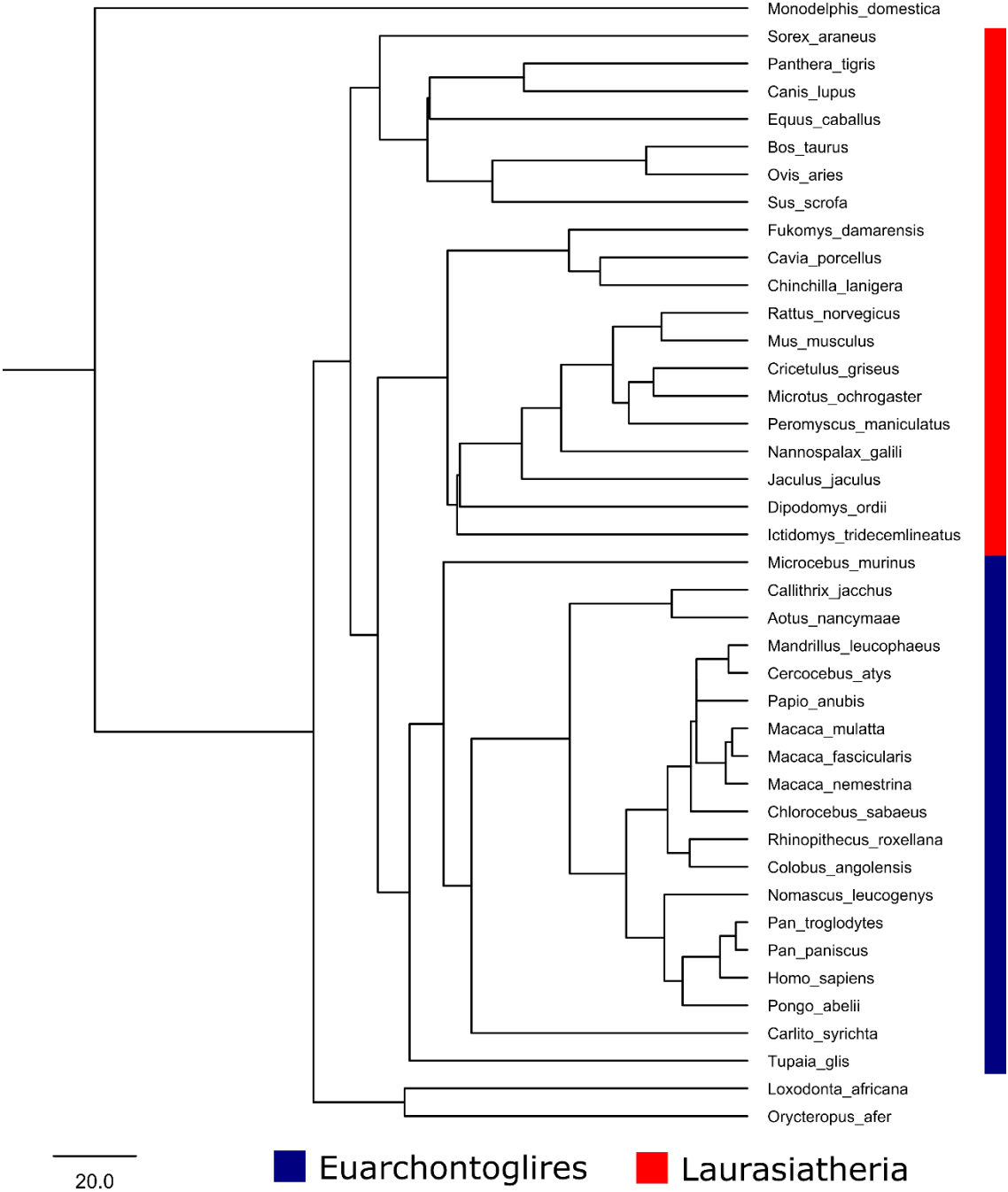

D α

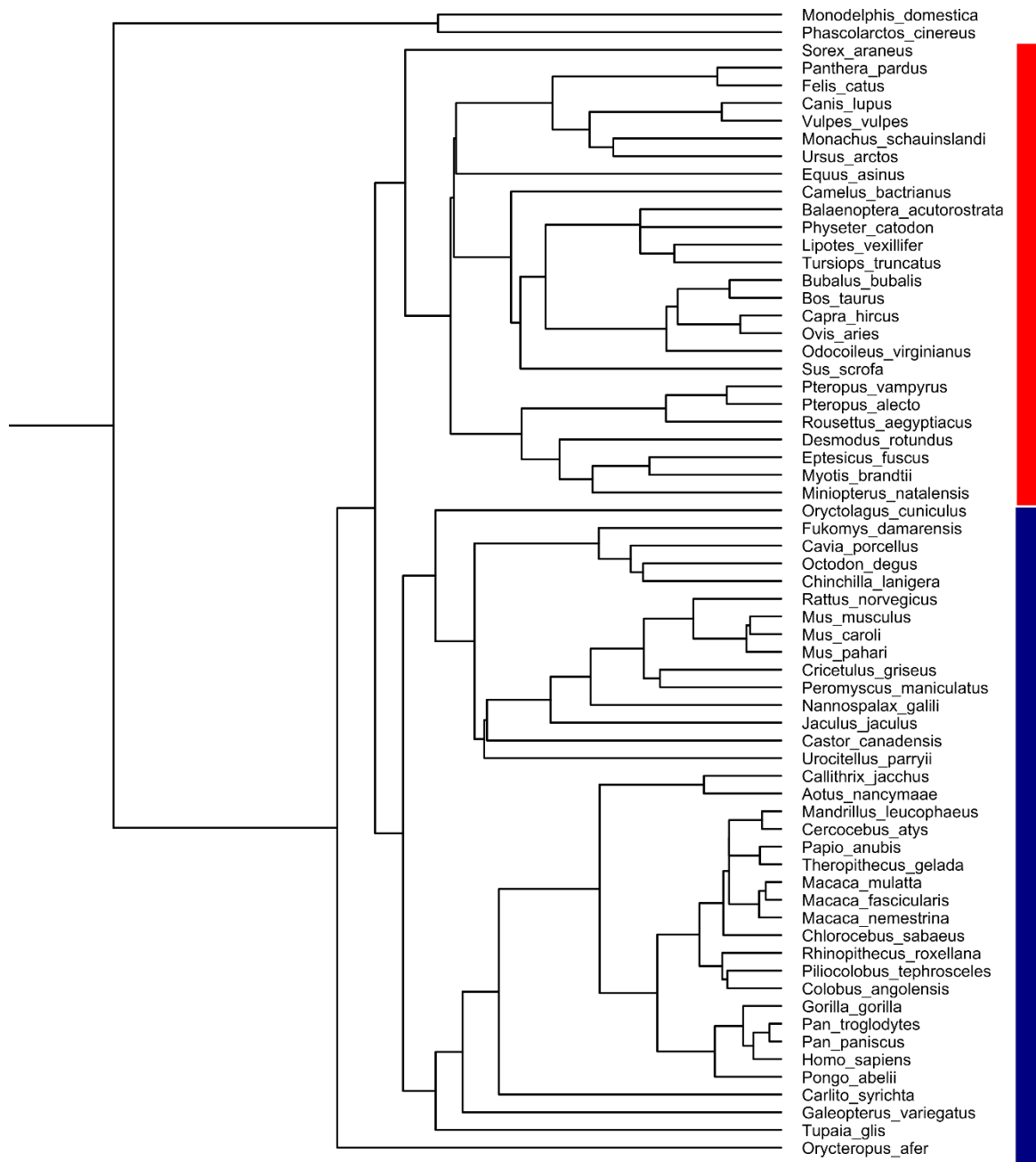

20.0

■ Euarchontoglires ■ Laurasiatheria

E β

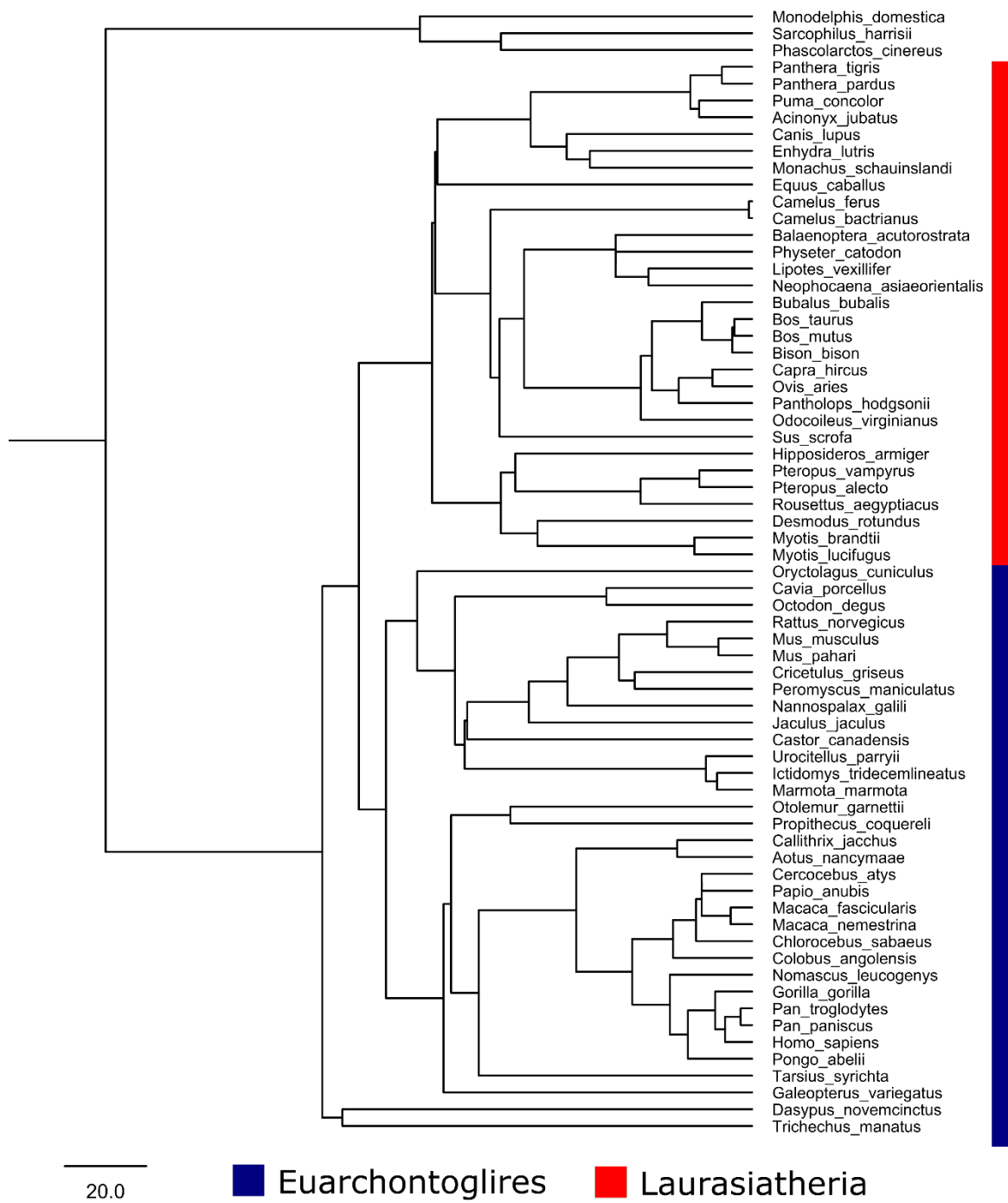

Supplement: S8 Fig — Phylogenetic trees for IIa, IIb, IIx, α, and β species generated from TimeTree. (PDF) [file pbio.3001248.s009.pdf]
