# Supplementary material for: Identification of sequence changes in myosin II that adjust muscle contraction velocity
Source: PLoS Biol. 2021 Jun 10;19(6):e3001248. doi: 10.1371/journal.pbio.3001248 (PMC8191873; doi:10.1371/journal.pbio.3001248)
Supplement: S5 Fig — This table shows the amino acids that occurs in each of the 12 residues, those predicted to be assocaited with mass and that are of interest, in each species, sorted by mass. Yellow background is the predominant amino acid in small mammals. Blue background is the predominant amino acid in large mammals. The clades Laurasiatheria, Euarchontoglires, Metatheria, and Afrotheria are represented by the letter L, E, M, and A, respectively. (PDF) [file pbio.3001248.s006.pdf]

Species	Clade	366	434	326	553	343	349	573	421	424	430	569	580
Myotis_brandtii	L	Q	K	S	Y	P	I	Q	S	I	S	V	V
Myotis_lucifugus	L	Q	K	S	Y	P	I	Q	S	I	S	I	V
Hipposideros_armiger	L	Q	K	S	Y	P	I	P	S	T	A	I	V
Peromyscus_maniculatus_bairdii	E	Q	K	S	Y	P	I	Q	S	I	S	V	V
Mus_musculus	E	Q	K	S	Y	P	I	Q	S	I	S	V	V
Mus_pahari	E	Q	K	S	Y	P	I	Q	S	I	S	V	V
Cricetulus_griseus	L	Q	K	S	Y	P	I	Q	S	I	S	V	V
Desmodus_rotundus	M	Q	K	S	Y	P	I	Q	S	I	A	V	I
Monodelphis_domestica	E	Q	K	S	Y	P	I	Q	S	I	S	I	I
Otolemur_garnettii	E	Q	K	A	F	S	M	P	A	T	A	I	I
Rousettus_aegyptiacus	L	Q	K	S	Y	P	I	Q	A	T	A	I	I
Ictidomys_tridecemlineatus	E	Q	K	S	Y	S	I	Q	I	T	A	I	I
Tarsius_syrichta	E	Q	K	S	Y	P	I	P	A	T	A	I	I
Octodon_degus	E	Q	K	S	Y	P	I	Q	A	T	A	I	I
Rattus_norvegicus	E	Q	K	S	Y	P	I	Q	A	I	S	I	I
Callithrix_jacchus	E	Q	K	S	F	P	M	Q	A	T	A	I	I
Nannospalax_galili	E	Q	K	S	Y	S	I	Q	A	T	S	V	I
Jaculus_jaculus	E	Q	K	S	Y	P	I	Q	A	I	S	V	I
Pteropus_alecto	E	Q	K	A	Y	P	I	Q	A	T	A	V	I
Aotus_nancymaae	E	Q	K	S	F	P	M	Q	A	T	A	I	I
Urocitellus_parryii	E	Q	K	S	Y	S	I	Q	I	T	A	I	I
Pteropus_vampyrus	L	Q	K	A	Y	P	I	Q	A	T	A	V	V
Cavia_porcellus	E	Q	K	S	F	P	M	P	A	T	A	I	I
Galeopterus_variegatus	E	M	K	S	Y	P	M	P	A	T	A	V	I
Oryctolagus_cuniculus	E	Q	K	S	Y	L	M	P	A	T	A	I	I
Marmota_marmota_marmota	E	Q	K	S	Y	S	I	P	I	T	A	I	I
Propithecus_coquereli	E	Q	K	A	F	P	M	P	A	T	A	I	I
Chlorocebus_sabaeus	E	Q	K	A	F	T	M	P	A	T	A	I	I
Dasypus_novemcinctus	M	Q	K	A	Y	S	I	P	A	T	A	I	I
Nomascus_leucogenys	E	L	R	A	F	S	I	P	I	T	A	I	I
Macaca_fascicularis	E	Q	K	A	F	T	M	P	A	T	A	I	I
Macaca_nemestrina	E	Q	K	A	F	T	M	P	A	T	A	I	I
Sarcophilus_harrisii	M	Q	K	S	Y	S	M	P	A	T	S	I	I
Colobus_angolensis_palliatus	E	Q	K	A	F	T	M	P	A	T	A	I	I
Cercocebus_atys	E	Q	K	A	F	T	M	P	A	T	A	I	I
Phascolarctos_cinereus	M	L	K	S	F	S	I	P	A	T	A	I	I
Papio_anubis	E	Q	K	S	F	S	M	P	A	T	A	I	I
Castor_canadensis	E	Q	K	S	F	S	M	P	A	T	A	I	I
Neophocaena_asiaeorientalis_asiaeorientalis	L	L	K	A	Y	S	I	P	E	R	A	I	I
Pantholops_hodgsonii	L	Q	R	A	Y	A	M	P	A	T	A	I	I
Canis_lupus_familiaris	L	Q	K	S	Y	S	M	Q	A	T	A	I	I
Panthera_pardus	L	Q	K	A	F	S	M	P	A	T	A	I	I
Pan_paniscus	E	L	R	A	F	S	M	P	I	T	A	I	I
Capra_hircus	L	Q	R	A	Y	A	M	P	A	K	A	I	I
Enhydra_lutris_kenyoni	L	Q	K	S	Y	P	M	Q	S	T	A	V	I
Odocoileus_virginianus_texanus	L	Q	R	A	Y	T	M	P	A	T	A	I	I
Pan_troglodytes	E	L	R	A	F	S	M	P	I	T	A	I	I
Acinonyx_jubatus	L	Q	K	A	Y	S	M	P	A	T	A	I	I
Pongo_abelii	E	L	R	A	F	S	M	P	I	T	A	I	I
Homo_sapiens	E	L	R	A	F	S	M	P	I	T	A	I	I
Puma_concolor	L	Q	K	A	Y	S	M	P	A	T	A	I	I
Lipotes_vexillifer	L	L	K	A	Y	S	I	P	A	T	A	I	I
Ovis_aries	L	Q	R	A	Y	A	M	P	A	K	A	I	I
Gorilla_gorilla_gorilla	E	L	R	A	F	S	M	P	I	T	A	I	I
Sus_scrofa	L	L	K	A	Y	S	M	P	M	T	A	I	I
Panthera_tigris_altaica	L	Q	K	A	Y	S	M	P	A	T	A	I	I
Neomonachus_schauinslandi	L	Q	K	A	Y	S	M	P	A	T	A	I	I
Camelus_bactrianus	L	L	R	A	F	S	M	P	A	K	A	I	I
Trichechus_manatus_latirostris	A	L	R	A	F	S	M	P	L	T	A	I	I
Bos_mutus	L	L	R	A	F	T	M	P	V	K	A	I	I
Equus_caballus	L	Q	R	A	F	S	M	P	A	K	A	I	I
Bison_bison_bison	L	L	R	A	F	T	M	P	V	K	A	I	I
Bubalus_bubalis	L	L	R	A	F	T	M	P	A	K	A	I	I
Bos_taurus	L	L	R	A	F	T	M	P	V	K	A	I	I
Camelus_ferus	L	L	R	A	F	S	M	P	A	K	A	I	I
Balaenoptera_acutorostrata_scammoni	L	L	R	A	F	S	M	P	A	T	A	I	I
Physeter_catodon	L	L	K	A	F	S	M	P	A	T	A	I	I
