# Supplementary material for: Identification of sequence changes in myosin II that adjust muscle contraction velocity
Source: PLoS Biol. 2021 Jun 10;19(6):e3001248. doi: 10.1371/journal.pbio.3001248 (PMC8191873; doi:10.1371/journal.pbio.3001248)
Supplement: S7 Fig — Phylogenetic trees for IIa, IIb, IIx, α, and β isoforms motor and tail domains. (PDF) [file pbio.3001248.s008.pdf]

A IIa Motor

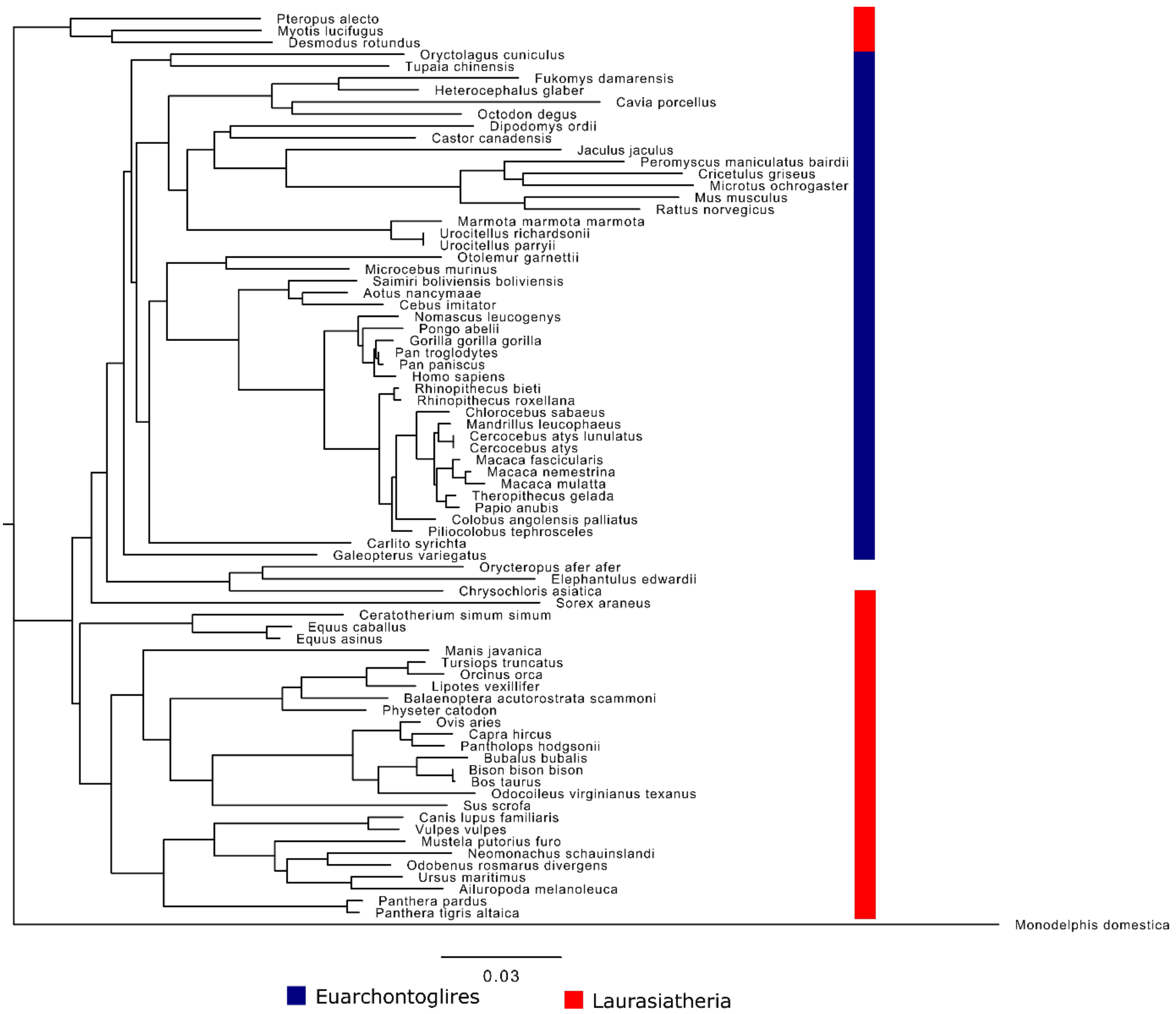
Pteropus alecto
Myotis lucifugus
Desmodus rotundus
Oryctolagus cuniculus
Tupaia chinensis
Fukomys damarensis
Heterocephalus glaber
Cavia porcellus
Octodon degus
Dipodomys ordii
Castor canadensis
Jaculus jaculus
Peromyscus maniculatus bairdii
Cricetulus griseus
Microtus ochrogaster
Mus musculus
Rattus norvegicus
Marmota marmota marmota
Urocitellus richardsonii
Urocitellus parryii
Otolemur garnettii
Microcebus murinus
Saimiri boliviensis boliviensis
Aotus nancymaae
Cebus imitator
Nomascus leucogenys
Pongo abelii
Gorilla gorilla gorilla
Pan troglodytes
Pan paniscus
Homo sapiens
Rhinopithecus bieti
Rhinopithecus roxellana
Chlorocebus sabaeus
Mandrillus leucophaeus
Cercocebus atys lunulatus
Cercocebus atys
Macaca fascicularis
Macaca nemestrina
Macaca mulatta
Theropithecus gelada
Papio anubis
Colobus angolensis palliatus
Piliocolobus tephrosceles
Carlito syrichta
Galeopterus variegatus
Orycteropus afer afer
Elephantulus edwardii
Chrysochloris asiatica
Sorex araneus
Ceratotherium simum simum
Equus caballus
Equus asinus
Manis javanica
Tursiops truncatus
Orcinus orca
Lipotes vexillifer
Balaenoptera acutorostrata scammoni
Physeter catodon
Ovis aries
Capra hircus
Pantholops hodgsonii
Bubalus bubalis
Bison bison bison
Bos taurus
Odocoileus virginianus texanus
Sus scrofa
Canis lupus familiaris
Vulpes vulpes
Mustela putorius furo
Neomonachus schauinslandi
Odobenus rosmarus divergens
Ursus maritimus
Ailuropoda melanoleuca
Panthera pardus
Panthera tigris altaica
Monodelphis domestica
0.03
Euarchontoglires
Laurasiatheria

B IIa Tail

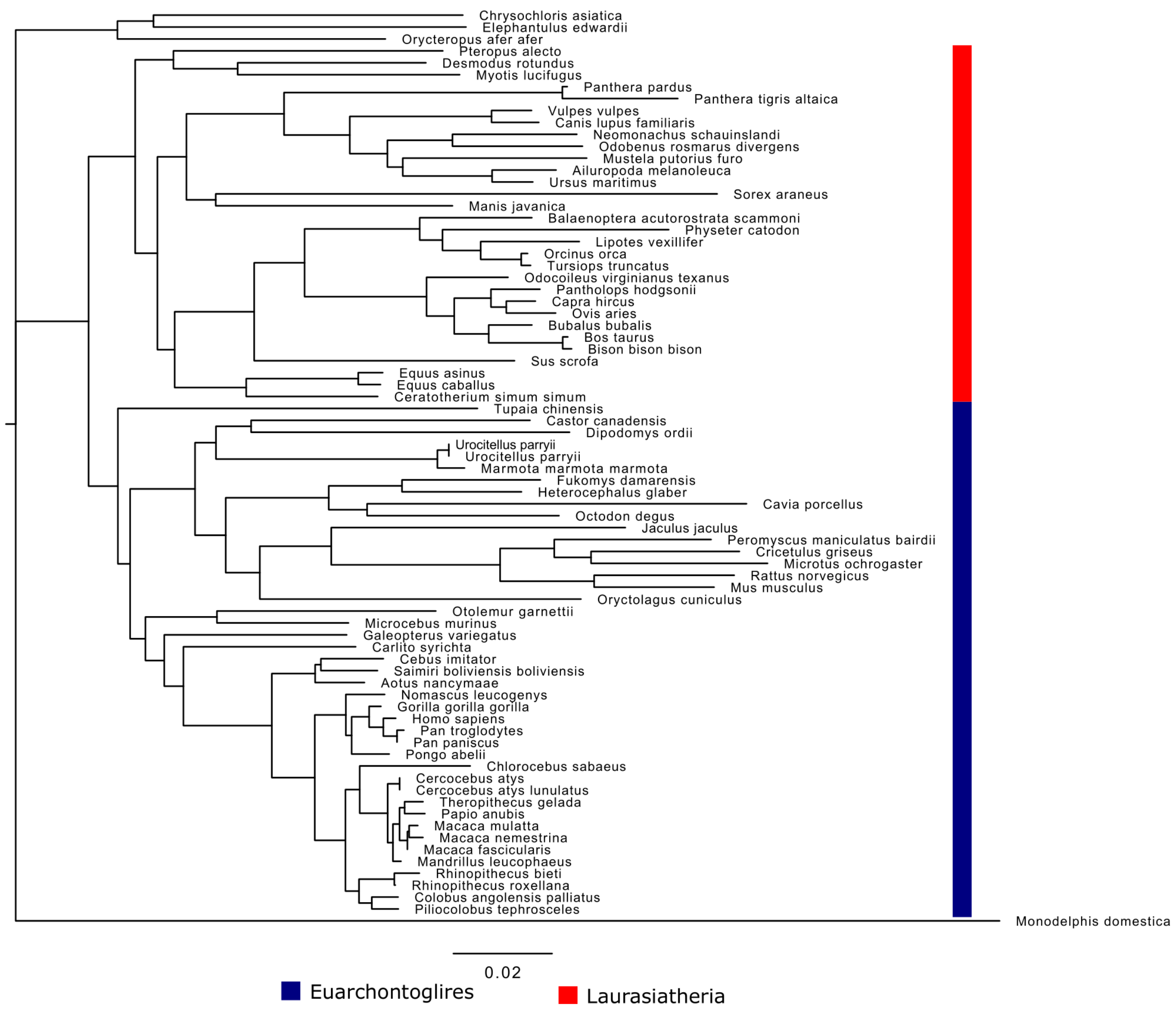

Chrysochloris asiatica
Elephantulus edwardii
Orycteropus afer afer
Pteropus alecto
Desmodus rotundus
Myotis lucifugus
Panthera pardus
Panthera tigris altaica
Vulpes vulpes
Canis lupus familiaris
Neomonachus schauinslandi
Odobenus rosmarus divergens
Mustela putorius furo
Ailuropoda melanoleuca
Ursus maritimus
Sorex araneus
Manis javanica
Balaenoptera acutorostrata scammoni
Physeter catodon
Lipotes vexillifer
Orcinus orca
Tursiops truncatus
Odocoileus virginianus texanus
Pantholops hodgsonii
Capra hircus
Ovis aries
Bubalus bubalis
Bos taurus
Bison bison bison
Sus scrofa
Equus asinus
Equus caballus
Ceratotherium simum simum
Tupaia chinensis
Castor canadensis
Dipodomys ordii
Urocitellus parryii
Urocitellus parryii
Marmota marmota marmota
Fukomys damarensis
Heterocephalus glaber
Cavia porcellus
Octodon degus
Jaculus jaculus
Peromyscus maniculatus bairdii
Cricetulus griseus
Microtus ochrogaster
Rattus norvegicus
Mus musculus
Oryctolagus cuniculus
Otolemur garnettii
Microcebus murinus
Galeopterus variegatus
Carlito syrichta
Cebus imitator
Saimiri boliviensis boliviensis
Aotus nancymaae
Nomascus leucogenys
Gorilla gorilla gorilla
Homo sapiens
Pan troglodytes
Pan paniscus
Pongo abelii
Chlorocebus sabaeus
Cercocebus atys
Cercocebus atys lunulatus
Theropithecus gelada
Papio anubis
Macaca mulatta
Macaca nemestrina
Macaca fascicularis
Mandrillus leucophaeus
Rhinopithecus bieti
Rhinopithecus roxellana
Colobus angolensis palliatus
Piliocolobus tephrosceles
Monodelphis domestica
0.02
Euarchontoglires
Laurasiatheria

C IIb Motor

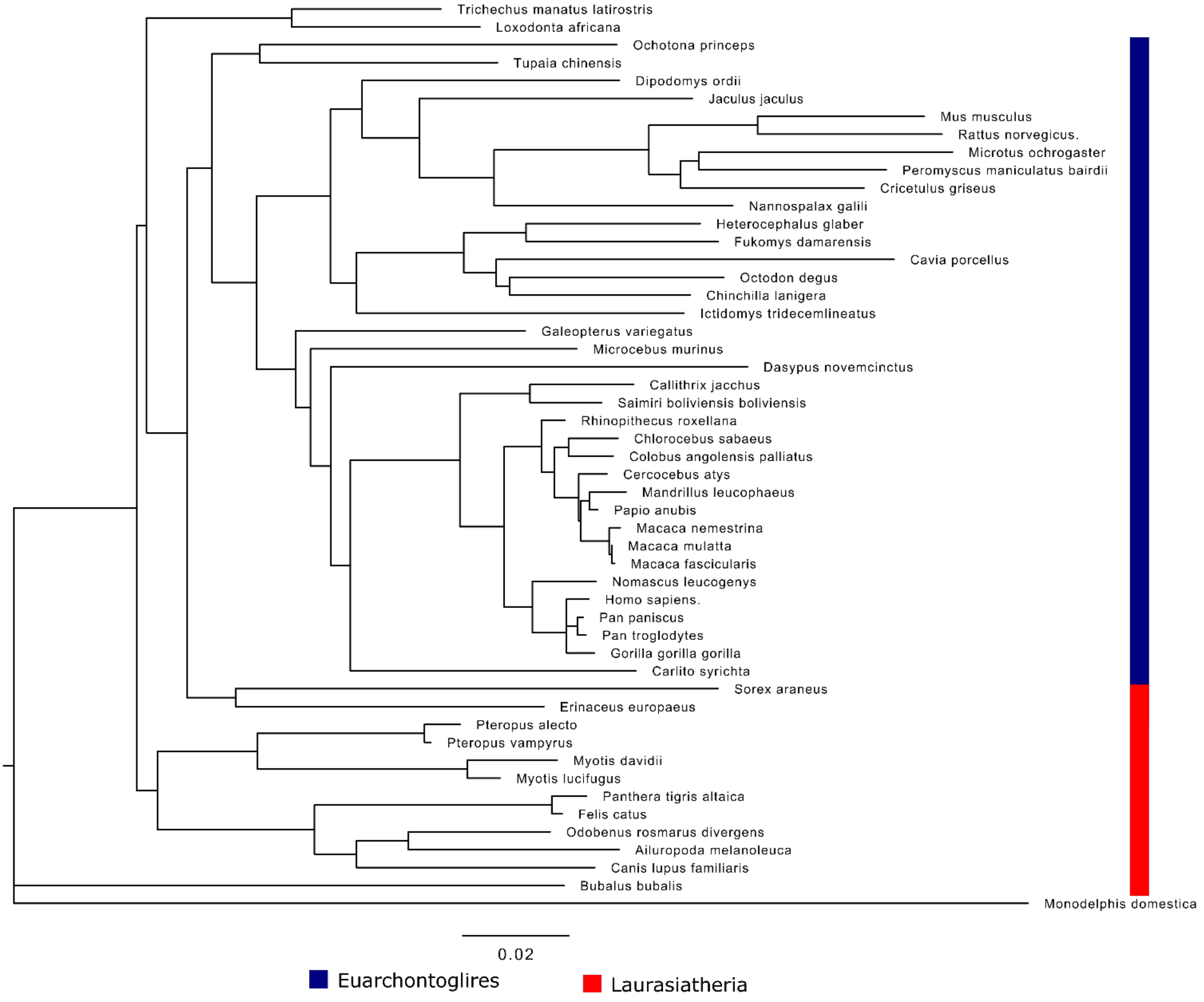
Trichechus manatus latirostris
Loxodonta africana
Ochotona princeps
Tupaia chinensis
Dipodomys ordii
Jaculus jaculus
Mus musculus
Rattus norvegicus.
Microtus ochrogaster
Peromyscus maniculatus bairdii
Cricetulus griseus
Nannospalax galili
Heterocephalus glaber
Fukomys damarensis
Cavia porcellus
Octodon degus
Chinchilla lanigera
Ictidomys tridecemlineatus
Galeopterus variegatus
Microcebus murinus
Dasypus novemcinctus
Callithrix jacchus
Saimiri boliviensis boliviensis
Rhinopithecus roxellana
Chlorocebus sabaeus
Colobus angolensis palliatus
Cercocebus atys
Mandrillus leucophaeus
Papio anubis
Macaca nemestrina
Macaca mulatta
Macaca fascicularis
Nomascus leucogenys
Homo sapiens.
Pan paniscus
Pan troglodytes
Gorilla gorilla gorilla
Carlito syrichta
Sorex araneus
Erinaceus europaeus
Pteropus alecto
Pteropus vampyrus
Myotis davidii
Myotis lucifugus
Panthera tigris altaica
Felis catus
Odobenus rosmarus divergens
Ailuropoda melanoleuca
Canis lupus familiaris
Bubalus bubalis
Monodelphis domestica
0.02
Euarchontoglires
Laurasiatheria

D IIb Tail

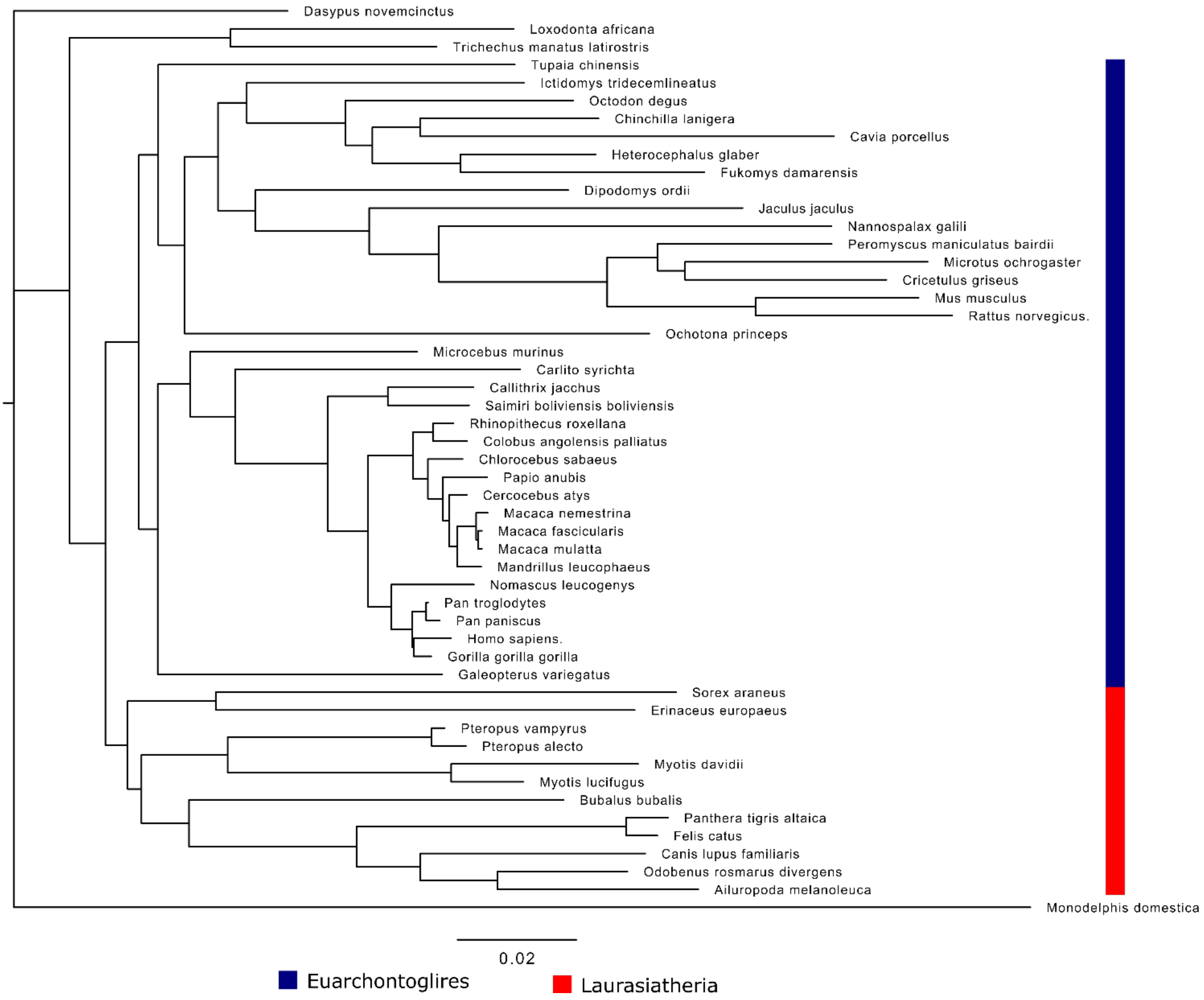
Dasypus novemcinctus
Loxodonta africana
Trichechus manatus latirostris
Tupaia chinensis
Ictidomys tridecemlineatus
Octodon degus
Chinchilla lanigera
Cavia porcellus
Heterocephalus glaber
Fukomys damarensis
Dipodomys ordii
Jaculus jaculus
Nannospalax galili
Peromyscus maniculatus bairdii
Microtus ochrogaster
Cricetulus griseus
Mus musculus
Rattus norvegicus.
Ochotona princeps
Microcebus murinus
Carlito syrichta
Callithrix jacchus
Saimiri boliviensis boliviensis
Rhinopithecus roxellana
Colobus angolensis palliatus
Chlorocebus sabaeus
Papio anubis
Cercocebus atys
Macaca nemestrina
Macaca fascicularis
Macaca mulatta
Mandrillus leucophaeus
Nomascus leucogenys
Pan troglodytes
Pan paniscus
Homo sapiens.
Gorilla gorilla gorilla
Galeopterus variegatus
Sorex araneus
Erinaceus europaeus
Pteropus vampyrus
Pteropus alecto
Myotis davidii
Myotis lucifugus
Bubalus bubalis
Panthera tigris altaica
Felis catus
Canis lupus familiaris
Odobenus rosmarus divergens
Ailuropoda melanoleuca
Monodelphis domestica
0.02
Euarchontoglires
Laurasiatheria

E IIx Motor

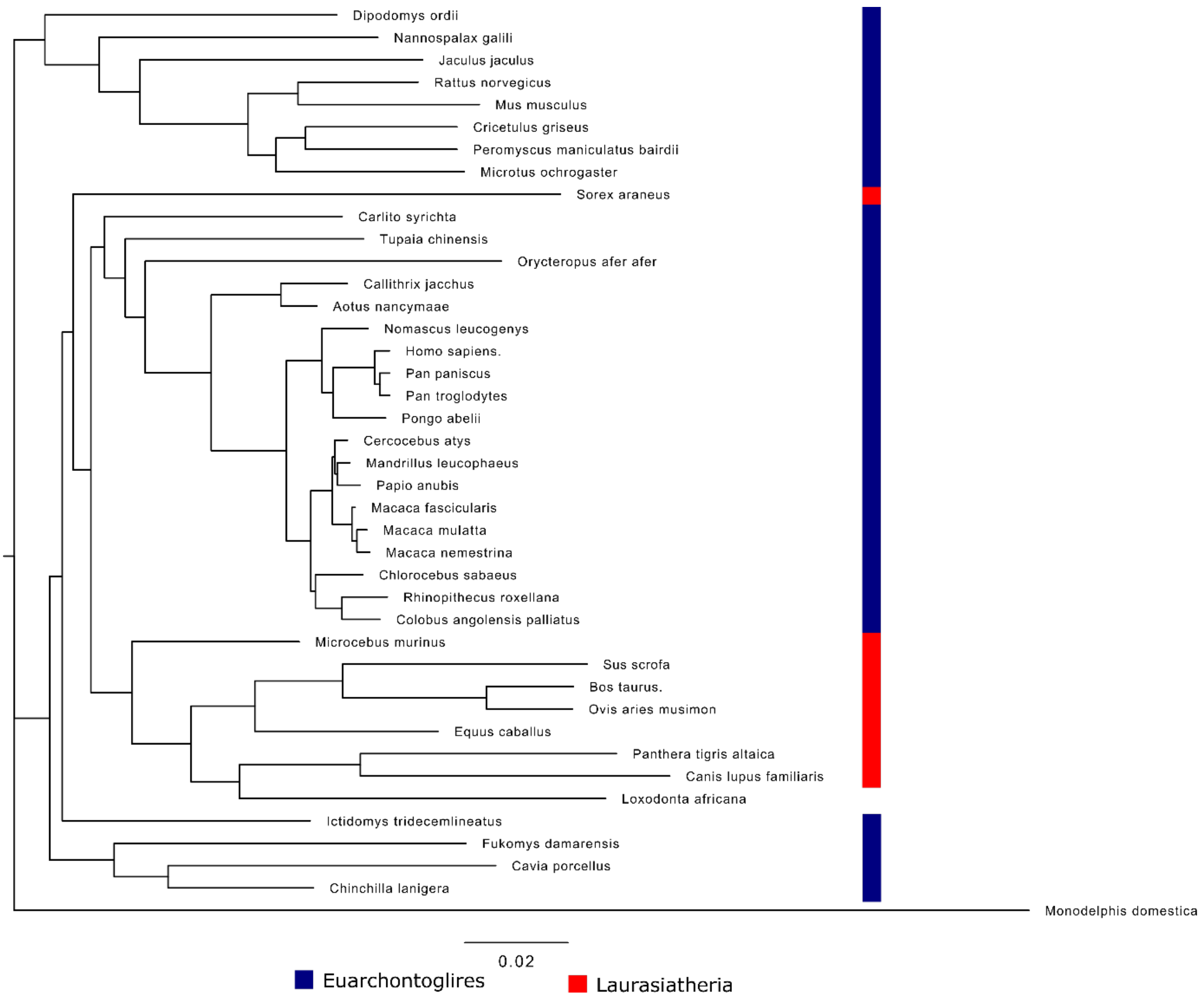
Dipodomys ordii
Nannospalax galili
Jaculus jaculus
Rattus norvegicus
Mus musculus
Cricetulus griseus
Peromyscus maniculatus bairdii
Microtus ochrogaster
Sorex araneus
Carlito syrichta
Tupaia chinensis
Orycteropus afer afer
Callithrix jacchus
Aotus nancymaae
Nomascus leucogenys
Homo sapiens.
Pan paniscus
Pan troglodytes
Pongo abelii
Cercocebus atys
Mandrillus leucophaeus
Papio anubis
Macaca fascicularis
Macaca mulatta
Macaca nemestrina
Chlorocebus sabaeus
Rhinopithecus roxellana
Colobus angolensis palliatus
Microcebus murinus
Sus scrofa
Bos taurus.
Ovis aries musimon
Equus caballus
Panthera tigris altaica
Canis lupus familiaris
Loxodonta africana
Ictidomys tridecemlineatus
Fukomys damarensis
Cavia porcellus
Chinchilla lanigera
Monodelphis domestica
0.02
Euarchontoglires
Laurasiatheria

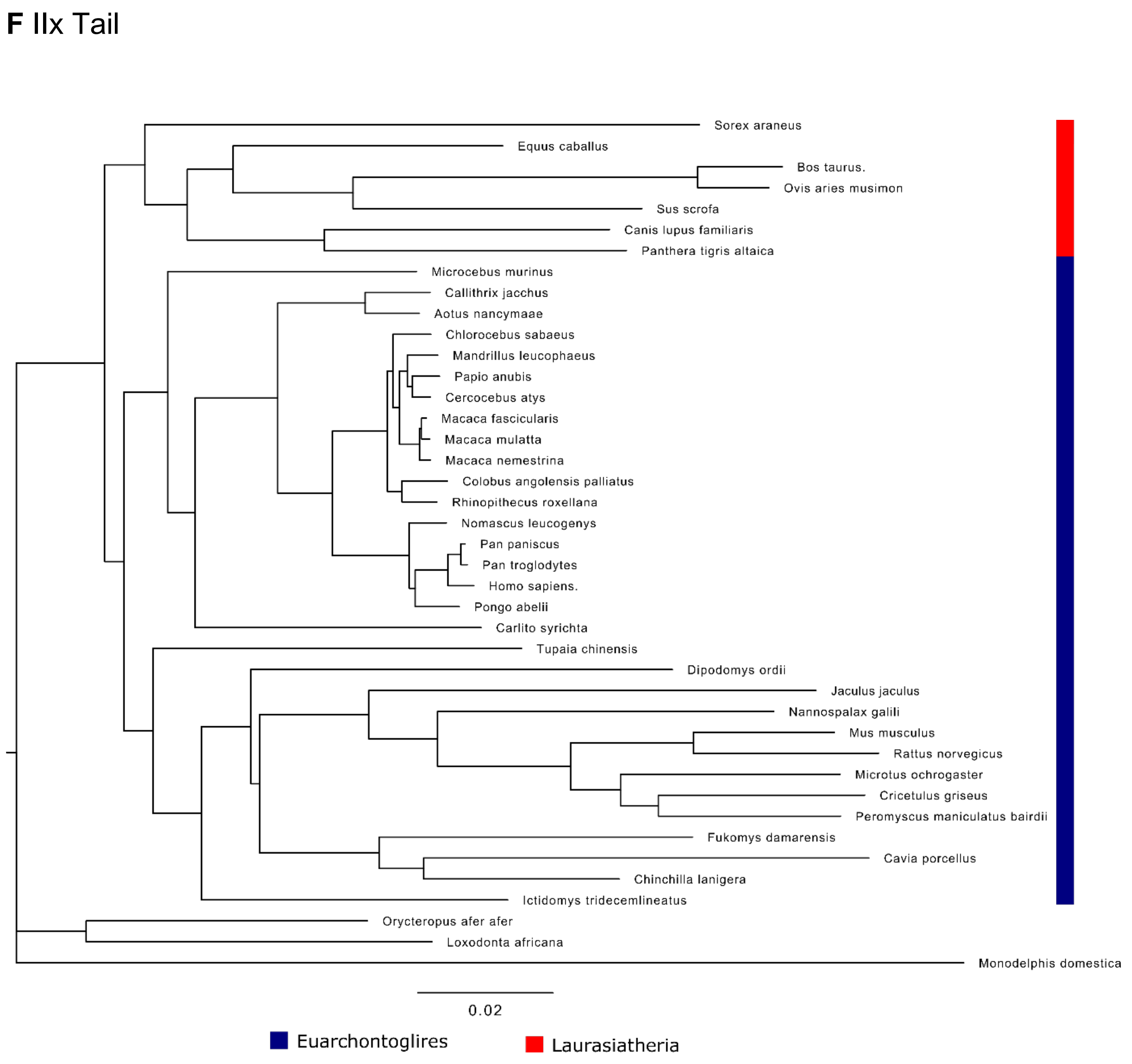
F IIx Tail
Sorex araneus
Equus caballus
Bos taurus.
Ovis aries musimon
Sus scrofa
Canis lupus familiaris
Panthera tigris altaica
Microcebus murinus
Callithrix jacchus
Aotus nancymaae
Chlorocebus sabaeus
Mandrillus leucophaeus
Papio anubis
Cercocebus atys
Macaca fascicularis
Macaca mulatta
Macaca nemestrina
Colobus angolensis palliatus
Rhinopithecus roxellana
Nomascus leucogenys
Pan paniscus
Pan troglodytes
Homo sapiens.
Pongo abelii
Carlito syrichta
Tupaia chinensis
Dipodomys ordii
Jaculus jaculus
Nannospalax galili
Mus musculus
Rattus norvegicus
Microtus ochrogaster
Cricetulus griseus
Peromyscus maniculatus bairdii
Fukomys damarensis
Cavia porcellus
Chinchilla lanigera
Ictidomys tridecemlineatus
Orycteropus afer afer
Loxodonta africana
Monodelphis domestica
0.02
Euarchontoglires
Laurasiatheria

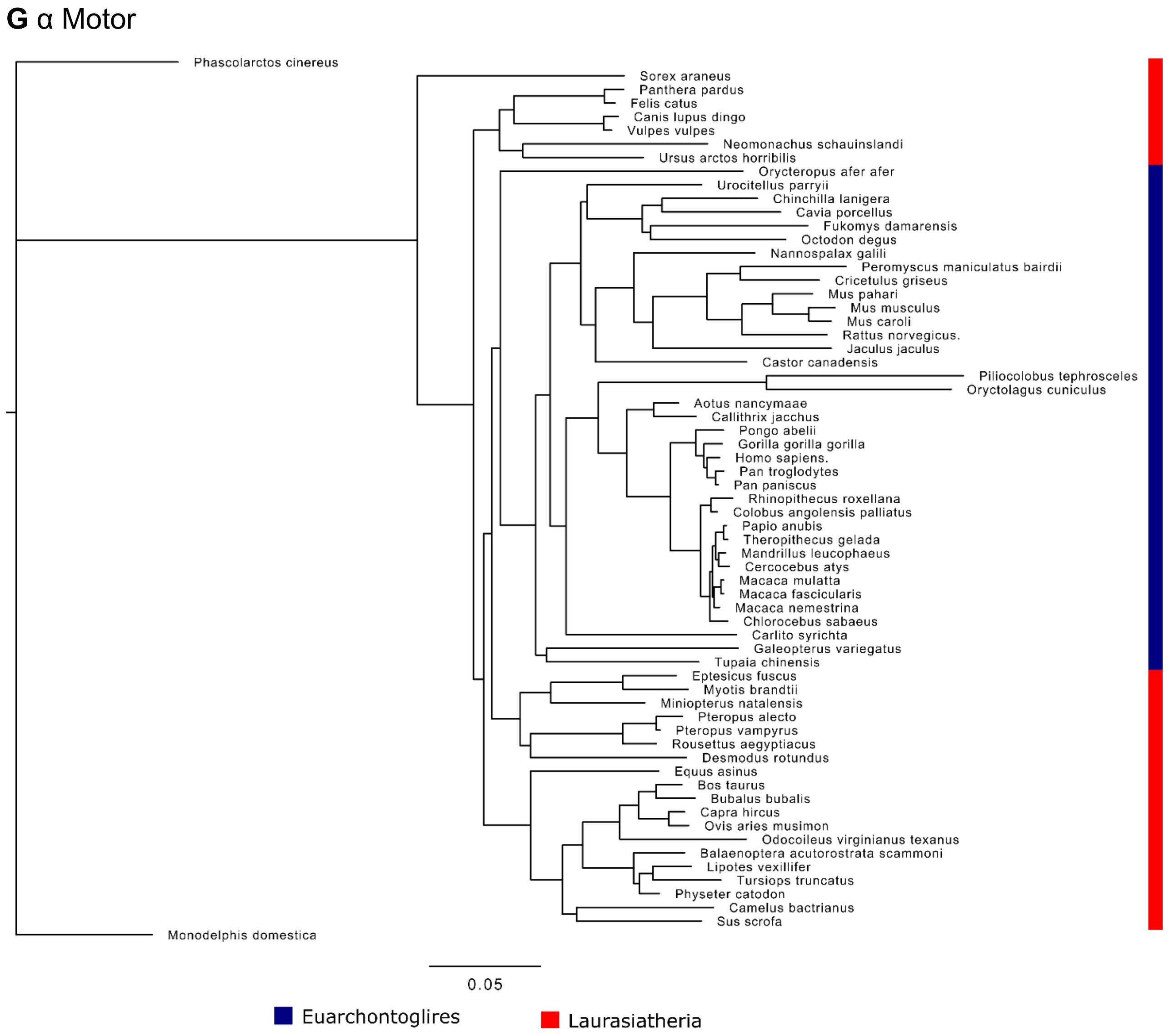
G α Motor
Phascolarctos cinereus
Sorex araneus
Panthera pardus
Felis catus
Canis lupus dingo
Vulpes vulpes
Neomonachus schauinslandi
Ursus arctos horribilis
Orycteropus afer afer
Urocitellus parryii
Chinchilla lanigera
Cavia porcellus
Fukomys damarensis
Octodon degus
Nannospalax galili
Peromyscus maniculatus bairdii
Cricetulus griseus
Mus pahari
Mus musculus
Mus caroli
Rattus norvegicus.
Jaculus jaculus
Castor canadensis
Piliocolobus tephrosceles
Oryctolagus cuniculus
Aotus nancymaae
Callithrix jacchus
Pongo abelii
Gorilla gorilla gorilla
Homo sapiens.
Pan troglodytes
Pan paniscus
Rhinopithecus roxellana
Colobus angolensis palliatus
Papio anubis
Theropithecus gelada
Mandrillus leucophaeus
Cercocebus atys
Macaca mulatta
Macaca fascicularis
Macaca nemestrina
Chlorocebus sabaeus
Carlito syrichta
Galeopterus variegatus
Tupaia chinensis
Eptesicus fuscus
Myotis brandtii
Miniopterus natalensis
Pteropus alecto
Pteropus vampyrus
Rousettus aegyptiacus
Desmodus rotundus
Equus asinus
Bos taurus
Bubalus bubalis
Capra hircus
Ovis aries musimon
Odocoileus virginianus texanus
Balaenoptera acutorostrata scammoni
Lipotes vexillifer
Tursiops truncatus
Physeter catodon
Camelus bactrianus
Sus scrofa
Monodelphis domestica
0.05
Euarchontoglires
Laurasiatheria

H α Tail

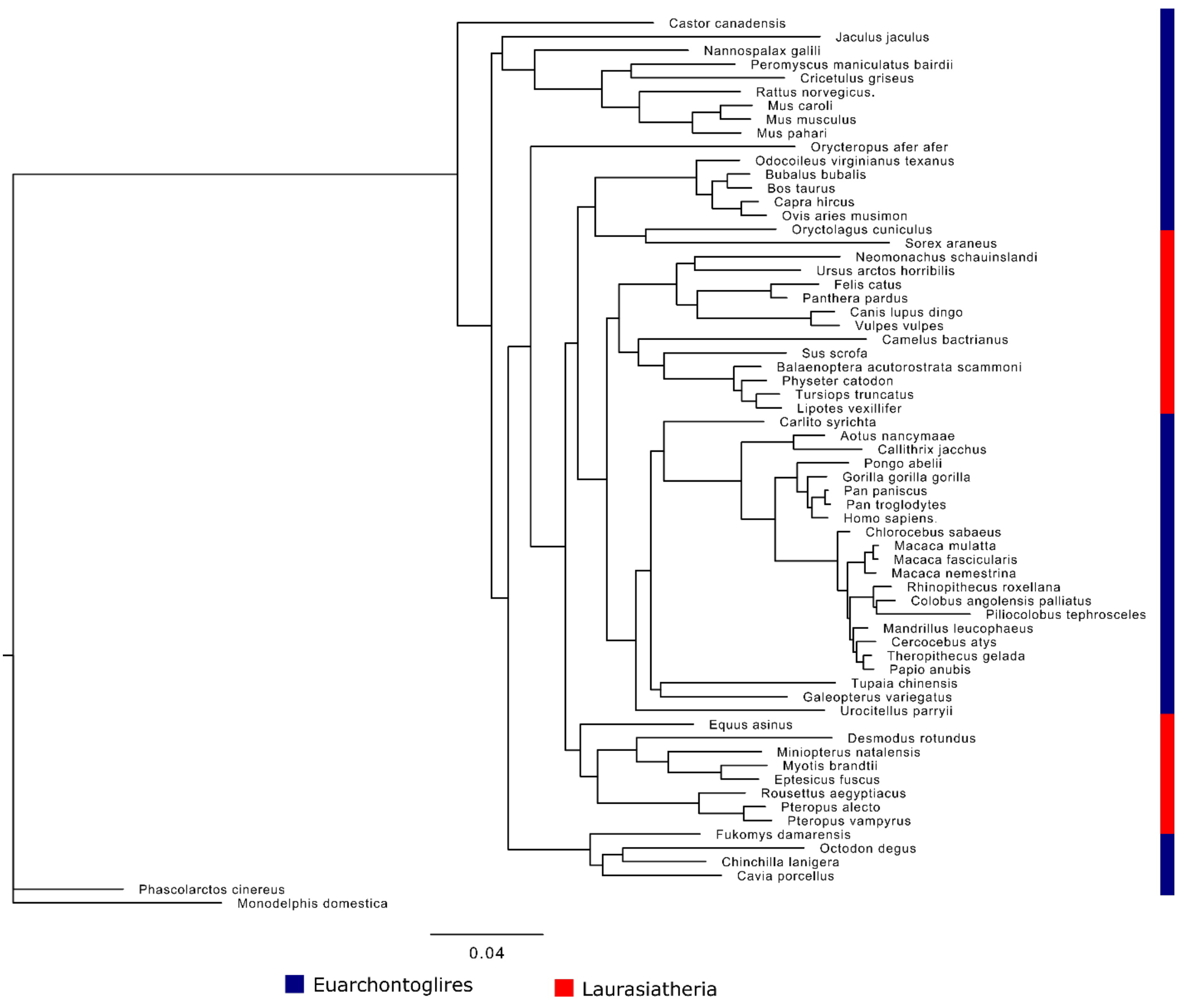
Castor canadensis
Jaculus jaculus
Nannospalax galili
Peromyscus maniculatus bairdii
Cricetulus griseus
Rattus norvegicus.
Mus caroli
Mus musculus
Mus pahari
Orycteropus afer afer
Odocoileus virginianus texanus
Bubalus bubalis
Bos taurus
Capra hircus
Ovis aries musimon
Oryctolagus cuniculus
Sorex araneus
Neomonachus schauinslandi
Ursus arctos horribilis
Felis catus
Panthera pardus
Canis lupus dingo
Vulpes vulpes
Camelus bactrianus
Sus scrofa
Balaenoptera acutorostrata scammoni
Physeter catodon
Tursiops truncatus
Lipotes vexillifer
Carlito syrichta
Aotus nancymaae
Callithrix jacchus
Pongo abelii
Gorilla gorilla gorilla
Pan paniscus
Pan troglodytes
Homo sapiens.
Chlorocebus sabaeus
Macaca mulatta
Macaca fascicularis
Macaca nemestrina
Rhinopithecus roxellana
Colobus angolensis palliatus
Piliocolobus tephrosceles
Mandrillus leucophaeus
Cercocebus atys
Theropithecus gelada
Papio anubis
Tupaia chinensis
Galeopterus variegatus
Urocitellus parryii
Equus asinus
Desmodus rotundus
Miniopterus natalensis
Myotis brandtii
Eptesicus fuscus
Rousettus aegyptiacus
Pteropus alecto
Pteropus vampyrus
Fukomys damarensis
Octodon degus
Chinchilla lanigera
Cavia porcellus
Phascolarctos cinereus
Monodelphis domestica
0.04
Euarchontoglires
Laurasiatheria

I β-Motor

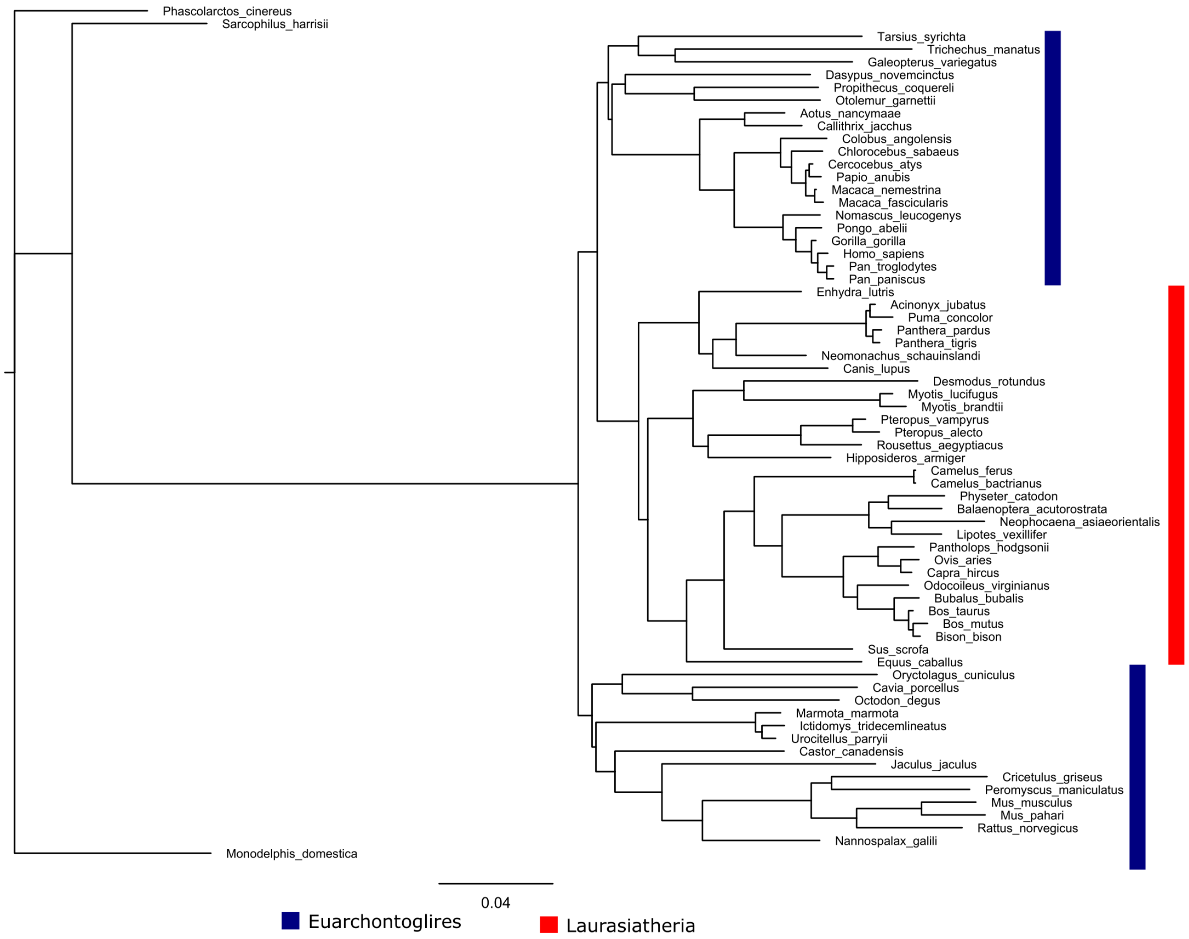
Phascolarctos_cinereus
Sarcophilus_harrisii
Tarsius_syrichta
Trichechus_manatus
Galeopterus_variegatus
Dasypus_novemcinctus
Propithecus_coquereli
Otolemur_garnettii
Aotus_nancymaae
Callithrix_jacchus
Colobus_angolensis
Chlorocebus_sabaeus
Cercocebus_atys
Papio_anubis
Macaca_nemestrina
Macaca_fascicularis
Nomascus_leucogenys
Pongo_abelii
Gorilla_gorilla
Homo_sapiens
Pan_troglodytes
Pan_paniscus
Enhydra_lutris
Acinonyx_jubatus
Puma_concolor
Panthera_pardus
Panthera_tigris
Neomonachus_schauinslandi
Canis_lupus
Desmodus_rotundus
Myotis_lucifugus
Myotis_brandtii
Pteropus_vampyrus
Pteropus_alecto
Rousettus_aegyptiacus
Hipposideros_armiger
Camelus_ferus
Camelus_bactrianus
Physeter_catodon
Balaenoptera_acutorostrata
Neophocaena_asiaeorientalis
Lipotes_vexillifer
Pantholops_hodgsonii
Ovis_aries
Capra_hircus
Odocoileus_virginianus
Bubalus_bubalis
Bos_taurus
Bos_mutus
Bison_bison
Sus_scrofa
Equus_caballus
Oryctolagus_cuniculus
Cavia_porcellus
Octodon_degus
Marmota_marmota
Ictidomys_tridecemlineatus
Urocitellus_parryii
Castor_canadensis
Jaculus_jaculus
Cricetulus_griseus
Peromyscus_maniculatus
Mus_musculus
Mus_pahari
Rattus_norvegicus
Nannospalax_galili
Monodelphis_domestica
0.04
Euarchontoglires
Laurasiatheria

J β-Tail

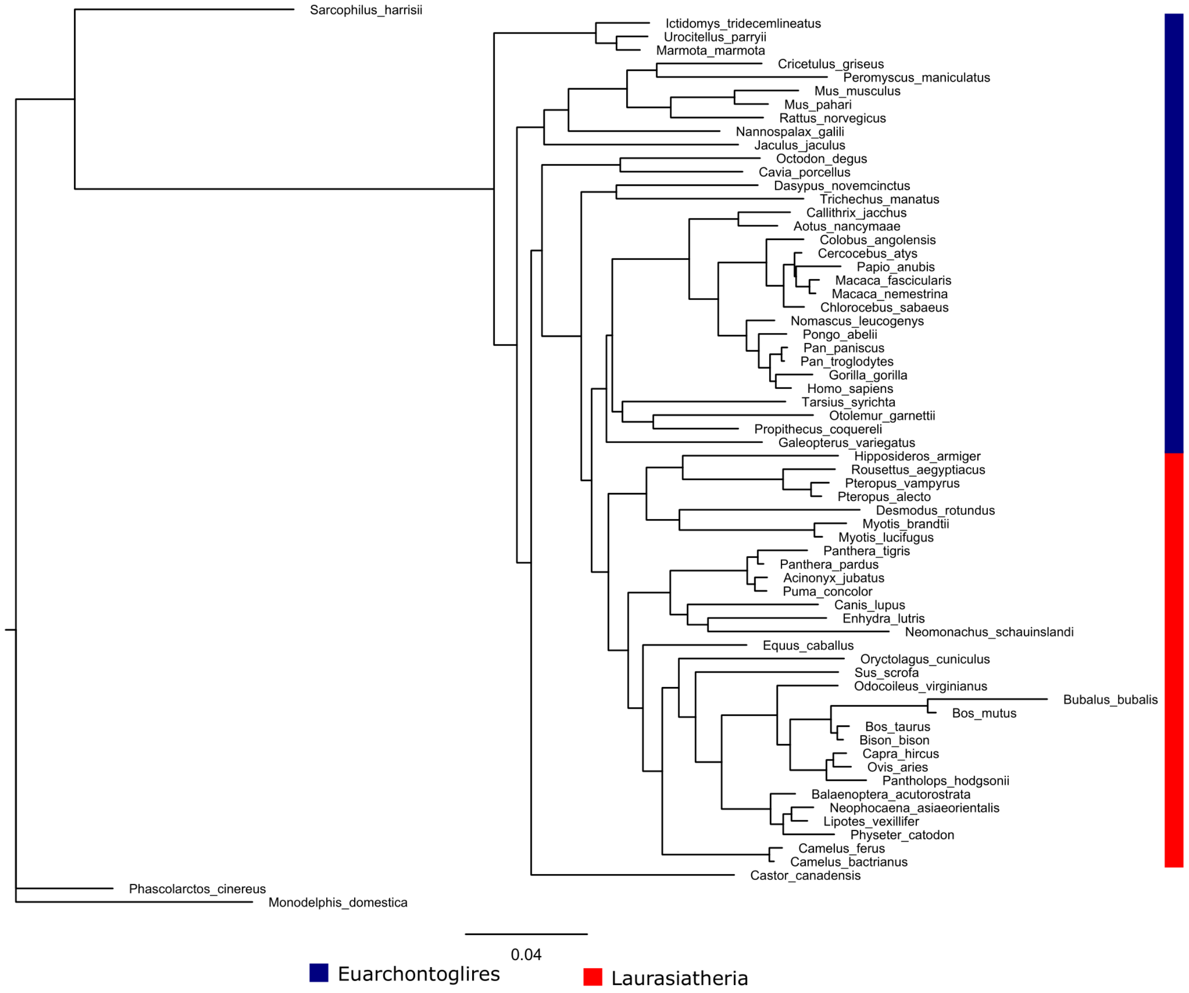

Sarcophilus_harrisii
Ictidomys_tridecemlineatus
Urocitellus_parryii
Marmota_marmota
Cricetulus_griseus
Peromyscus_maniculatus
Mus_musculus
Mus_pahari
Rattus_norvegicus
Nannospalax_galili
Jaculus_jaculus
Octodon_degus
Cavia_porcellus
Dasypus_novemcinctus
Trichechus_manatus
Callithrix_jacchus
Aotus_nancymaae
Colobus_angolensis
Cercocebus_atys
Papio_anubis
Macaca_fascicularis
Macaca_nemestrina
Chlorocebus_sabaeus
Nomascus_leucogenys
Pongo_abelii
Pan_paniscus
Pan_troglodytes
Gorilla_gorilla
Homo_sapiens
Tarsius_syrichta
Otolemur_garnettii
Propithecus_coquereli
Galeopterus_variegatus
Hipposideros_armiger
Rousettus_aegyptiacus
Pteropus_vampyrus
Pteropus_alecto
Desmodus_rotundus
Myotis_brandtii
Myotis_lucifugus
Panthera_tigris
Panthera_pardus
Acinonyx_jubatus
Puma_concolor
Canis_lupus
Enhydra_lutris
Neomonachus_schauinslandi
Equus_caballus
Oryctolagus_cuniculus
Sus_scrofa
Odocoileus_virginianus
Bubalus_bubalis
Bos_mutus
Bos_taurus
Bison_bison
Capra_hircus
Ovis_aries
Pantholops_hodgsonii
Balaenoptera_acutorostrata
Neophocaena_asiaeorientalis
Lipotes_vexillifer
Physeter_catodon
Camelus_ferus
Camelus_bactrianus
Castor_canadensis
Phascolarctos_cinereus
Monodelphis_domestica
0.04
Euarchontoglires
Laurasiatheria
