# Supplementary material for: Identification of sequence changes in myosin II that adjust muscle contraction velocity
Source: PLoS Biol. 2021 Jun 10;19(6):e3001248. doi: 10.1371/journal.pbio.3001248 (PMC8191873; doi:10.1371/journal.pbio.3001248)
Supplement: S1 Table — The mass of species for each isoform was randomised 1,000 times at 0.8–1.2 times the mass value for each given species. This table shows the range of values produced from this analysis. The values for the motors are shown in A and values for the tails in B. (PDF) [file pbio.3001248.s010.pdf]

## A Motor domain

| Isoform | Error Range | Slope Range | $R^2$- Range | Error STD-DEV | Slope STD-DEV | $R^2$- STD-DEV |
|---|---|---|---|---|---|---|
| ***IIb*** | 0.181 - 0.216 | -0.053 | 0.448 - 0.497 | 0.01 | 0.01 | 0.01 |
| ***IIa*** | 0.095 - 0.101 | -0.025 | 0.426 - 0.461 | 0 | 0 | 0 |
| ***IIx*** | 0.083 - 0.138 | -0.068 | 0.796 - 0.869 | 0.01 | 0.01 | 0.01 |
| ***α*** | 0.267 - 0.326 | -0.066 | 0.339 - 0.42 | 0.01 | 0.01 | 0.01 |
| ***β*** | 0.098 - 0.112 | -0.031 | 0.618 - 0.658 | 0 | 0.01 | 0.01 |
| ***EXOC*** | 0.099 - 0.104 | -0.023 | 0.165 - 0.187 | 0 | 0 | 0 |
| ***slowT*** | 0.058 - 0.061 | -0.011 | 0.039 - 0.05 | 0 | 0 | 0 |
| ***EMB*** | 0.034 - 0.036 | -0.007 | 0.118 - 0.136 | 0 | 0 | 0 |
| ***PERI*** | 0.105 - 0.122 | -0.02 | 0.005 - 0.014 | 0 | 0 | 0 |
| ***NMA*** | 0.034 - 0.037 | -0.007 | 0.027 - 0.038 | 0 | 0 | 0 |
| ***NMB*** | 0.021 - 0.023 | 0.04 - 0.043 | 0.103 - 0.12 | 0 | 0 | 0 |
| ***SM*** | 0.066 - 0.073 | -0.015 | 0.479 - 0.514 | 0 | 0 | 0.01 |
| ***NMC*** | 0.107 - 0.117 | -0.022 | 0.095 - 0.113 | 0 | 0 | 0 |

## B Tail domain

| Isoform | Error Range | Slope Range | $R^2$-Range | Error STD-DEV | Slope STD-DEV | $R^2$- STD-DEV |
|---|---|---|---|---|---|---|
| ***IIb*** | 0.112 - 0.126 | -0.041 | 0.558 - 0.607 | 0 | 0.01 | 0.01 |
| ***IIa*** | 0.104 - 0.112 | -0.025 | 0.337 - 0.371 | 0 | 0 | 0.01 |
| ***IIx*** | 0.076 - 0.127 | -0.037 | 0.831 - 0.862 | 0.01 | 0.01 | 0.01 |
| ***α*** | 0.093 - 0.113 | -0.02 | 0.338 - 0.368 | 0 | 0 | 0 |
| ***β*** | 0.156 - 0.194 | -0.037 | 0.059 - 0.121 | 0.01 | 0.01 | 0.01 |
| ***EXOC*** | 0.19 - 0.2 | -0.043 | 0.204 - 0.225 | 0 | 0.01 | 0 |
| ***slowT*** | 0.058 - 0.062 | -0.011 | 0.013 - 0.021 | 0 | 0 | 0 |
| ***EMB*** | 0.12 - 0.128 | -0.024 | 0.208 - 0.237 | 0 | 0 | 0 |
| ***PERI*** | 0.113 - 0.125 | -0.023 | 0.198 - 0.225 | 0 | 0 | 0 |
| ***NMA*** | 0.058 - 0.064 | -0.011 | 0.135 - 0.154 | 0 | 0 | 0 |
| ***NMB*** | 0.051 - 0.054 | -0.011 | 0.069 - 0.088 | 0 | 0 | 0 |
| ***SM*** | 0.099 - 0.105 | -0.018 | 0.127 - 0.144 | 0 | 0 | 0 |
| ***NMC*** | 0.34 - 0.367 | -0.063 | 0.189 - 0.217 | 0 | 0.01 | 0 |
